# Optophysiology: Illuminating cell physiology with optogenetics

**DOI:** 10.1152/physrev.00021.2021

**Published:** 2022-01-24

**Authors:** Peng Tan, Lian He, Yun Huang, Yubin Zhou

**Affiliations:** ^1^Center for Translational Cancer Research, Institute of Biosciences and Technology, Texas A&M University, Houston, Texas; ^2^Klarman Cell Observatory, Broad Institute of MIT and Harvard, Cambridge, Massachusetts; ^3^Center for Epigenetics and Disease Prevention, Institute of Biosciences and Technology, Texas A&M University, Houston, Texas; ^4^Department of Translational Medical Sciences, College of Medicine, Texas A&M University, Houston, Texas

**Keywords:** cellular physiology, nanophotonics, optogenetics, signal transduction, synthetic biology

## Abstract

Optogenetics combines light and genetics to enable precise control of living cells, tissues, and organisms with tailored functions. Optogenetics has the advantages of noninvasiveness, rapid responsiveness, tunable reversibility, and superior spatiotemporal resolution. Following the initial discovery of microbial opsins as light-actuated ion channels, a plethora of naturally occurring or engineered photoreceptors or photosensitive domains that respond to light at varying wavelengths has ushered in the next chapter of optogenetics. Through protein engineering and synthetic biology approaches, genetically encoded photoswitches can be modularly engineered into protein scaffolds or host cells to control a myriad of biological processes, as well as to enable behavioral control and disease intervention in vivo. Here, we summarize these optogenetic tools on the basis of their fundamental photochemical properties to better inform the chemical basis and design principles. We also highlight exemplary applications of opsin-free optogenetics in dissecting cellular physiology (designated “optophysiology”) and describe the current progress, as well as future trends, in wireless optogenetics, which enables remote interrogation of physiological processes with minimal invasiveness. This review is anticipated to spark novel thoughts on engineering next-generation optogenetic tools and devices that promise to accelerate both basic and translational studies.

## Graphical abstract

**Figure:**
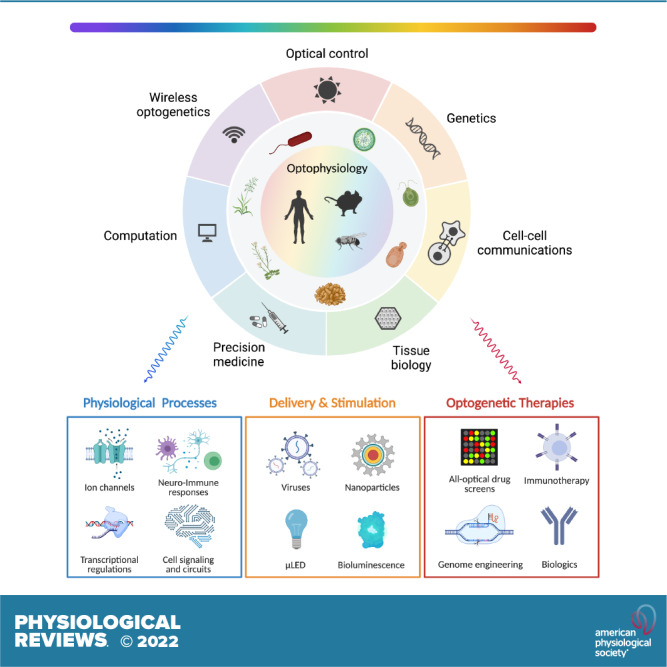

CLINICAL HIGHLIGHTS

1. Optogenetics gives physiologists unprecedented levels of control over protein activity and cell physiology in space and time. This approach shows great promise in facilitating the mechanistic dissection of physiological processes and the development of precision medicine, as exemplified by the design of light-switchable chimeric antigen receptor (CAR) T cell-based immunotherapy against tumors.
2. The combined use of optogenetic actuators and indicators enables high-throughput all-optical drug screens to accelerate the cost-effective discovery of drug candidates, as well as preclinical evaluations of their efficacy and safety.
3. Viral vectors and liposomal nanoparticles have shown great potential for targeted gene therapy and vaccination in humans. These convenient delivery systems are anticipated to unlock bottlenecks that hamper the bench-to-bedside translation of optogenetic tools.
4. The development of red-shifted wireless optogenetic devices has greatly facilitated the noninvasive modulation of cellular physiology in mammals. Optogenetic therapies without surgery and implants have achieved great success in restoring visual functions in patients suffering from retinitis pigmentosa.

## 1. INTRODUCTION

Traditional approaches for studying protein function and physiological processes often involve genetic manipulations of proteins of interest (POIs) or the treatment of cells with agonists or antagonists. These genetic and pharmacological approaches could either lead to irreversible phenotypes in the host cells or elicit undesirable cytotoxicity and signaling cross talk due to off-target or pleiotropic effects. Owing to the superior spatiotemporal precision of light, optogenetics promises to overcome these problems by allowing reversible interrogation of protein activity and cell physiology with high precision. By definition, optogenetics refers to the combination of optics and genetics to enable optical control of physiological processes with tailored function at the molecular, cellular, and systems levels. The term was first coined in 2005 to describe the use of microbial channelrhodopsin-2 (ChR2) to photocontrol neuronal activity (1). Since then, optogenetics has transformed neuroscience by enabling versatile control of neurons and neural circuits in excitable tissues and living organisms (2, 3). The introduction of ChR2-like microbial opsins into neurons allows neuroscientists to photomanipulate action potential with an unprecedented temporal resolution (up to milliseconds) and subcellular precision (2, 4–8).

Over the past decade, optogenetics has been greatly expanded to control a myriad of subcellular targets and physiological processes beyond the nervous system (9–17). An increasing number of genetically encoded photosensory proteins other than ChR2 or its variants have been discovered, as most comprehensively summarized in OptoBase, a web-based platform for documenting molecular optogenetics tools (18). These non-opsin-based photosensors and photoswitches are capable of absorbing photons emitting in the wide range of 300–800 nm to initiate photochemical reactions and trigger changes in protein behaviors, thereby translating user-defined light stimuli into tailored functional outputs (19). These photosensitive modules have been widely engineered into proteins of interest (POIs) to achieve light-inducible protein self-multimerization, protein-protein/target heterodimerization, conformational switch, and protein-target dissociation, thereby mimicking many of the cellular signaling events that are initiated or terminated by natural signals.

Optogenetics allows physiologists to precisely and reversibly perturb POIs and observe the direct functional consequences, ranging from receptor activation in the plasma membrane (PM) to gene expression within the nucleus, in a user-defined time frame at the single-cell level that was previously impossible (17). The coupling of genetically encoded photosensitive actuators with biosensors has enabled closed-loop optogenetics with real-time light stimulation, simultaneous behavioral and physiological activity recording, and feedback control (20). Moreover, multicolor optogenetic stimulation can be exploited as mimics of multiple environmental cues that allow complex manipulations with multidimensional outputs (21). Moving toward in vivo applications, several pioneering approaches have been exploited to overcome the tissue penetration issue associated with most existing blue or green light-activatable optogenetic tools. The microLED (µLED)-based implantable device has been invented to permit the remote actuation of cell circuits in a given tissue environment across space and time (20). Furthermore, the coupling of optogenetics with nanomaterials or bioluminescence has been explored as alternative solutions for wireless optogenetics. These creative approaches obviate the need of fiber optics or µLED implantation in living animals (17, 22–26). Undoubtedly, optogenetics has been benefiting both basic and translational studies by offering novel means to tackle physiological questions that are otherwise inaccessible or less effective with conventional techniques and methods.

Given that microbial opsin-based optogenetics and its applications in neuroscience have been extensively reviewed elsewhere (2, 4–7), we put particular emphasis on nonopsin optogenetic tools (or opsin-free optogenetics), summarizing their photochemical properties, design principles, and exemplary applications in controlling signal transduction and cell physiology. In parallel, we present problems facing optogenetics that hamper its application in deeply buried tissues and further discuss potential solutions by combining optogenetics with microoptics, bioluminescence, and nanotechnologies. It is our hope that this review will spark new thoughts to evolve next-generation optogenetics and accelerate the pace of illuminating cell physiology and developing novel disease intervention strategies.

## 2. CHEMICAL BASIS FOR PHOTORESPONSIVENESS

**FIGURE 1.**
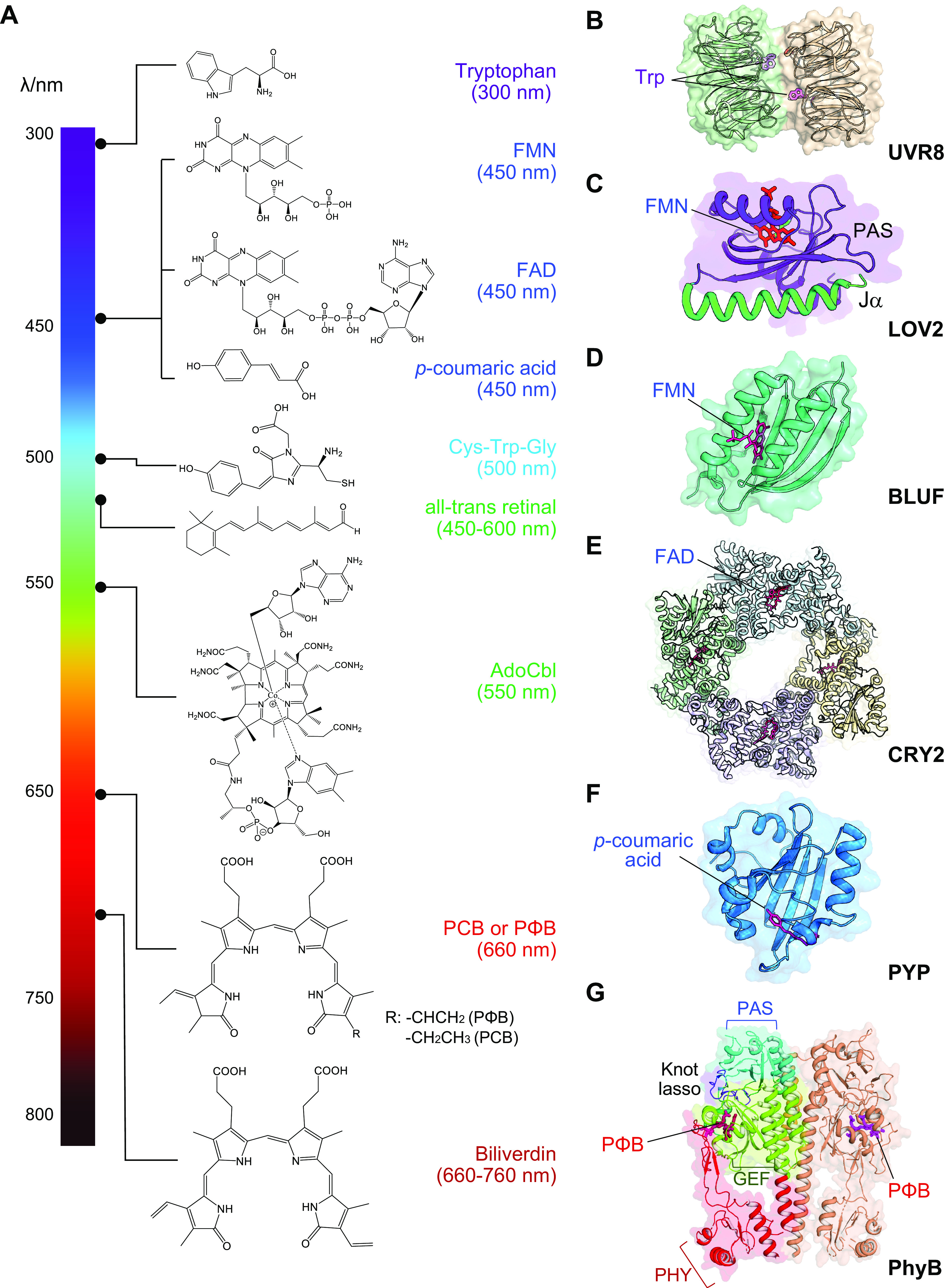
Overview of the optogenetic systems. *A*: chemical structures of photosensing chromophores or cofactors and their photoresponsiveness at varying wavelengths (λ/nm). The black dots indicate the approximate peak excitation wavelengths for each chromophore or cofactor. *B–G*: 3-dimensional structures of representative photosensory domains. The bound cofactors or photosensing antennas are presented as red sticks, which include tryptophan residues for UVR8 [*B*; Protein Data Bank (PDB) entry: 4DNW]; FMN for LOV2 (*C*; PDB entry: 2V0W); FMN for BLUF (*D*; PDB entry: 2IYI); FAD for CRY2 (*E*; PDB entry: 6M79); *p*-coumaric acid for PYP (*F*; PDB entry: 5HD3); and PΦB or PCB for PhyB (*G*; PDB entry: 4OUR). See glossary for abbreviations.

Nature has evolved various organisms across the tree of life, including plants, algae, bacteria, fungi, and corals, to serve as rich sources of photoreceptors. Photoreceptors are capable of absorbing photons emitting in the wide range between 300 nm (ultraviolet; UV) and 800 nm (near-infrared light; NIR) to trigger photochemical reactions, leading to conformational changes in the photosensitive domains that can be relayed to the linked effector domains in POIs. Photon absorption could occur either through intrinsic tryptophan antennas used by the *Arabidopsis* UV-B resistance receptor UVR8 (27) or by chromophores bound to photoreceptors (9, 10, 17). Based on the chemical basis of the chromophores, natural and synthetic photosensory proteins can be divided into seven major categories (FIGURE 1*A* and TABLE 1): tryptophan antennas (from UVR8), flavin adenine dinucleotide (FAD) or flavin mononucleotide (FMN) [light-oxygen-voltage sensing domain (LOV) sensors of blue light using FAD (BLUF) and cryptochrome (CRY)], *p*-coumaric acid [photoactive yellow protein (PYP)], retinal or vitamin A derivatives (from microbial and vertebrate opsins), the Cys-Trp-Gly triad (DronpaN), cobalamin or vitamin B_12_ (cobalamin-binding domains, or CBDs), and bilin analogs (phytochromes).

**Table 1.** Summary of properties and working mechanisms of commonly used PSMs

Cofactor	Domain	PSM	Binding Partner	Mechanisms of Action	Excitation λ, nm	Reversion λ, nm	Selected References
*Tryptophan antennas*							
Trp	UVR	UVR8	UVR8	Dissociation	∼300	Dark	(182, 183)
Trp	UVR	UVR8	COP1	Heterodimerization	∼300	Dark	(27, 140)
*FMN and FAD as vitamin B_2_ derivatives*							
FMN	LOV	LOV2 or cpLOV2		Allosteric regulation	∼450	Dark	(23, 116)
FMN	LOV-STAS	YtvA-LOV	YtvA-LOV	Allosteric regulation	∼450	Dark	(133)
FMN	LOVTRAP	LOV2 or cpLOV2	Zdk1/2	Dissociation	∼450	Dark	(23, 184)
FMN	LOV-HTH	RsLOV	RsLOV	Dissociation	∼450	Dark	(188)
FMN	TULIP	LOVpep	ePDZ	Heterodimerization	∼450	Dark	(147)
FMN	iLID/cpLID	LOV2-ssrA or ssrA-cpLOV2	SspB	Heterodimerization	∼450	Dark	(23, 153)
FMN	LOV-HTH	EL222	DNA	Homodimerization	∼450	Dark	(166)
FMN	LOV-FKF1	FKF1	GIGANTEA	Heterodimerization	∼450	Dark	(168)
FMN	LOV-PB	OptoPB	Lipid	Lipid binding	∼450	Dark	(160, 161)
FMN	RGS-LOV	BcLOV4	Lipid	Lipid binding	∼450	Dark	(164, 165)
FMN	bZIP-LOV	VfAU1-LOV or ptAU1a-LOV	VfAU1-LOV or ptAU1a-LOV	Homodimerization	∼450	Dark	(48, 138, 139)
FAD	Ncap-LOV	VVD+	VVD-	Homodimerization	∼450	Dark	(354)
FAD	Ncap-LOV	pMag	nMag	Heterodimerization	∼450	Dark	(157)
FAD	BLUF	PixD	PixE	Dissociation	∼450	Dark	(53, 185)
FMN	BLUF	PAC		cAMP production	∼450	Dark	(240, 427)
FAD	Cryptochrome	CRY2	CRY2	Self-oligomerization	∼450	Dark	(68)
FAD	CRY2-ARDPPDL	CRY2clust	CRY2clust	Self-oligomerization	∼450	Dark	(67)
FAD	CRY2 (E490G)	CRY2olig	CRY2olig	Self-oligomerization	∼450	Dark	(136)
FAD	Cryptochrome	CRY2	CIB1	Heterodimerization	∼450	Dark	(63, 65)
*p-Coumaric acid (or 4-hydroxycinnamic acid)*							
*p*-Coumaric acid	Fluorescent protein	PYP		Allosteric regulation	∼450	Dark	(114, 115)
*Retinal and vitamin A derivatives*							
Retinal	Rhodopsin	ChR2		Cation channel	∼470	Dark	(74)
Retinal	Rhodopsin	RO4		G_α_io	∼470	Dark	(76)
Retinal	Rhodopsin	OPN4		G_α_q	∼470	∼560	(80, 428)
Retinal	Rhodopsin	JellyOP		G_α_s	∼493	Dark	(77, 78)
Retinal	Rhodopsin	Opto-XR		Gq, Gs, Gi	∼500	Dark	(82)
Retinal	Rhodopsin	ChRmine		Cation channel	∼585	Dark	(425, 426)
Retinal	Rhodopsin	Chrimson		Cation channel	∼590	Dark	(424)
Retinal	Rhodopsin	bReaChES		Cation channel	∼600-650	Dark	(429, 430)
*Cys-Trp-Gly*							
*Cys-Trp-Gly*	Fluorescent protein	Dronpa145K	Dronpa145N	Dissociation	∼500	∼400	(87)
*Cys-Trp-Gly*	Fluorescent protein	Dronpa145N	Dronpa145N	Dissociation	∼500	∼400	(87)
*Cys-Trp-Gly*	Fluorescent protein	pdDronpa1	pdDronpa1	Dissociation	∼500	∼400	(88)
*Cobalamin or vitamin B_12_*							
Cbl	Cobalamin-binding domain	MxCBD		Dissociation	∼545	Dark	(93)
Cbl	Cobalamin-binding domain	TtCBD		Dissociation	∼545	Dark	(93)
*Bilin and its derivatives*							
PCB	Cyanobacteriochrome	Am1	BAm green	Heterodimerization	∼525	∼680	(178)
PCB	Cyanobacteriochrome	Am1	BAm red	Heterodimerization	∼680	∼525	(178)
PCB	Phytochrome	CPH1		Homodimerization	∼660	∼740	(109)
PΦB	Phytochrome	PhyA	FHY1 or FHL	Heterodimerization	∼660	∼740	(175)
PΦB	Phytochrome	PhyB	PIF	Heterodimerization	∼650	∼750	(103, 106)
BV	Phytochrome	BphP1	PpsR2 orQ-PAS1	Heterodimerization	∼760	∼640/dark	(102, 180)
BV	Phytochrome	DrBphP1		Homodimerization	∼740	∼650/dark	(191)
BV	Phytochrome	DrBphP	LDB	Heterodimerization	∼660	∼780/dark	(181)
BV	Phytochrome	LAPD		cGMP/cAMP production	∼650	∼750	(243)

See glossary for abbreviations.

### 2.1. Tryptophan Antennas

UV resistance locus 8 (UVR8) derived from *Arabidopsis* is responsive to UV-B light at the wavelength of 280–320 nm. A triad of tryptophan residues (W233/W285/W337) in UVR8 are proposed to function as photon absorbers (28). UVR8 receptor exists as a homodimer in the dark (FIGURE 1*B*), with the tryptophan antennas at the dimeric interface in contact with adjacent residues to stabilize the dimer (29). UV-B light illumination breaks the intersubunit electrostatic interactions, thereby leading to dimer-to-monomer transition of UVR8. Upon UV light-induced monomerization, UVR8 forms a heterodimer with an E3 ubiquitin ligase, the constitutive photomorphogenesis protein 1 (COP1) (30). When UV light illumination ceases, UVR8 returns to its ground state within a few hours, a process facilitated by repressor of UV-B photomorphogenesis 1 and 2 in plants (27, 31). Because UV light irradiation is known as an environmental carcinogen and causes cytotoxicity, UVR8-based tools are mostly limited to cellular studies ex vivo.

### 2.2. FMN and FAD as Vitamin B_2_ Derivatives

FMN and FAD derived from vitamin B_2_, which are readily available in mammalian cells, are often utilized by the LOV, BLUF, and CRY families of flavoprotein as photo-absorbing cofactors. These photoswitchable domains, relatively small in size with 100–150 amino acids, are primarily derived from plant and microbial photoreceptors that respond to blue light in the wavelength of 440–490 nm.

LOV domains belong to the Per-ARNT-Sim (PAS) superfamily and show high sequence homology to motifs involved in sensing environmental cues, such as light, oxygen, and voltage (32, 33). Over 6,700 LOV domains from plants and protists have been identified through bioinformatic analysis on >40 million open reading frames (ORFs) from InterPro and OneKP databases (32). LOV often uses a non-covalently bound cofactor, FMN, to sense blue light. The *Avena sativa* LOV2 (AsLOV2) domain derived from oat phototropin 1 is an intensively studied model photosensory system (34, 35). AsLOV2 contains 110 amino acids and adopts a mixed α/β protein fold (36). Within the cofactor binding pocket, the isoalloxazine ring of FMN is surrounded by a hydrogen-bonding network that senses and responds to photoexcitation. In the dark, the COOH-terminal Jα helix tightly docks to the PAS core of LOV2 (FIGURE 1*C*). Photoexcitation triggers the formation of a covalent photoadduct between C450 and FMN on the microsecond (μs) timescale to cause protonation of the adjacent FMN-N5 position. This photochemical reaction leads to the rotation of a nearby glutamine residue (Q513) to unwind the Jα helix, culminating in the unfolding and subsequent undocking of the Jα helix from the PAS core (34, 35, 37). This process is fully reversible, with an activation half-life of 5–30 s and an estimated deactivation half-life of ∼0.5–1 min. Notably, the deactivation half-time can be further tuned to range from seconds to up to half an hour via the introduction of environment-sensitive mutations into LOV2 (38–41). Two prominent mutants, C450A and I539E, are often used to mimic the dark and lit states of LOV2 in optogenetic applications (42, 43). The free energy difference between the two states is estimated to be ∼3.8 kcal/mol (43).

Other notable riboflavin-dependent LOV proteins sharing similar structural features include *1*) the *Arabidopsis thaliana* phototropin that contains two LOV domains (AtLOV1 and AtLOV2) in an NH_2_-terminal photosensory input region coupled to a COOH-terminal effector serine/threonine kinase motif (44); *2*) stress response protein YtvA with a LOV-STAS (sulfate transporter anti-sigma-factor antagonist) domain from *Bacillus subtilis* (45); *3*) VIVID (VVD) from the filamentous fungus *Neurospora crassa* (46); *4*) EL222, a light-activated DNA-binding protein with DNA binding helix-loop-helix (HLH) domain from *Erythrobacter litoralis* (47); *4*) the basic region/leucine zipper (bZIP) transcription factor aureochrome from *Vaucheria frigida* (vfAU1-LOV, or AuLOV as abbreviation) (48); and *6*) flavin-binding Kelch repeat F-box1 (FKF1) from *A. thaliana* (49). These LOV-bearing photosensory proteins propagate signals either through coupling LOV with a linked auxiliary effector domain or via light-dependent heterotypic interactions with their corresponding binding partners.

BLUF domain adopts a typical ferredoxin-like topology and uses FAD or FMN as a cofactor sandwiched between two α-helices (FIGURE 1*D*). Blue light causes hydrogen bond rearrangement between the N5 and O4 positions of FAD and the conserved Tyr/Gln side chains, triggering conformational changes across the β-sheet with subsequent activation of the linked effector domain (50). Representative BLUF-containing proteins include *1*) AppA from the purple bacterium *Rhodobacter sphaeroides* that modulates the DNA-binding activity of the transcription factor PpsR to control photosynthetic gene expression (51, 52); *2*) the cyanobacterial protein PixD (or Slr1694) involved in regulating the phototaxis of *Synechocystis sp.* strain PCC 6803 (53); *3*) the *Escherichia coli* protein YcgF, which controls biofilm formation in an extrahost environment (54, 55); and *4*) photoactivated adenylyl cyclases (PACs), including euPAC from *Euglena gracilis*, bPAC from *Beggiatoa*, and OaPAC from *Oscillatoria acuminata*, which contain BLUF domains and catalyze cyclic AMP (cAMP) production. Substitution of the flavin-neighboring tyrosine and glutamine within the BLUF domain was found to abrogate its photochemical reactivity, strongly suggesting the importance of the Tyr–Gln–FAD hydrogen-bonding network (56).

Cryptochromes (CRYs) represent a large family of blue light-responsive flavoproteins involved in regulating plant growth, as well as magnetoreception and circadian rhythm in mammals (57, 58). CRY typically contains an NH_2_-terminal photolyase homology region (PHR) that binds noncovalently to FAD as the chromophore (FIGURE 1*E*) and a less conserved COOH-terminal domain of varying sizes. Blue light illumination causes reduction of FAD to initiate conformational changes that culminate in forming CRY2 homooligomers (59) or engaging its binding partners, such as CRY-interacting bHLH (CIB1) and COP1 (60), through heterotypic interactions. CRY2 or its mutants can be photoactivated in the range of 450–500 nm within seconds and return to the dark state with estimated half-lives of 2.5–24 min (57). In *Arabidopsis*, BIC has been shown to potently inactivate CRY2 and abrogate photoinducible responses (61, 62). Light-triggered homo- and heterodimerization of *A. thaliana* CRY2 (AtCRY2) is widely used in optogenetic applications discussed below (63–68).

### 2.3. p-Coumaric Acid (or 4-Hydroxycinnamic Acid)

The photoactive yellow protein (PYP) from Gram-negative *Halorhodospira* bacteria contains a PAS domain bound with *p*-coumaric acid (or 4-hydroxycinnamic acid; FIGURE 1*F*) to serve as an intramolecular conformational switch (69, 70). Absorption of blue light peaked around 450 nm causes *trans*-to-*cis* isomerization of the chromophore (71–73), leading to protonation of *p*-coumaric acid with the concomitant partial unfolding of PYP to alter its overall structure and affect the linked effector domain. Because rodents and humans lack the biosynthetic machinery for de novo *p*-coumaric acid synthesis, the use of PYP-based optogenetic tools in mammals necessitates the supply of this exogenous compound, which might hamper the application in vivo.

### 2.4. Retinal and Vitamin A Derivatives

Vitamin A-based retinaldehyde chromophore bestows a group of seven transmembrane G protein-coupled receptors (GPCRs) called opsins with light sensitivity (e.g., rhodopsin, sensory protein found in the rod cells of the retina). A series of photosensitive opsins have been discovered and engineered with varying kinetics, dynamics, and peak activation wavelengths that range from blue to red (400–700 nm) (2, 4–8, 74). A typical rhodopsin binds covalently to one retinal molecule through a Schiff base with a lysine residue (such as Lys296 in bovine rhodopsin) in the cofactor binding pocket (75). Photons can induce photoisomerization of 11-*cis*-retinal or 11-*cis*-3,4-didehydroretinol to adopt an all-*trans* conformation. Glu113 stabilizes the protonation of the Schiff linkage between retinal and Lys296. The subsequent conformational change within the opsins induces channel pore dilation to allow ion flux across the PM and alter the membrane action potential in excitable cells and tissues. Reversion to the ground state occurs on the millisecond timescale when the all-*trans*-retinal is hydrolyzed and dissociated from rhodopsin. Aside from rhodopsins, a group of light-activated vertebrate GPCRs are found in animals that engage different intrinsic G protein pathways. Examples include *1*) rat rhodopsin RO4 that activates intrinsic Gi/o (76); *2*) box jellyfish opsin (JellyOp) repetitively activating Gs signaling (77, 78); and *3*) olivary pretectal nucleus 4 (OPN4) or melanopsin that engages Gi- and Gq-related pathways involved in vision restoration (79, 80) and circadian rhythm (81). Because the activation and inactivation kinetics of ectopically expressed OPN4 is >1,000-fold slower than that of ChR2, melanopsin is well suited to control biological events in nonexcitable cells.

In addition to natural photosensory GPCRs, synthetic light-gated GPCRs (Opto-XRs) have been engineered by combining the extracellular and transmembrane domains of rhodopsins with the intracellular loops of ligand-gated GPCRs (82). For instance, Opto-XRs built upon adrenergic receptors enable optical control of Gs-mediated adenylyl cyclase activation or Gq-associated phospholipase C (PLC) activation. A similar hybrid approach has been employed to generate light-sensitive μ-opioid-like receptor (Opto-MOR) based on RO4 (83). When expressed in selected GABAergic neurons, Opto-MOR enables light-inducible suppression of cAMP production, activation of MAPK signaling, and mimicry of classical MOR-mediated opioid circuitry and reward/aversion behaviors.

Microbial opsins have been discovered since the early 1970s, including the light-driven proton-pumping rhodopsin and sensory rhodopsin for microbial light sensing and phototaxis. Rhodopsins functioning as ion transporters have been widely found in microbes, including bacteriorhodopsin, *Acetabularia* rhodopsin, xanthorhodopsin [11], *Gloeobacter* rhodopsin, *Exiguobacterium sibiricum* rhodopsin, proteorhodopsin derived from γ-proteobacteria, fungal retinal protein *Leptosphaeria* rhodopsin, and sodium pump rhodopsin. Halorhodopsin has been discovered in the archaeon *Halobacterium salinarum* acting as a light-driven inward chloride pump. A major difference between microbial opsins and mammalian opsins is reflected in the conformation adopted by the bound cofactor retinal. All-*trans* retinal binds to the microbial opsin and isomerizes to 13-*cis*. By contrast, 11-*cis* retinal reversibly binds to the animal opsins and isomerizes to all-*trans*. In addition, the tertiary structures of microbial retinal proteins, including bacteriorhodopsin and halorhodopsin, share less similarity with those of animal retinal proteins, such as bovine rhodopsin and squid rhodopsin. Nonetheless, the boundary can be blurred via GPCR engineering or evolution. For instance, microbial retinal proteins with the cytoplasmic loop of bovine rhodopsin have been shown to activate the trimeric G protein transducin in a way similar to animal retinal proteins. Furthermore, a microbial opsin middle rhodopsin has 11-*cis* retinal as a chromophore, similar to animal opsins. Recently, animal opsin Opn3 was found to act as a light sensor when reconstituted with 13-*cis* retinal (84).

Similar to the light-sensing (or vision) function of animal opsins, a group of microbial opsins with light sensory functions has been identified to show light avoidance or attractive behavior. The light stimulus is captured by the photoreactive membrane-embedded retinylidene proteins sensory rhodopsin I (SRI) and sensory rhodopsin II (SRII, or phoborhodopsin). In the cell membranes, SRI and SRII form complexes with their cognate transducer proteins, called halobacterial transducer protein for SRI (HtrI) and for SRII (HtrII), respectively. Light signals are transmitted from the SRI-HtrI and SRII-HtrII complexes to a cytoplasmic two-component signal transduction cascade, consisting of the chemotaxis histidine kinase (Che)A and the response regulator CheY, which regulates the rotational direction of the flagellar motor for phototaxis. SRI is a dual photoreceptor that mediates positive phototaxis under 587-nm light and negative phototaxis under harmful near-UV radiation, whereas SRII is activated by blue light and produces only a repellent phototaxis signal (84). Since both microbial and vertebrate opsins are extensively reviewed elsewhere (2, 4–8, 74), we will not elaborate further in this review.

### 2.5. Cys-Trp-Gly

Dronpa is a photoswitchable protein sharing >75% sequence similarity with green fluorescent protein (GFP) (85). The chromophore of Dronpa is formed by the Cys62-Tyr63-Gly64 tripeptide that resides in a central α-helical segment and is enclosed by an 11-stranded β-barrel. The chromophore triad primarily adopts a *cis* conformation in the deprotonated on state. Upon photostimulation at 500 nm, protonated Cys-Tyr-Gly of Dronpa assumes a *trans* configuration and becomes nonfluorescent in its off state, which also leads to the partial unfolding of the β-barrel to disrupt the oligomeric interface (86). A mutant form of Dronpa, DronpaN (K145N), is engineered to undergo a tetramer-to-monomer transition when the light is switched from 400 nm to 500 nm (87). A dimeric version, designated pdDronpa1, has been further evolved to have higher photosensitivity and brightness. pdDronpa1 dissociates into a monomer in the presence of cyan light (∼500 nm) and reassembles as a dimer with a half-life of ∼30 min upon violet light stimulation at ∼400 nm (88). Dronpa variants represent a class of unique photoswitchable domains that do not require additional chemical cofactors.

### 2.6. Cobalamin or Vitamin B_12_

Cobalamin or Vitamin B_12_ is an adenosyl- or methyl-donating cofactor for many archaeal and prokaryotic enzymes, including isomerase, methyltransferase, and reductase (89). Cobalamin-binding domains, found in CarH of Gram-negative bacteria *Myxococcus xanthus* (MxCBD) and *Thermus thermophilus* (TtCBD), function as light-sensitive transcriptional repressors of carotenoid synthesis and carotenoid-mediated protection against photooxidative damage (89, 90). The CarH consists of an NH_2_-terminal DNA-binding domain and a COOH-terminal adenosylcobalamin (5′-deoxyadenosylcobalamin, AdoCbl)-binding and oligomerization domain to directly sense light and regulate gene expression (91). In the dark, AdoCbl-bound CarH exists as a tetramer, allowing itself to bind to the carotenoid promoters to repress gene transcription. Green light (545 nm) stimulation causes the cleavage of the 5′-deoxyadenosyl group in the CBD-AdoB_12_ complex, resulting in a tetramer-to-monomer transition of CBD to release CarH-mediated transcriptional repression (92, 93). Photoexcited MxCBD reverts to its tetrameric dark state with an estimated half-life of ∼30 min. Because the cobalamin cofactor in TtCBD forms a covalent adduct with CarH through a bis-His ligation (89, 91), TtCBD-based tools are largely irreversible and thus have limited applicability in probing dynamic physiological processes with fast kinetics.

### 2.7. Bilin and Its Derivatives

Bilin and its derivatives are tetrapyrrole chromophores bound to phytochromes to serve as photon absorbers and shift the photoactivation window to the red or infrared end of the light spectrum (600–900 nm) (94–98). Three bilin-derived chromophores are commonly found in phytochromes, including phytochromobilin (PΦB) in plants, phycocyanobilin (PCB) in cyanobacteria or algae, and biliverdin (BV) in bacteria. Phytochrome typically photoswitches between two distinct conformations (99–101), the red light-absorbing Pr state and far-red light-responsive Pfr state. The double bond between the C and D rings of the chromophore undergoes light-dependent isomerization, leading to an allosteric transition between the Pr and Pfr states (97, 102, 103). Three modular domains are found in the NH_2_-terminal photosensory module (PSM) of phytochrome (FIGURE 1*G*), including Period/Arnt/SIM (PAS), cGMP phosphodiesterase/adenylyl cyclase/FhlA (GAF), and a phytochrome-specific domain (PHY). The COOH-terminal effector region or output module (OPM) varies among different phytochromes but often contains a histidine kinase-related domain (HKRD) for dimerization and nuclear localization.

PΦB-bound *Arabidopsis* phytochrome B (PhyB) absorbs red light (640–670 nm) to assume an activated Pfr state and physically interacts with phytochrome-interacting factors (PIFs), such as PIF3 and PIF6 (104–107). This process is readily reversed upon far-red light illumination at 700–760 nm with a half-life of <5 s (103, 108). The cyanobacterial phytochrome 1 (CPH1) uses PCB as the chromophore and exhibits reversible dimer-to-monomer transition when the light is switched from 660 nm to 740 nm (109). The bacteriophytochrome photoreceptor 1 (BphP1) from *Rhodopseudomonas palustris* uses biliverdin, a metabolite that is abundantly present in mammals, as the chromophore. BphP1 is also activated with infrared light at 760 nm to enable its heterodimerization with RpsR2. After red light irradiation at 640 nm or when kept in the dark, BphP1 gradually relaxes back to an inactive Pr state (102).

Phytochrome’s advantages in optogenetic applications are rooted in its ability to absorb red-shifted light. First, the red photo-sensing window allows paralleled use of bilin-bound phytochromes with blue or green light-activatable optogenetic tools. The wide spectral difference enables orthogonal control of multiple targets in a single experiment. Second, because far-red and NIR light can deeply penetrate biological tissues (up to cm), phytochrome-based molecular tools entail wireless in vivo optogenetics without the use of invasive optical fibers. However, mammals are incapable of synthesizing PΦB or PCB, which necessitates the additional expression of multiple bacterial enzymes, such as the SynPCB platform (110), to reconstitute the involved biosynthesis pathways in mammalian cells.

## 3. OVERVIEW OF PHOTOSENSORY MODULES

**FIGURE 2.**
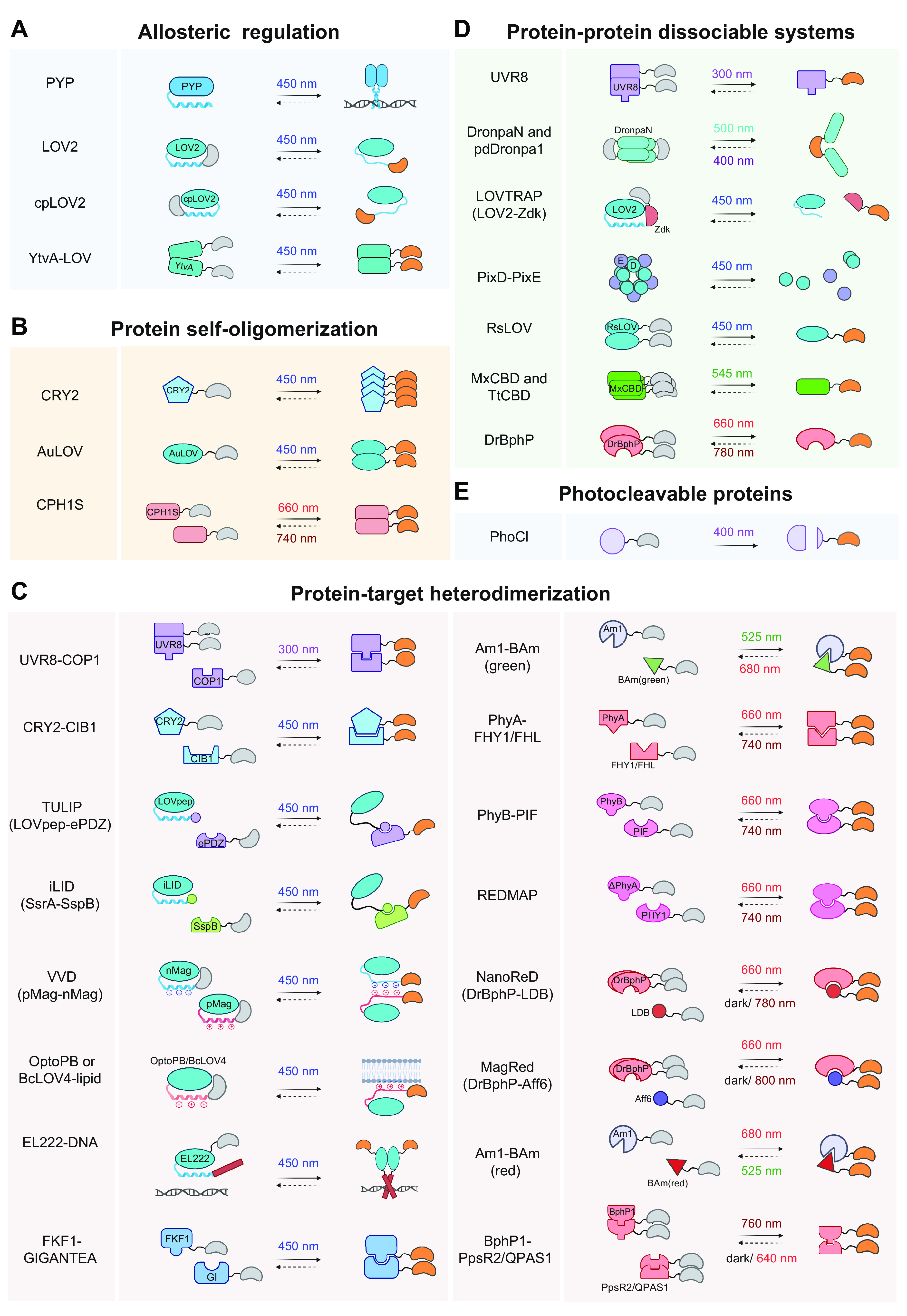
Classification of photosensory modules based on photoregulatory mechanisms and engineering strategies for optogenetic control of POIs. *A*: allosteric regulation. *B*: protein self-oligomerization. *C*: protein-target heterodimerization. *D*: protein-protein dissociable systems. *E*: photocleavable proteins. Gray indicates an inactive POI, and orange represents a POI in its active state. See glossary for abbreviations. Image created with BioRender.com and used with permission.

In this section, we summarize the working mechanisms of representative photosensory modules (PSMs) and discuss generalizable principles of optogenetic engineering to control the behavior of POIs in mammalian cells (FIGURE 2 and TABLE 1). PSMs can be modularly inserted into host protein scaffolds to achieve allosteric control of protein activity. These modular domains can also be fused with POIs to optically recapitulate protein self-oligomerization, protein-target heterodimerization, and protein-target dissociation. Alternatively, photoinducible PSM self-cleavage can be utilized to confer optogenetic control of target proteins and their associated physiological processes. To avoid redundancy, we present the first or most notable proof-of-concept demonstrations for each PSM.

### 3.1. Allosteric Regulation

Allosteric control is a classic mechanism shared by many signaling proteins and enzymes for tunable control of protein function (111–113). Allostery refers to the process whereby ligand or effector binding to a regulatory site induces conformational changes to affect the behavior of a distinct functional site, thereby causing profound changes in overall protein activity (FIGURE 2*A*). Genetically encoded photoswitches, such as PYP and LOV2 domains, can be engineered into POIs to enable allosteric regulation of biological processes by photons, rather than chemical ligands or effectors.

#### 3.1.1. PYP.

PYP was initially applied to devise a light-controllable DNA binder (114). PYP was fused to the COOH terminus of a homodimeric bZIP protein, GCN4, as a photoswitch (GCN4Δ25PYP-v2) for regulating DNA binding. In the dark, the monomeric hybrid protein was kept in the *trans* folded state (FIGURE 1*F*) to sequester the linked leucine zipper dimerization domain of GCN4 and block DNA binding. At 450 nm, blue light-induced unfolding of PYP unmasked GCN4, permitting its dimerization to cause a twofold increase in cognate DNA binding (114). On the contrary, another truncated GCN4-PYP chimera, designated GCN4(S)Δ25PYP, exhibited light-inducible dissociation from its target DNA (115). Therefore, it is possible to devise both ON and OFF switches with slight modifications on effector-PYP combinations.

#### 3.1.2. LOV2 and cpLOV2.

AsLOV2 derived from *Avena sativa* phototropin 1 is among the most well-characterized and widely used photoswitches (10, 34, 35, 37, 116). In a typical design, an effector domain is fused immediately after the COOH-terminal Jα helix of LOV2 via linker optimization (116), thus caging the effector through steric hindrance imposed by the PAS core of LOV2 in the dark state (FIGURE 1*C*). Blue light-induced unfolding of the Jα helix unleashes the linked effector to restore its native function. To expand the caging interfaces and extend optical control over proteins requiring free NH_2_ termini, a set of LOV2 circular permutants (cpLOV2) have recently been developed by connecting the original NH_2_/COOH termini with a flexible linker and creating new termini at positions immediately before the Jα helix (23). cpLOV2 adopts an overall structure reminiscent of LOV2 and maintains superior photosensitivity. cpLOV2 is an important complement to LOV2 because it opens more engineering opportunities by allowing the fusion of an effector domain to the NH_2_-terminal end of the Jα helix or between the reordered Jα helix and PAS core (23).

Aside from functioning as a convenient caging tag, LOV2 or cpLOV2 can be modularly inserted into POIs as an allosteric switch to control the host protein activity. LOV2- or cpLOV2-based optogenetic approaches have been successfully employed to control the functions of short peptides such as nuclear localization (NLS) or nuclear export (NES) signal (117–120), as well as proteins of moderate or considerable sizes, including ion channels (22, 23, 121–123), DNA-binding proteins (124), enzymatic or signaling proteins (23, 116, 125–127), cytoskeletal motors (128), and anti-CRISPR proteins (23, 129).

#### 3.1.3. YtvA-LOV.

*Bacillus subtilis* YtvA comprises an NH_2_-terminal photosensory LOV domain and a COOH-terminal STAS domain that binds nucleotide triphosphates (130, 131). The NH_2_-terminal region (residues 1–126) contains a LOV core domain (cLOV, residues 25–126), an NH_2_-terminal A′α helix (residues 1–25), and a Jα linker region (residues 127–147) that joins cLOV with STAS (132, 133). Unlike the COOH-terminal Jα of AsLOV2 that docks tightly toward the PAS core, the Jα linker region in YtvA does not appear to pack against the cLOV domain (133). When purified as a recombinant protein in vitro, YtvA-LOV (residues 20–147) was found to dimerize in a head-to-head fashion even in the dark. Upon photostimulation, the two subunits of one YtvA-LOV dimer rotate relative to each other by 5° (133). This moderate structural change suggests that YtvA-LOV employs a light-dependent signaling mechanism distinct from that postulated for plant-derived LOV domains.

### 3.2. Protein Self-Oligomerization

Ligand-induced protein homooligomerization is another widespread mechanism employed by mammalian cells to initiate or terminate cell signaling, as exemplified by fibroblast growth factors (FGFs) binding to their receptors (FGFRs) to trigger downstream cytoplasmic signaling cascades (134, 135). The use of light to control protein oligomerization obviates the need of natural ligands or stimuli, allowing optical interrogation of many oligomerization-dependent cellular events in a reversible manner (FIGURE 2*B*).

#### 3.2.1. CRY2, CRY2olig, and CRY2clust.

*Arabidopsis* CRY2 undergoes monomer-to-oligomer transition upon blue light illumination through conformational changes in the NH_2_-terminal PHR domain (residues 1–498; FIGURE 1*E*) within seconds (59, 60, 62, 68). A light-insensitive mutant, D387A, abolishes the clustering ability of CRY2. Further studies have been conducted to optimize the speed and the oligomeric state of CRY2 by screening over PHR variants and fusion with additional peptides. An E490G mutant (CRY2olig) was found to significantly enhance the light-mediated oligomerization (136). Since the COOH-terminal charges could impact CRY2 clustering, CRY2high (E490R) and CRY2low (residues 1–488 + EED extension) variants were further engineered to tune the extent of oligomerization (66). In addition, a 7-mer peptide (ARDPPDL) extension at the COOH terminus of PHR (CRY2clust) is reported to promote its self-clustering (67). Likewise, the fusion of intrinsically disordered regions (IDRs) to the COOH terminus of PHR leads to the generation of optoDroplets, which exhibit light-inducible aggregation or phase transition in living cells (137). Because the relative positioning and fusion of various fluorescent protein (FP) tags may affect the efficiency of CRY2 clustering (67), parallel testing on multiple constructs is strongly recommended to identify an ideal FP-tagged CRY2-fusion protein.

#### 3.2.2. AuLOV.

*Vaucheria frigida* aureochrome 1 (VfAU1) contains a bZIP DNA-binding domain and a LOV domain that work together to mediate light-dependent association of vfAU1 with the genomic DNA sequence TGACGT. Unlike the plant LOV2, the Jα helix in vfAU1-LOV is partially folded back on the outer surface of the β-sheet and lacks a Glu/Lys salt bridge found in all other major LOV domains (48). The vfAU1 bZIP-LOV fragment exists predominantly as a dimer because of the intrinsic dimerization of bZIP. By contrast, the vfAU1-LOV domain alone occurs largely as a monomer at a low concentration in the dark. Blue light irradiation at ∼450 nm enhances intermolecular interactions between two LOV subunits to shift the equilibrium toward a dimeric form.

A similar dimeric bZIP-LOV domain is found in *Phaeodactylum tricornutum* aureochrome 1a (PtAU1a). The interaction between LOV and bZIP in the dark impedes PtAU1a-DNA binding. Blue light stimulation triggers intramolecular bZIP–LOV dissociation, followed by LOV dimerization to enhance the DNA-binding affinity of PtAu1a. Unlike the dimeric full-length paAU1a or the bZIP-LOV domain, PtAu1a-LOV alone remains monomeric even at a concentration as high as ∼4 mM in the dark but quickly assembles as a dimer after blue light illumination. Monomer-to-dimer transition is driven by the light-induced unfolding of the NH_2_-terminal A′α and Jα helices that uncovers the dimerization interface of LOV (138, 139).

#### 3.2.3. CPH1S.

The NH_2_-terminal photosensory domain of *Cyanobacteria* phytochrome Cph1, designated Cph1S, undergoes light-dependent changes in its oligomeric state. Cph1S stays at the homodimeric Pfr state under red light peaked at 660 nm and adopts a monomeric Pr state in the presence of far-red light (740 nm) or when kept in the dark (109). Codon-optimized Cph1S (Cph1S-o) was fused to the intracellular domain of TrkB and FGFR1 to enable red light-inducible activation of RTK-mediated signaling, a process that could be spontaneously reversed within 30 min in the dark (109).

### 3.3. Protein-Target Heterodimerization

Ligand-induced signal transduction can also be transmitted via delocalization of a POI from its original place to a new destination through protein-protein, protein-DNA, or protein-lipid interactions. A variety of optical dimerizers have been invented to achieve the same functional consequences with light (FIGURE 2*C*).

#### 3.3.1. UVR8-COP1.

Upon UV light-induced dissociation of UVR8 dimer, monomeric UVR8 in its activated state interacts with COP1 (140), with the minimal interacting region mapped to a COOH-terminal 340-residue fragment. This was confirmed in cellulo by monitoring UV-B light-induced recruitment of mcherry-NLS-COP1 to chromatin marked by histone H2B-GFP-UVR8, as well as nuclear translocation to induce light-dependent luciferase gene expression (27).

#### 3.3.2. CRY2-CIB1.

The CRY2-CIB1 pair is one of the most popular optical dimerizers applied to control protein-protein interactions (63, 141, 142). In addition to mediating photoinducible homooligomerization as discussed above, the FAD-bound NH_2_-terminal photolyase homology region of CRY2 shows blue light-dependent heterotypic interaction with CIB1. The minimal CRY2-interaction region in CIB1 was mapped to the NH_2_-terminal 170 residues of CIB1 (CIBN). Although shorter CIBN fragments (1–81 or 16–43) exhibit a similar light-dependent association with CRY2, their dynamic ranges are substantially reduced (68). To reduce dark activation and diversify photoactivation kinetics, the second generation of CRY2-CIB-based dimerizers were further optimized, including a longer CRY2 truncation (residues 1–535) that showed stronger heterodimerization with CIBN and the CRY2 mutants L348F or W349R with prolonged or shortened photocycle (*t*_1/2,off_ ∼24 min or ∼2.5 min), respectively (65). The CRY2-CIB1 pair was applied to reconstitute the enzymatic activity of a photoactivatable Cre recombinase (PA-Cre) for genome engineering with a single pulse of light (65). This optical dimerizer was also coupled with TALE (transcription activator-like effector)- or CRISPR (clustered regularly interspaced short palindromic repeats)-based transcriptional reprogramming systems to allow spatiotemporal control of endogenous gene expression (143–146).

#### 3.3.3. TULIP (LOVpep-ePDZ).

The LOV2-based optical dimerizer TULIP consists of an engineered PDZ domain (ePDZ) and its interacting peptide fused to the COOH terminus of LOV2-Jα helix (LOVpep) (147). In the presence of blue light, the peptide is fully exposed and physically interacts with ePDZ. When photostimulation ceases, the Jα-peptide region adopts a well-folded structure and docks against the LOV2 core to disrupt ePDZ binding. Because the dissociation constant (*K*_d_) for the PDZ-peptide interaction can be tuned via mutagenesis in the range of 0.5 nM to 10 µM (148), a set of TULIP variants with varying affinities and kinetics were engineered to control protein-target heterodimerization. The TULIP system was first applied to manipulate MAPK signaling and cell polarity via light-inducible translocation of Ste5 and Ste11 to the PM in yeast (147). One potential caveat associated with TULIP is that the PDZ domain and its binding peptide might cause undesired cross talk with endogenous PDZ-dependent signaling pathways.

#### 3.3.4. iLID (SspB + LOV2-ssrA).

Similar to TULIP, the improved light-induced dimer (iLID) tool comprises two components: the *Escherichia coli* SsrA peptide fused to the COOH-terminal Jα helix of LOV2 (LOV2-ssrA) and its natural binding partner SspB (149–151). iLID was evolved from its prototype (152) by introduction of several key mutations, including R73Q in SspB and G528A/N528E in LOV2 that significantly improved the affinity for SspB and reduced the dark activity (153). The iLID-nano variant exhibited ∼40-fold change in binding affinity, with the dissociation constants determined to be 132 nM at 470 nm and 4.7 µM in the dark state. Weaker versions of iLID, named iLID-micro and iLID-milli (154), were also designed to better control proteins at high effective concentrations (5–100 µM) with diminished dark activity. Both components of iLID seem to exist as monomers in solution, which could avoid futile interactions by eliminating the competition between the self-oligomerization of one component (such as CRY2) and heterodimerization as seen in the CRY2-CIB1 pair. Unlike TULIP that is built upon mammalian PDZ and its interacting peptide, iLID is solely engineered from bacterial proteins and therefore has minimal cross talk with host cell signaling.

#### 3.3.5. VVD and its variants (Magnets and eMags).

VVD, a LOV-containing photoreceptor derived from *Neurospora crassa*, rapidly forms a homodimer in response to blue light but returns to the monomeric state with a rather slow switch-off kinetics (*t*_1/2,off_ 2.8 h) (46, 155, 156). VVD-LOV was also engineered into an optical heterodimerization system (designated Magnets) made of pMag (positive Magnet, I52R/M55R mutant) and nMag (negative Magnet, I52D/M55G mutant) that underwent light-induced electrostatic interactions (157). After further incorporation of the I85V substitution or I74V/I85V double mutations into Magnets, the deactivation kinetics was shortened from 1.8 h to 4.2 min or 25 s, but the dimerization extent was sacrificed by nearly 50%. To avoid this unwanted trade-off, M135I/M165I substitutions were subsequently introduced to prolong the dissociation half-life (*t*_1/2,off_ 4.7 h) and enhance the extent of dimerization by 17-fold. The application of Magnets is somewhat limited by two drawbacks: the requirement of pMag/nMag concatemers for efficient heterodimerization and preincubation of engineered targets at nonphysiological temperature (28°C) for maximal functionality. Very recently, an enhanced Magnets (eMags) tool was invented through structure-guided protein engineering (158). eMags exhibited enhanced dimerization efficiency and thermal stability, as well as faster kinetics (τ_on_ = 2.8–3.6 s; τ_off_ = 14.0–23.1 s). The eMags tool was applied to reversibly induce interorganelle contacts to reprogram phospholipid metabolism (158). Worthy of mention, because both components in Magnets or eMags require light stimulation to be activated, its background activity in the dark and the spatial controllability seem to be superior over the iLID or CRY2-CIB1 systems (159).

#### 3.3.6. OptoPB-lipid and BcLOV4-lipid.

To meet the need for real-time control over protein-lipid interactions in living cells, OptoPB was developed by fusing LOV2 with phospholipid-binding polybasic (PB) domains derived from stromal interaction molecules (STIMs) or small GTPases (160, 161). The endoplasmic reticulum (ER)-anchored version of OptoPB [termed OptoPBer (160) or LiMETER (for light-inducible membrane-tethered peripheral ER)] further enabled light-inducible ER-PM junction formation (162), with intermembrane space subjected to phototuning at nanoscale. OptoPBer has been used to demonstrate that an optimal gap distance between ER and PM (>10–15 nm) is required for the diffusion of ORAI1 Ca^2+^ channels into ER-PM junctions to mediate effective Ca^2+^ influx (160, 163).

Whereas OptoPB is an artificial synthetic photoswitchable lipid binder, BcLOV4, a LOV domain-containing photoreceptor from *Botrytis cinerea*, is naturally evolved to interact with the anionic phospholipids embedded in the PM inner leaflet through electrostatic interactions in a light-dependent manner. In the dark state, an NH_2_-terminal regulator of G protein signaling (RGS) domain seems to inhibit the electrostatic interaction to prevent lipid binding and PM translocation (164, 165). After blue light stimulation, the unmasking of a membrane lipid-interacting polybasic amphipathic helical linker between the LOV Jα-helix and a COOH-terminal domain of unidentified function is speculated to mediate the BcLOV4-lipid interaction (165).

#### 3.3.7. EL222-DNA.

EL222, a 222-residue transcription factor from the marine bacterium *Erythrobacter litoralis*, contains an NH_2_-terminal LOV domain and a helix-turn-helix (HTH) DNA binding domain (166). Blue light illumination disrupts the intramolecular LOV-HTH association, allowing EL222 to dimerize and associate with its cognate DNA sequences (166, 167). When replacing the native helix-turn-helix domain with a VP16 transcriptional activation domain, a VP-EL222 chimeric construct allows light-inducible synthetic gene expression driven by five copies of the EL222-binding C120 (Clone 1–20 bp) sequence and a downstream TATA box promoter (47).

#### 3.3.8. FKF1-GIGANTEA.

In *Arabidopsis*, flavin-binding Kelch repeat F-box1 (FKF1) forms a complex with GIGANTEA in a blue light-dependent manner to promote daytime *Constans* gene expression and regulate photoperiodic flowering (168). When heterologously expressed in mammalian cells, the FKF1-GIGANTEA pair was initially used for Rac targeting to the PM to induce lamellipodia formation (49). This optical dimerizer was later used to promote Ca_V_1.2 channel interactions for the amplification of Ca^2+^ influx and signaling (169). Along with VP16 and engineered zinc finger proteins or the DNA binding domain of Gal4, FKF1-GIGANTEA also enables light-inducible spatiotemporal control of transcriptional activation (49, 170, 171). Although it is useful for eliciting long-lasting effects in light-illuminated targets, the extremely slow reversal kinetics (*t*_1/2,off_ = 62.5 h) of the FKF1-GIGANTEA tool (172) greatly impedes its application in reversible and dynamic control of cell physiology.

#### 3.3.9. PhyA-FHY1/FHL.

Far-red light photoreceptor PhyA from *Arabidopsis thaliana* transduces signaling through its binding partner, far-red elongated hypocotyl 1 (FHY1), or its homology FHY1-like (FHL) for nuclear translocation and transcriptional regulation. Hence, PhyA-FHY1 and PhyA-FHL were also repurposed as red light-induced protein-protein heterodimerization pairs. The PhyA-FHY1/FHL nucleocytoplasmic shuttling is regulated by red/far-red light in the plant, with FHY1 containing a nuclear localization signal to directly mediate nuclear translocation. With red light, the nuclear entry of PhyA is inhibited because of the phosphorylation of FHY1. Subsequent exposure to far-red light reverses FHY1 phosphorylation to cause PhyA translocation into the nucleus. The transcription of FHY1 and FHL is further controlled by transcription factors such as far-red elongated Hypocotyl 3 and far-red impaired response 1 (173, 174). The PhyA-FHL pair was fused to GAL4 and GAL1, respectively, to develop a red light-inducible gene expression system in yeast and mammals (175, 176).

#### 3.3.10. PhyB-PIF.

PhyB absorbs red light (650 nm) to switch from its inactive form Pr to an active Pfr state, enabling its heterotypic interaction with PIFs with subsequent nuclear entry to modulate gene expression (103, 177). PhyB readily reverses back to its Pr inactive state under infrared light at 750 nm. Structural studies on the NH_2_-terminal PAS-GAF-PHY module reveal a knot-lasso structure formed by a loop extending between strand β4 and helix α4 of the GAF domain, which lassos the conserved isoleucine (Ile35) of the polypeptide chain upstream of PAS to generate a tight water-excluded interface and direct the sequence upstream of PAS toward the bilin cofactor (177) (FIGURE 1*G*). A series of PhyB truncation variants were designed to pair with PIF3 or PIF6 to enable red/infrared light-controllable protein-protein heterodimerization (103). The PhyB-PIF pair shows an activation half-life of 1.3 s and a deactivation half-life of 4 s based on its performance in a cytosol-to plasma membrane recruitment assay. As mentioned above, the PhyB-PIF system requires the addition of exogenous toxic PCB as the indispensable cofactor for photoreaction.

#### 3.3.11. Am1-BAm.

*Acaryochloris marina* Am1_c0023g2 (hereafter abbreviated as Am1), a red/green GAF domain from the cyanobacteriochrome photoreceptor family, binds PCB or BV as the light absorber. By loading with either PCB or BV chromophores, Am1 can be made responsive to a wide range of light inputs, ranging from orange to far-red light. When bound with PCB, red light irradiation (680 nm) converts Am1 from a red light-absorbing Pr state to a green light-absorbing Pg state. Green light (525 nm) reverses Am1-PCB back to the Pr state. By contrast, BV-bound Aim 1 undergoes reversible Pfr-to-Po conversion in the presence of orange (595 nm) or far-red (750 nm) light. Engineered monomeric Am1 bearing the L159K mutation and a truncated NH_2_-terminal helix was coupled with its natural binding partner BAm to form a versatile optical dimerizer that responds to green, orange, red, or far-red light (178).

#### 3.3.12. BphP1-PpsR2/QPAS1.

Bacterial BphP1 uses biliverdin chromophore and forms a heterodimer with a transcription repressor PpsR2 upon NIR light stimulation (102). The BphP1-PpsR2 interaction reverses upon 640-nm illumination or spontaneously in the dark within 15 min. To minimize the overall size of the tool and avoid the unwanted oligomeric behavior of PpsR2 (179), a truncated form of PpsR2 (residues 101–251), Q-PAS1, was generated for more efficient transcriptional regulation by NIR light (180).

#### 3.3.13. NanoReD (DrBphP-LDB).

A nanobody-based, red light-induced dimerization system (nanoReD) was created by screening nanobody-based binders (LDBs) for the minimal photoactive module (PAS-GAF-PHY; 60 kDa) truncated from *Deinococcus radiodurans* phytochrome (DrBphP) in the presence of cofactor biliverdin (181). Two best-performing candidates, LDB-3 and LDB-14, displayed far-red light-inducible interaction with DrBphP at 654 nm, with the apparent dissociation constants estimated to be 0.5–1 μM by isothermal calorimetry titration or 2.4–7.7 μM by biolayer interferometry (BLI) measurements. In the dark form, the BLI-determined apparent *K*_d_ fell in the range of >10–25 μM. Given its compatibility with the tissue transparency window (650–900 nm), the nanoReD system is likely to enable more convenient deep-tissue optogenetic applications without the need for supplying exogenous photosensing cofactors.

### 3.4. Protein-Protein Dissociable Systems

#### 3.4.1. UVR8.

UVR8 primarily stays as a homodimer in its ground state but rapidly dissociates into monomer upon UV-B irradiation at 300 nm (FIGURE 2*D*). This property was utilized to generate a light-inducible protein secretion system in which a UVR8 fusion protein was initially sequestered in the endoplasmic reticulum and then switched to the secretory pathway for plasma membrane trafficking after UV light stimulation (182). UVR8 was also used to generate a hydrogel nanofiber network capable of photoresponsive gel-solvent phase transition (183). This synthetic system contains a mixture of UVR8-fused hexapeptide (WRESAI) and its binding partner tax-interacting protein-1 (TIP1). In the dark, the UVR8-peptide/TIP1 complex forms a hydrogel network but quickly becomes a clear solution upon UV-B irradiation. Such a light-responsive hydrogel device will likely find translational applications in controllable drug delivery and smart biomaterial design.

#### 3.4.2. DronpaN and pdDronpa1.

Dronpa is a fluorescent protein with its fluorescence switched on and off at violet (400 nm) and cyan light (500 nm), respectively. A tetrameric fluorescent Dronpa mutant K145N (abbreviated as DronpaN) was shown to exhibit tetramer-to-monomer transition at 500 nm (87). Illumination at 400 nm can reverse the process to induce retetramerization. This feature was utilized to design light-controllable enzymes. For instance, a DronpaN-protease-DronpaN fusion was used to block the hepatitis C virus nonstructural 3 (NS3) protease activity in the dark. The target protein mCherry was anchored to the plasma membrane through a CAAX-box farnesylation signal peptide, with an NS protease cleavage site inserted in between. Blue light stimulation uncaged the NS3 protease and restored its enzymatic activity to cleave the peptide bond, with subsequent release of mCherry from PM to the cytosol (87).

For easier control of protein activity via dimer-to-monomer interconversion, a photodissociable dimeric Dronpa 1 (pdDronpa1) was further engineered by introducing mutations into Dronpa145N. This was made possible through the rational introduction of mutations to break the antiparallel dimer interface and strengthening of the cross-dimer interface in DronpaN (88). Two pdDronpa1 molecules can be conveniently attached to both ends of a POI so that their dimerization at 400 nm masks the active site of a POI, but 500-nm light-induced monomerization removes the steric hindrance to restore protein function. Violet light not only causes reactivation of green fluorescence but also allows the redimerization of pdDronpa with a half-life of ∼30 min. This strategy has been applied to control the activity of a series of kinases and small GTPases (87, 88).

#### 3.4.3. LOVTRAP (LOV2-Zdk).

LOV2 trap and release of protein (LOVTRAP) was initially engineered to anchor and release POIs from subcellular locations by using LOV2 and its synthetic binder, Zdark (Zdk) (184). Zdk proteins (Zdk1–3) are evolved from the Z domain of staphylococcal protein A through mRNA display-based screening, which preferentially recognizes LOV2 in its dark state but not in the lit state. Structural analysis of the Zdk1-LOV2 complex in the dark revealed that the LOV2 Jα helix tightly docked against two helices of Zdk1. The binding affinities of Zdks toward the dark and lit states of LOV2 differ by >15-fold [*K*_d_: 11.4–26.2 nM (dark) vs. 0.5–4 µM (lit)] (184). Photoinducible Zdk-LOV2 dissociation occurs in <1 s, whereas the association half-life can be adjusted between 2 s and 500 s by taking advantage of fast- or slow-cycling LOV2 mutants (38–41).

#### 3.4.4. PixD-PixE.

PixD (Slr1694) and PixE (Slr1693) are found in *Synechocystis sp. PCC6803* to mediate cyanobacteria phototaxis. PixE drives the aggregation of PixD dimers to form large multisubunit complexes in the dark with a PixD_10_:PixE_5_ stoichiometry. Upon blue light stimulation, structural changes within PixD destabilize the PixD-PixE complex and cause the dissociation of the complex into PixD dimers and PixE monomers within seconds (53, 185). The engineered PixD-PixE pair has been repurposed for optical control of liquid-liquid phase separation in mammalian cells (186) and metabolic flux in yeast (187).

#### 3.4.5. RsLOV.

LOV domain from *Rhodobacter sphaeroides* (RsLOV) contains an NH_2_-terminal A′α helix and a unique long COOH-terminal HTH motif extension (Jα and Kα) that is similar to EL222 but dimerizes in the dark. Dimeric RsLOV dissociates into monomer upon blue light stimulation (188). Capitalizing on this property, Cas9-RsLOV was engineered to block the Cas9 catalytic activity in the dark. Under blue light irradiation, the function of Cas9 was restored upon RsLOV monomerization (189). Therefore, RsLOV can be used to confer photosensitivity on an unrelated nonnatural POI for optogenetic engineering.

#### 3.4.6. MxCBD and TtCBD.

MxCBD or TtCBD within the bacterial photoreceptor CarH serve as transcriptional repressors of carotenoid genes and mediate CarH tetramerization in the dark, allowing CarH to bind to carotenoid promoters through its HTH-DNA binding motif. Upon absorption of green light at 550 nm, photolytic cleavage of the 5′‐deoxyadenosyl group of the 5′‐deoxyadenosylcobalamin cofactor leads to CBD monomerization and DNA dissociation. The release of CarH from DNA leads to gene activation. TtCBD was also fused with a transactivator to enable green light-inducible transgene expression, such as the human glucagon-like peptide-1 for the treatment of mice with experimental type 2 diabetes (190). MxCBD and TtCBD have been used to control FGFR1 activity by fusing to the COOH-terminal intracellular domain of FGFR1 and replacing the NH_2_-terminal ligand-binding and transmembrane domains with a myristoylation anchor for PM tethering (93). Activation of FGFR1-MxCBD/TtCBD was observed, as reflected by ERK phosphorylation in the dark. At 540 nm, the reversal of FGFR1 activation was only observed in the construct containing MxCBD but not TtCBD. It turned out that the corrin ring of the cofactor of TtCBD forms a stable adduct with a histidine residue, thereby inhibiting the release of the cofactor from its binding pocket to make the photoreaction irreversible (93). Such discrepancy between MxCBD and TtCBD provides engineering possibilities for reversible or irreversible control of protein-protein dissociation by light.

#### 3.4.7. DrBphP.

DrBphP consists of a core NH_2_-terminal PSM domain and a COOH-terminal histidine-kinase domain connected by an α-helix. The DrBphP-PSM undergoes reversible monomer-to-dimerization when the light switch is toggled between red (660 nm, Pr state) and infrared (780 nm; Prf state). DrBphP was used to develop optokinases (Dr-TrkA and Dr-TrkB) for NIR light control of RTK signaling (191). Given its unique far-red/NIR-shifted optical activation window, DrBhpP allows multiplexing with other visible light (400–600 nm)-responsive optogenetic devices.

### 3.5. Photocleavable Proteins

The photocleavable protein (PhoCl) was generated based on a circularly permutated fluorescent protein, mMaple, that spontaneously splits into two fragments after violet light (400 nm)-induced cleavage of the polypeptide backbone. Photostimulation leads to the generation of a short NH_2_-terminal fragment (66 residues) and a 166-residue COOH-terminal fragment (FIGURE 2*E*), with a dissociation rate of ∼500 s (192). PhoCl2c with improved dissociation extent and PhoCl2f with a faster dissociation rate (*t*_1/2_ < 30 s) were further developed to enable robust optogenetic control of protein localization and protein-protein interactions (193). PhoCl-based optogenetic tools have been applied to control enzymes (Cre recombinase and protease), ion channel (pannexin-1), and the Gal4 transcription factor (192).

## 4. APPLICATION OF OPTOGENETICS IN PHYSIOLOGICAL PROCESSES

Optogenetics provides opportunities to interrogate important physiological processes. At the molecular and cellular levels, optogenetic tools have been applied to remotely control ion channel actuation, membrane receptor-mediated signaling, cytoskeleton remodeling, organelle positioning, phase separation, and protein degradation and cleavage. At the systematic level, successful examples of optogenetic applications include cell-to-cell communication, immunomodulation, transcriptional reprogramming, cell death, cellular metabolism, and Cre/LoxP-mediated genome engineering. Rather than exhaustively enumerate the growing list of optogenetic applications, we highlight representative examples to showcase the versatility and broad applicability of optogenetics in precise interrogation of cell physiology (TABLE 2).

**Table 2.** Selected applications and attributes of representative PSMs

Optogenetic System	PSM	Attributes	Selected Applications
UVR	UVR8	Phototoxicity from UV light	Protein secretion (182); light-responsive hydrogel network (183)
UVR	UVR8/COP1	Irreversible, large size, slow kinetics, phototoxicity from UV light	Nuclear translocation (27)
LOV	LOV2 or cpLOV2	Limited choice of available caging surfaces; efforts required to test linker length and minimize dark activity. cpLOV2 provides additional opportunities for effector caging and allosteric control.	Ion channel (22, 23, 121–123); anti-CRISPR proteins (23, 129); GTPase signaling: PA-Rac1, PI-Rac1, PI-Src (116, 126); subcellular organelle targeting (267); molecular recorder: FLARE (315) and FLiCRE (314); necroptosis and pyroptosis (23, 24); intrabody-antigen recognition (339–341)
LOVTRAP	LOV2 or cpLOV2/Zdk1	Dark-state binding; limited choice of available caging surface	Cytoskeleton dynamics (246, 247)
LOV-HTH	RsLOV	Similar to EL222 but dimerizes in the dark and monomerizes under blue light	Cas9 activity (189)
TULIP	LOVpep/ePDZ	PDZ domain and its binding peptide might cause undesired cross talk with endogenous PDZ-dependent signaling pathways.	Organelle positioning (264)
iLID/cpLID	LOV2-ssrA or ssrA-cpLOV2/SspB	Unlike TULIP, iLID is engineered from bacterial proteins and has minimal cross talk with endogenous cell signaling; some background dark-state binding if overexpressed	Cytoskeleton dynamics (254) and SxIP-iLID (247); ion channel: OptoRGK (230); interorganelle contacts (273); CAR-T cell therapy (23, 303); phase separation and transcriptional regulation: CasDrop (279), Corelet (280); mitophagy (284)
LOV-HTH	EL222/DNA	Single DNA recognition site; limited transferability to other genomic targets	DNA binding (166, 167); transcriptional regulation (47); autophagy regulation (281)
LOV-FKF1	FKF1/GIGANTEA	Large size, slow kinetics, sensitive to expression levels	Ca^2+^ signaling (169); transcriptional regulation (49, 170, 171)
LOV-PB	OptoPB/Lipid	OptoPBer and LiMETER	Protein-phospholipid interaction; reversible assembly of membrane contact sites at ER-PM junctions (160, 162)
RGS-LOV	BcLOV4/Lipid	Naturally evolved to interact with phospholipids in the PM inner leaflet	Lipid binding for protein translocation (165)
bZIP-LOV	VfAU1-LOV or ptAU1a-LOV	Intramolecular interaction between bZIP and LOV in the dark; light-induced bZIP-LOV dissociation followed by AU1-LOV dimerization	RTK signaling: Opto-RTK (FGFR1, EGFR, RET, TrkA/B) (139, 231, 232)
Ncap-LOV	VVD+/VVD-pMag/nMag	Requirement of pMag/nMag concatemers for efficient heterodimerization and preincubation of engineered targets at nonphysiological temperature (28°C) for maximal functionality	Interorganelle contacts (158); Cre recombinase activity (293); intrabody-antigen recognition (342); Cas9 activity (350)
BLUF	PixD/PixE	PixE drives oligomerization of PixD dimers in the dark (PixD_10_:PixE_5_). Light-induced PixD/PixE dissociation into PixD dimers and PixE monomers.	Phase separation: PixELL (186); metabolic flux (187)
BLUF	PAC	BLUF domain linked to an adenylyl cyclase domain	Ion channel: PAC-K (195); bPAC for cAMP production (241)
Cryptochrome, CRY2clust CRY2 (E490G)	CRY2, CRY2clust, CRY2olig	High background using CRY2 for protein oligomerization	Ion channel (220, 223); phase separation: optoDroplets (137); innate immune signaling: OptoMyD88, OptoTRAF6, Photo-SMOCs (310, 311); necroptosis: LiPOP1 (24); protein degradation: OptoTrim-Away (349)
Cryptochrome	CRY2/CIB1	Potential background of CRY2 oligomerization; competition between CRY2 self-oligomerization and heterotypic interaction with CIB1	Cre recombinase (65, 292); transcriptional reprogramming (143–146); phase separation and transcriptional regulation: OptoTF (278); innate immune signaling (312); apoptosis: OptoBax (319, 360); protein degradation: LiPD (344); RNA modifications: PAMEC (356)
Fluorescent protein	PYP	Requirement of exogenous supply of *p*-coumaric acid	GCN4Δ25PYP/DNA binding (114, 115)
Rhodopsin	ChR2	Non-ion selective, not suitable for nonexcitable cells; desensitization	T cell trafficking: PA-CXCR4 (301)
Fluorescent protein	Dronpa145N, pdDronpa1	Tetramer-to-monomer transition at 500 nm and can be reversed by 400-nm light	Enzymatic activity (87); transcriptional regulation (353)
Cobalamin-binding domain	MxCBD, TtCBD	Differential regulation of protein-protein dissociation by light in between MxCBD (reversible) or TtCBD (irreversible)	Transcriptional regulation (190); receptor signaling (93)
Cyanobacteriochrome	Am1/BAmgreen or BAmred	Multicolor optogenetics	Transcriptional regulation (178)
Phytochrome	CPH1	Deep tissue red light-inducible RTK signaling activation	RTK signaling (109)
Phytochrome	PhyA/FHY1 or FHL	Requiring exogenous supply of chromophore phycocyanobilin (PCB)	REDMAP for MAPK signaling, epigenetic remodeling, and gene expression (176)
Phytochrome	PhyB/PIF	Requiring exogenous supply of chromophore phycocyanobilin (PCB); large size; sensitive to expression	Cell-cell interactions (359); GTPase signaling: Opto-SOS (234); phospholipid metabolism (108); opto-ligand T-cell receptor system (299)
Phytochrome	BphP1/PpsR2 or Q-PAS1	Large size; dimerization of individual elements	Transcriptional regulation (102, 180)
Phytochrome	DrBphP1	Unique far-red/NIR-shifted photosensitivity allows multiplexing with other visible light modules	Opto-kinases and RTK signaling (191)
Phytochrome	DrBphP/LDB	Deep-tissue optogenetic applications without the need of supplying exogenous photo-sensing cofactors; Nanobody-based dimerization system	NanoReD for protein translocation and transcriptional regulation (181)
Phytochrome	LAPD	LAPD can be used in parallel with bPAC to enable cAMP degradation with red light and cAMP biogenesis with blue light	cAMP and cGMP hydrolysis (243)
Photocleavable protein	PhoCl	Violet light (400 nm)-induced PhoCl cleavage enables protein-protein interaction and dissociation; irreversible and intense photostimulation.	Enzymatic activities, ion channel and transcriptional regulation (192)

See glossary for abbreviations.

### 4.1. Ion Channels and Receptor-Mediated Signaling

#### 4.1.1. Ion channels.

**FIGURE 3.**
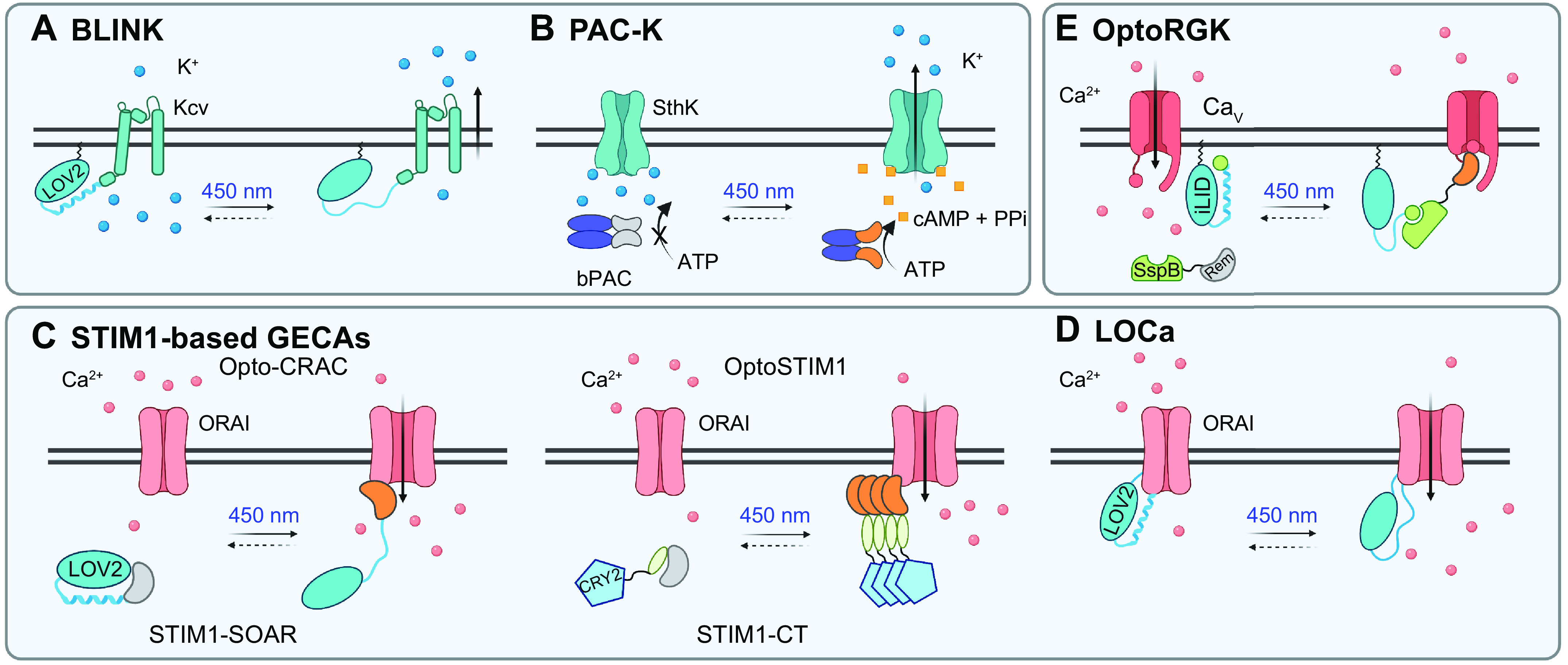
Optogenetic engineering of K^+^- or Ca^2+^-selective channels. *A* and *B*: design of blue light-activatable K^+^ channels. BLINK is engineered by fusing LOV2 domain to a viral K_cv_ channel (*A*), and PAC-K consists of a small cyclin nucleotide-gated SthK channel and a light-activatable bPAC (*B*). *C* and *D*: engineering of CRAC channels to enable optical control of Ca^2+^ influx in mammalian cells. *C*: STIM1-based GECAs including LOV2-STIM1 (Opto-CRAC) and CRY2-STIM1 (OptoSTIM1) hybrid constructs that gate PM-resident ORAI channels to induce Ca^2+^ influx upon blue light stimulation. *D*: design of light-operated Ca^2+^ channel (LOCa) by installing LOV2 domain into the intracellular loop of an engineered ORAI1 Ca^2+^ channel. *E*: blue light-mediated inhibition of Ca_V_ channels via conditional recruitment of an engineered Rem GTPase toward the PM using the iLID-sspB optical dimerizer. See glossary for abbreviations. Image created with BioRender.com and used with permission.

Optogenetic engineering of nonopsin channels has thus far achieved great successes with two classes of targets, the potassium channel and the calcium release-activated calcium (CRAC) channel. A viral potassium channel, K_cv_, has been engineered to drive cell hyperpolarization via light-inducible K^+^ flux and terminate the excitatory currents without external cofactors or extracellular stimuli (122). The synthetic channel, named BLINK1 (blue light-induced potassium channel 1), was designed by fusing the K_cv_ channel from *Paramecium bursaria* Chlorella virus 1 to the COOH terminus of a PM-tethered LOV2 domain (FIGURE 3*A*). BLINK1 was born out of a functional screen by using light-induced growth rescue of the Δtrk1 Δtrk2 potassium transport-deficient yeast, followed by random mutagenesis to enhance the light responsiveness and channel activation. This engineered Kcv-LOV2 hybrid channel has several key modifications to both elements, including the N538A mutation in LOV2 to increase LOV2-K_cv_ interaction, P13L to affect channel gating, A7T in the myristoylation/palmitoylation sequence, and a LOV2-Jα helix lacking the last nine residues. BLINK1 has demonstrated high K^+^ selectivity and relatively slow kinetics (τ_on_ 87 s and τ_off_ 168 s), making it suitable for long-term control of channel activity. A proof-of-concept in vivo study was performed in zebrafish to manipulate embryonic escape motion by blue light. Further improvement of its initial variant (BLINK2) was made by including the COOH terminus of inward-rectifier K^+^ channel subunit 1 (KAT1) with a binding motif ^673^YFSDN^677^ derived from the 14-3-3 protein to promote expression efficiency and render the light-gated potassium channel more compatible with mammalian neurons (194). Activation of BLINK2 with a brief blue light pulse could lead to a reduction in pain sensation for >30 min in a rat model of neuropathic pain.

In a second approach, a light-gated potassium channel (designated PAC-K; FIGURE 3*B*) for optogenetic silencing was engineered on the basis of a synthetic two-component system, consisting of the small cyclin nucleotide-gated potassium channel SthK and photoactivatable BLUF-containing adenylyl cyclases (PACs) (195). Blue light was applied to induce PAC-catalyzed cAMP production, which subsequently gates the SthK channel to silence excitable cells. A single pulse of light was able to robustly suppress electrically evoked action potentials for almost 5 min in cardiomyocytes and up to 1 min in cultured hippocampal neurons.

Compared with excitable tissues that rely on Na^+^ or K^+^ to alter the membrane potential and initiate signaling, nonexcitable cells remain largely inert to electric signals but are adept at using Ca^2+^ as a versatile secondary messenger to relay signals. Genetically encoded Ca^2+^ channel actuators (GECAs; FIGURE 3*C*) built upon GPCRs and the Ca^2+^ release-activated Ca^2+^ (CRAC) channel have been widely used to remotely control intracellular Ca^2+^ signaling (25, 121, 196, 197). Microbial opsin-based tools used in excitable cells have fast kinetics (ms) but lack ion selectivity, hence impeding their applications in nonexcitable cells. Light-induced activation of synthetic or vertebrate GPCRs (opto-XR and melanopsin) (82, 198, 199) are capable of eliciting inositol trisphosphate (IP_3_)-dependent intracellular Ca^2+^ mobilization, but at the cost of coactivating other pathways modulated by another secondary messenger, diacylglycerol (DAG).

To overcome these limitations, highly Ca^2+^-selective optogenetic tools engineered from the two-component CRAC channel complex, made of stromal interaction molecule (STIM) and CRAC modulator (ORAI), have been developed (17, 25, 121). STIM1 serves as an ER-resident Ca^2+^ sensor and also the direct activator of PM-embedded ORAI Ca^2+^ channels (200–209). STIM1-based GECAs, based on either CRY2-STIM or LOV2-STIM hybrid constructs (FIGURE 3*C*), were designed to recapitulate two key molecular steps that drive CRAC channel activation: *1*) the oligomerization of the STIM1 luminal domain to initiate STIM1 activation (210, 211) and *2*) the release of autoinhibitory intramolecular trapping within the STIM1 cytoplasmic domain (212–216). STIM1-based optical actuators enable blue light-gated activation of endogenous ORAI channels to mediate Ca^2+^ influx at varying kinetics (25, 121). The activation and deactivation half-lives fell in the range of 10–50 s and 20–600 s, respectively.

Representative GECAs built upon STIM1 include Opto-CRAC, BACCS (blue light-activated Ca^2+^ channel switch), and LOVS1K with similar designs (22, 23, 217, 218) and optoSTIM1 or its further improved versions (eOS1 and monSTIM1) and CRY2/CIBN-STIM1 variants (197, 214, 219, 220). These tools have been applied to control Ca^2+^-modulated physiological processes in over one dozen cell types and major model organisms, including flies, worms, zebrafish, and rodents. For example, Opto-CRAC was among the first Ca^2+^-dependent optogenetic tools used for lymphocyte activation and dendritic cell-based immunomodulatory therapy in vivo when coupled with NIR light-convertible upconversion nanoparticles (UCNPs) (22). Paired with a CRISPR interference system (221), Opto-CRAC also enabled photoinducible transcriptional reprogramming (222). The BACCS series, designed with a strategy similar to Opto-CRAC but with the best constructs engineered from *Drosophila* Stim, were used to photoexcite olfactory sensory neuron activation and trigger electroolfactogram response in mice (217). OptoSTIM1 expressed in CA1 hippocampus was exploited to selectively reinforce the contextual fear memory with blue light (223). monSTIM1, an ultra-light-sensitive variant of optoSTIM1, was further developed to enable noninvasive optical control of endogenous ORAI channels in deep brain regions, such as the excitatory neurons of the somatosensory cortex and astrocytes in the dentate gyrus and thalamic regions. This tool, when targeted to the anterior cingulate cortex, could photomodulate fear memory in freely moving awake mice (220).

STIM1-based GECAs, nonetheless, depend on endogenous ORAI expression in host cells and also run the risk of causing potential signaling cross talk with other STIM-engaged targets, such as transient receptor potential C (TRPC) channels and voltage-gated Ca^2+^ (Ca_V_) channels (224–228). To avoid these complications, a STIM-independent light-operated Ca^2+^ channel (LOCa; FIGURE 3*D*) was engineered through multiple rounds of high-throughput screening by using Ca^2+^ influx and NFAT nuclear translocation as readouts. LOCa was designed by inserting LOV2 into the intracellular loop of a constitutively active ORAI1 mutant (H171D/P245T), in which LOV2 keeps the channel largely inactive in the dark and photons induce channel activation via allosteric regulation imposed by LOV2 (123). Compared with STIM1-based GECAs, LOCa variants have relatively slower activation kinetics (*t*_1/2,on_ ∼50 s), presumably due to more energy cost required to actuate membrane-embedded hexameric ORAI proteins. LOCa has demonstrated its translational promise in suppressing the aberrant self-renewal of premalignant hematopoietic stem cells, as well as in the mitigation of neurodegeneration in a *Drosophila* model of Alzheimer’s disease (123).

In parallel, an optogenetic device capable of suppressing Ca^2+^ influx has been developed by engineering Ras-like GTPases Rad/Rem/Gem/Kir (RGK), which function as negative regulators of Ca_V_ channels (229). This tool, termed OptoRGK (FIGURE 3*E*), was constructed to conditionally suppress Ca_V_ channel activity by light in a reversible fashion. By replacing the original PM-targeting motif of Rem with SspB and anchoring the LOV2-ssrA to the PM, blue light-induced dimerization of iLID triggered the cytosol-to-PM translocation of Rem (residues 1–266) to suppress rhythmic oscillations of cytosolic Ca^2+^ in cardiac cells (230). OptoRGK promises to find applications in photomodulating many Ca_V_ channel-associated physiological processes in excitable tissues.

#### 4.1.2. Receptor tyrosine kinases.

Given the structural modularity and reliance on dimerization for functional activation, receptor tyrosine kinases (RTKs) are among the most frequently sought membrane receptors for optogenetic engineering. Engineering of photoactivatable receptor tyrosine kinases (Opto-RTKs) was first exploited by fusing AuLOV modules with intracellular catalytic domains from RTKs, including FGFR1, epidermal growth factor receptor (EGFR), rearranged during transfection (RET), and tropomyosin receptor kinase B (TrkB) (139, 231). Opto-RTKs utilize photons rather than natural ligands to precisely activate key downstream signaling events, including PLCγ, phosphatidylinositol 3-kinase/RAC-alpha serine/threonine-protein kinase (PI3K/AKT), and mitogen-activated protein kinase/extracellular signal-regulated kinase (MAPK/ERK) pathways. The use of light, instead of natural ligands, to modulate RTK signaling has the advantage of eliminating potential off-target effects of the ligand and avoiding signaling cross talk. For instance, nerve growth factor stimulates multiple signaling outputs via interaction with both high-affinity (TrkA) and low-affinity (p75NTR, p75 neurotrophin receptor) receptors in neurons. An optogenetic tool, made of AuLOV-TrkA fusion, that specifically engages the TrkA subcircuits was invented to delineate the functional consequence of TrkA activation in cell differentiation without being confounded by the parallel p75NTR signaling pathway (232).

**FIGURE 4.**
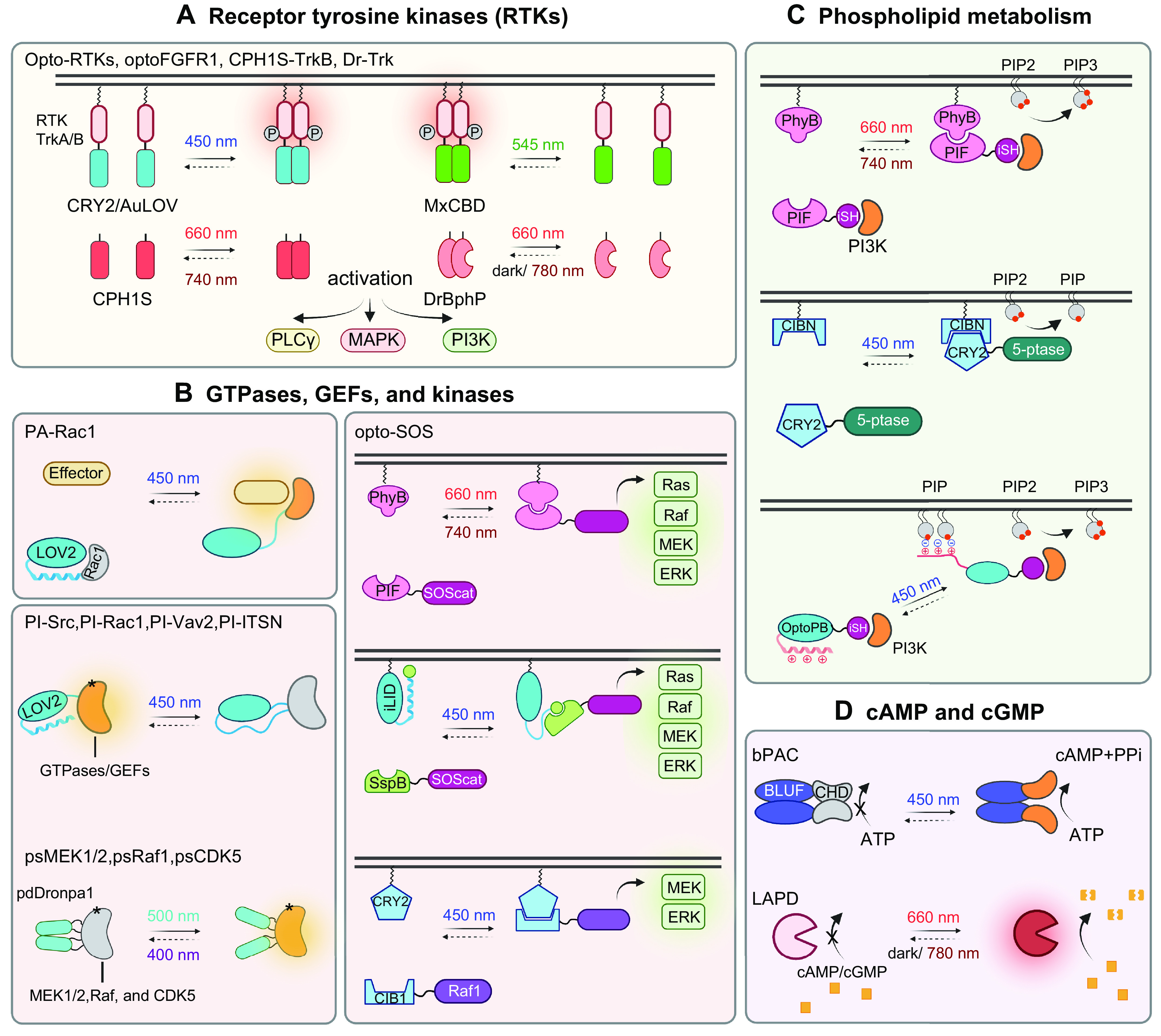
Design of light-sensitive RTKs, signaling messengers, and proteins or enzymes. *A*: light-induced association or dissociation of the intracellular domains of receptor tyrosine kinases (RTK-ICDs; such as FGFR and Trk) allows optical interrogation of downstream PLCγ, MAPK, and PI3K pathways. RTK-ICDs are fused to photosensory modules that respond to blue (CRY2 and AuLOV), green (MxCBD), red (CPH1S), or far-red (DrBphP) light, respectively. *B*: photoactivation (PA) or inhibition (PI) of GTPases, GEFs, or kinases. *C*: optogenetic control of phospholipid metabolism by inducible recruitment of phosphoinositide-metabolizing enzymes (PI3K or 5-ptase) to the PM or subcellular compartments. *D*: photoactivatable adenylate cyclase homology domain (CHD) of bPAC catalyzes cAMP production in response to blue light stimulation, whereas red light-activated LAPD promotes cAMP or cGMP degradation. See glossary for abbreviations. Image created with BioRender.com and used with permission.

A number of different photosensory domains have been fused to the RTK intracellular kinase domains to generate hybrid proteins that respond to blue (optoFGFR1) (139, 231), green (FGFR1-MxCBD) (93, 233), or red/infrared (CPH1S-TrkB, Dr-TrkA/B) (109, 191) light. A PM anchor, derived from the Lyn PM-targeting motif, is frequently required to maintain proper subcellular localization of engineered photoresponsive RKTs. Light stimulation can either initiate or terminate downstream signaling pathways (FIGURE 4*A*).

#### 4.1.3. GTPases, GEFs, and kinases.

GTPases are a large family of enzymes that bind to GTP and catalyze the hydrolysis of GTP into GDP. Guanine nucleotide exchange factors (GEFs) promote GTPase activity by stimulating the dissociation of GDP to allow subsequent GTP binding. GTPases and GEFs work cooperatively to function as molecular switches for many fundamental cellular processes, including receptor-mediated signal transduction and cell migration. Three general approaches have been applied to photomanipulate GTPase signaling that involves both small GTPase and GEFs (FIGURE 4*B*): *1*) reversible masking of GTPase activity via fusion with LOV2, as best exemplified by PA-Rac1 (116); *2*) introduction of extrinsic allostery into GTPases or GEFs via LOV2 insertion (126); and *3*) inducible delocalization of catalytic segment (SOScat) of the protein Son of sevenless (SOS) to a new subcellular destination (opto-SOS) (66, 234, 235).

PA-Rac1 was designed to photoactivate Rac GTPase for directed cell protrusion in a blue light-dependent manner (FIGURE 4*B*), whereas a photo-inhibitable Rac1 (PI-Rac1) was shown to inactivate the GTPase activity and caused light-inducible cell edge retraction (126). In another example, a red/infrared-light controllable Ras GTPase activation system (opto-SOS; FIGURE 4*B*) was developed. The recruitment of SOScat to the plasma membrane induces Ras/ERK activation. By fusing PhyB to a membrane-targeting peptide and PIF6 to SOScat, red light-induced PhyB-PIF6 interaction leads to the delocalization of cytosolic SOScat to the PM and induces ERK activation. Coupled with proteomic profiling, opto-SOS was used to decode various temporal patterns of Ras dynamics into distinct signaling programs and physiological outputs. Through a frequency-response analysis using different light pulses (minutes to hours), paracrine STAT3 activation was suggested to be critical for maintaining sustained Ras activation (234). Additionally, similar Opto-SOS systems using iLID-CAAX and SspB-SOScat linked by a P2A cleavable peptide (236) or using a REDMAP (red/far-red light-mediated and miniaturized PhyA-based photoswitch) system (176) have been created to achieve light-induced ERK activation in *Drosophila* and mammals.

Similar strategies, including subcellular delocalization and LOV2 insertion, were applied to engineer various photocontrollable kinases (FIGURE 4*B*). For instance, light-triggered membrane localization of Raf1 kinase was explored to recapitulate the RAF-MEK-ERK kinase signaling cascade (66, 235). LOV2 insertion into exposed surface loops of a constitutively active Src kinase mutant (Y535F) led to the development of a photoinhibitable Src (PI-Src). PI-Src was shown to cause a collapse of lamellipodia in a light-dependent manner (126). A more generalizable approach involves the use of pdDronpa1, which can be conveniently fused to both ends of kinases, such as MEK1/2, Raf, and CDK5, to bidirectionally turn on and off the kinase activity with light emitting at 500 nm and 400 nm, respectively (88). These tools provide new opportunities to interrogate the spatial and temporal aspects of GTPase and kinase signaling in living cells with high precision.

#### 4.1.4. Phospholipid metabolism.

Phosphoinositides (PIs) are integral lipid components of cell membranes that regulate a wide variety of cellular functions. PI3K is involved in the biogenesis of phosphatidylinositol 3,4,5-trisphosphate [PI(3,4,5)P_3_, or PIP_3_], which subsequently recruits the AKT kinase to the PM to phosphorylate many downstream effectors required for cell proliferation, growth, and survival (237). Light-controlled activation of PI3K/PIP_3_ was achieved by using PM-anchored PhyB to induce translocation of the SH2 region of the PI3K-p85α regulatory subunit fused to PIF6 (108), which in turn recruited the PI3K-p110α catalytic subunit to catalyze PIP_3_ production (FIGURE 4*C*). A similar approach using the CRY2-CIBN pair was utilized to control phosphoinositide metabolism in the plasma membrane. By fusing CRY2 to the phosphatidylinositol 5-phosphatase modules of OCRL or INPP5E, CIBN-CAAX-mediated light-triggerable membrane recruitment of 5-phosphatases led to dephosphorylation of PI(4,5)P_2_ (PIP_2_) and PIP_3_ (238). Furthermore, the OptoPB tool could be used for photoinducible cytosol-to-PM translocation of PI-modifying enzymes to reprogram PI metabolism (160). Collectively, by selectively recruiting phospholipid-metabolizing enzymes to a specific subcellular localization, light can be harnessed to precisely reprogram the phospholipid compositions in the cell membrane.

#### 4.1.5. cAMP and cGMP.

Light-activated proteins have been discovered to modulate the generation of second messengers, such as cAMP, cGMP, DAG, or inositol trisphosphate (IP_3_). The production of IP_3_ and DAG often arises from PIP_2_ hydrolysis, a reaction triggered by photoinducible activation of RTKs or GPCRs as described above. By contrast, the photoinducible biogenesis of cAMP was controlled by a family of soluble PACs.

A typical PAC enzyme has a BLUF photo-sensing domain linked to an adenylyl cyclase domain and mediates the conversion of ATP to cAMP upon light stimulation (FIGURE 4*D*). For example, *Beggiatoa* PAC (bPAC) has been used to modulate cAMP production in sensory neurons across different species (239). bPAC can be further coupled with cyclic nucleotide-gated channels (CNGCs) to photoregulate ion channel activity in rat hippocampal pyramidal neurons and to impact grooming behavior in *Drosophila* (240). In sperm, bPAC was ectopically expressed to photoactivate cAMP production to mimic endogenous adenylyl cyclase activity, which is required for sperm swimming and oocyte fertilization (241). bPAC was also spatially constrained to the primary cilium to demonstrate that ciliary but not extraciliary cAMP inhibits hedgehog signaling transduction (242).

In parallel, an engineered light-activated phosphodiesterase (LAPD; FIGURE 4*D*) was developed to photo-manipulate the hydrolysis of cAMP and cGMP with red light (243). LAPD consists of a phytochrome light-sensing domain and the catalytic domain of human phosphodiesterase 2A. In theory, LAPD and bPAC can be used in parallel to enable cAMP biogenesis with blue light and cAMP degradation with red light. Given its deeper tissue penetration capability, red/infrared light-responsive LAPD appears to be an attractive tool for studying cAMP/cGMP-associated physiological processes in vivo.

### 4.2. Cytoskeleton Dynamics and Cellular Mechanics

**FIGURE 5.**
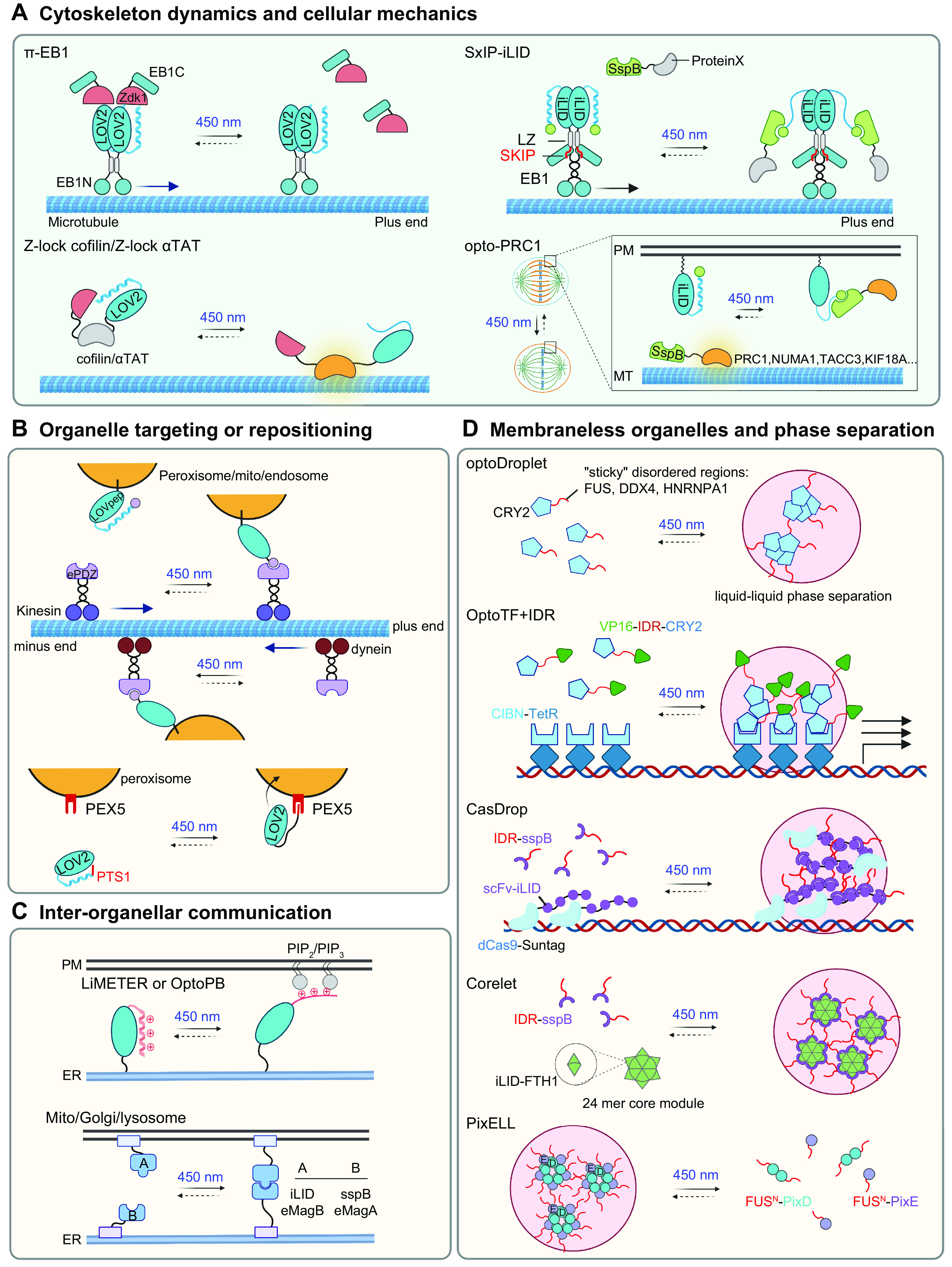
Optogenetic control of cytoskeleton and subcellular compartments. *A*: manipulation of microtubule (MT) plus end dynamics by optogenetic engineering of MT binders. In π-EB1, the EB1 NH_2_-terminal tubulin-binding domain and the COOH-terminal (EB1C) plus end tracking protein (+TIP) binding domain are fused to the LOVTRAP pair (LOV2 and Zdk1, respectively). The SxIP-iLID system consists of an iLID-fused EB-binding Ser-x-Ile-Pro (SxIP; SKIP in the cartoon) motif and sspB-fused protein X. In another design, LOVTRAP serves as a lock (via LOV2-ZDK1 interaction or Z-lock) to reversibly block the binding of actin-remodeling proteins, such as cofilin and the α-tubulin acetylase αTAT. Using iLID, opto-PRC1 dislocates PRC1 from mitotic spindle to the PM, thereby disturbing the proper alignment of kinesins and modulators (e.g., NUMA1, TACC3, or KIF18A) to the metaphase plate and ultimately destabilizing the kinetochore fibers. *B*: use of TULIP (the LOVpep-ePDZ pair; *top*) to enable light-controllable organelle repositioning and cargo protein transportation to subcellular organelles. In a second design, LOV2-PTS1 (*bottom*) is designed to allow light-inducible exposure of a peroxisome targeting sequence (PTS) with subsequent delivery of POIs into peroxisome. *C*: ER-anchored OptoPB/LiMETER (*top*) and membrane-bound optical dimerizers (*bottom*) were applied to enable photoswitchable membrane tethering and intermembrane contact formation. *D*: light-induced condensate formation is achieved by *1*) CRY2olig-mediated clustering (optoDroplet) of IDPs or IDRs (e.g., FUS, DDX4, HNRNPA1); *2*) conditional self-assembly of 24-mer core modules using iLID-FTH1/IDR-sspB (Corelet); *3*) a PixE/PixD dissociation system (PixELL) that disrupts FUS condensates upon light illumination. Light-driven condensate formation is also utilized to modulate gene transcription when coupled with transcription factors (OptoTF) or dCas9-Suntag (CasDrop). See glossary for abbreviations. Image created with BioRender.com and used with permission.

Cytoskeleton dynamics regulate intracellular cargo transport, organelle positioning, chromosome segregation, cell motility, and directed migration (244). A variety of optogenetic tools have been developed to manipulate microtubule (MT) and actin dynamics (245). MT end-binding protein 1 (EB1) mediates the binding of MT plus-end-tracking proteins (+TIPs) to the growing MT plus ends. Using the LOVTRAP system, an engineered π-EB1 (FIGURE 5*A*) was crafted to photodissociate EB1 and +TIPs and inhibit MT growth (246). In π-EB1, LOV2 is fused to the plus end-recognizing NH_2_-terminal CH domain of EB1. A GCN4 leucine zipper is inserted in between to enhance EB1 dimerization and efficient plus end tracking. Zdk1 is fused to the EB1 COOH-terminal EBH domain, which is responsible for recruiting +TIPs, such as mitotic centromere-associated kinesin (MCAK), CLIP-associated protein 2 (CLASP2), and SLAIN motif-containing protein 2 (SLAIN2). Under blue light, +TIPs dissociate from the MT plus end and cause a rapid inhibition of MT growth and change in the direction of cell migration. Another tool, SxIP-iLID (FIGURE 5*A*), was designed in a slightly different manner to enable light-inducible recruitment of targets to the growing MT plus ends to perturb cytoskeleton dynamics (247). For instance, SxIP-iLID was used to enable temporally controlled recruitment of an F-actin binding domain to MT plus ends, thereby cross linking the MT and actin networks to generate a peripheral MT exclusion zone.

Actin cytoskeleton dynamics is tightly coordinated by various Rho GTPases and their cognate GEFs. The aforementioned PA-Rac1 was among the first applied to reversibly control migration directionality and cell protrusion (116). Inducible membrane recruitment of GEFs or GTPases per se is a second effective means adopted to activate Rho GTPases (Rac1, Cdc42, and RhoA), thereby enabling cell shape remodeling by blue or red light (102, 103, 184, 248–252). In a third approach, LOVTRAP was repurposed to generate two photoswitchable actin-remodeling proteins, Z-lock cofilin and Z-lock α tubulin acetyltransferase (αTAT) (253). Z-lock modules were engineered into cofilin (an actin-severing protein) and αTAT as single-chain polypeptides to sterically block their active sites in the dark (FIGURE 5*A*). Blue light irradiation effectively restored the function of cofilin to produce protrusions and invadopodia and reactivated αTAT to catalyze tubulin acetylation.

Chromosome alignment at the spindle equator is maintained by the plus end of kinetochore fiber (k-fiber) and polar ejection force. In addition, bridging fibers, which refer to MT bundles associated with kinetochores that bridge sister k-fibers in metaphase, provide a centering force on the kinetochore. A series of optogenetic tools have been devised to probe kinetochore dynamics and spindle formation at high spatial and temporal resolution (FIGURE 5*A*). Acute delocalization of a cross linker of bridging fibers, protein regulator of cytokinesis 1 (PRC1), from the central region of the metaphase spindle to the PM by using iLID could disturb the alignment of the kinetochores on the metaphase plate through dissociation of kinesin 4 and 8 (254). Delocalization of other protein modulators (e.g., nuclear mitotic apparatus protein, transforming acidic coiled-coil-containing protein 3, and KIF18A) was similarly attempted to perturb mitotic spindle positioning and probe the mechanics of mitosis in living cells (255–257).

### 4.3. Organelles and Subcellular Compartments

#### 4.3.1. Organelle targeting or repositioning.

Organelle transport and positioning, a process actively facilitated by the cytoskeleton and motor protein complexes, is intimately involved in regulating cell signaling, metabolic response, growth, and polarity (258–262). Optical dimerizers have been utilized to control the interaction between selected organelles and specific cytoskeletal or motor proteins, their adaptors, cross linkers, and modulators (FIGURE 5*B*). Optogenetics makes it possible to precisely manipulate the subcellular distribution of a subpopulation of organelles without affecting other organellar compartments (262, 263). For example, the redistribution of peroxisomes from the cell center to peripheral areas was achieved in cells coexpressing PEX3-LOVpep and an MT plus end-directed kinesin fused with ePDZ (KIF1A-ePDZb1) (264). On the contrary, fusing minus end-directed dynein activating adaptor, the NH_2_-terminal domain of Bicaudal-D2, to ePDZb1 induced accumulation of peroxisomes at the center of the cells. Transportation of peroxisomes could thus be arrested or directed from light-activated areas to nonexposed areas with superior spatiotemporal precision. Furthermore, recruitment of a myosin-Vb-ePDZb1 to engineered peroxisomes could stall their movement at the actin-rich cell cortex. Similar manipulations can be attained with endosomes and mitochondria. The light-directed local positioning of recycling endosomes was further shown to induce reversible axon growth in primary rat hippocampal neurons. Likewise, Tom20-LOVpep was coupled either with KIF1A-ePDZb1 or syntaphilin-ePDZb1 to modulate axonal mitochondria motility in neurons, which will likely find uses in remote interrogation of axon regeneration, synaptic homeostasis, and neurodegeneration (259, 265). Similar organelle-repositioning tools could be designed by replacing TULIP with the CRY2-CIBN optical dimerizer and using slightly different organellar anchors, motor proteins or their adaptors (266).

An alternative approach for subcellular organelle targeting relies on the use of LOV2 to cage specific organelle-targeting sequences (FIGURE 5*B*). For instance, a peroxisome targeting sequence (PTS1) has been fused with LOV2 to induce peroxisomal protein transport (267). Under blue light stimulation, uncaged PTS1 restored its interaction with peroxisomal import receptor PEX5 to mediate cargo transport from the cytosol to the peroxisome.

Collectively, these organelle-targeting or -delocalizing optogenetic tools are superior over traditional pharmacological or chemical biology approaches by not only offering high spatiotemporal precision but also allowing the direct dissection of the causal effects between organelle positioning/movement and cellular function in real time.

#### 4.3.2. Interorganellar communication.

Membrane contact sites (MCSs) are specialized subcellular compartments dynamically assembled between closely apposed organelles with a gap distance ranging from 10 to 40 nm (268–270). MCSs play critical roles in Ca^2+^ and reactive oxygen species signaling, lipid metabolism, membrane biogenesis, autophagy, and cellular stress responses. In situ proximity biotinylation-based proteomics approaches have been widely used to identify the molecular compositions at the MCSs (162, 271, 272). In parallel, optogenetic tools capable of artificially including member tethering or intermembrane contacts have recently been developed to examine the functional consequences of MCS perturbation (FIGURE 5*C*) (163). For instance, the aforementioned photoswitchable ER-PM tether, LiMETER or OptoPBer, takes advantage of LOV2-caged polybasic (PB) C-tails derived from a small GTPase or STIM1, which are known to interact with PI(4,5)P_2_ and PI(3,4,5)P_3_ residing in the inner leaflet of PM. By grafting the LOV2-PB domain to the ER-resident STIM1 scaffold devoid of the luminal domain and ORAI-activating cytosolic region, LiMETER enabled light-inducible tuning of the gap distance between ER-PM contact sites to regulate ORAI distribution on the PM and impact Ca^2+^ signaling (160). In addition, the iLID-sspB pair has been used to photoinduce intermembrane tethering at the ER-mitochondria interfaces. In this case, outer mitochondrial membrane (OMM)-targeted bacterial actin nucleator protein ActA and an ER anchor peptide sequence from cytochrome *b*5 (CB5) were fused to iLID and SspB, respectively (273). Likewise, the VVD-based eMags optical dimerizer was employed to trigger interorganelle tethering with high spatial and temporal resolution by light (158). eMags was applied to reconstitute ER-Golgi tethering involved in phosphatidylinositol-4-phosphate (PI4P) transport and metabolism. These optogenetic tools allow in situ perturbation of MCSs in real time to greatly benefit the mechanistic dissection of MCSs under most physiologically relevant conditions.

#### 4.3.3. Membraneless organelles and phase separation.

Traditional intracellular organelles are often bound by membranous structures. However, biomolecules can also be organized into subcellular compartments in the form of cellular bodies that are membraneless and exhibit liquid-liquid phase separation (LLPS) properties. Notable examples include P granules, stress granules, and nucleoli that mediate spatial patterning of cells, transcriptional regulation, and innate immune sensing (274–276). These membraneless organelles usually exist as liquid droplet-like condensates that arise from multivalent interactions between clusters of intrinsically disordered proteins or regions (IDPs or IDRs) and/or nucleic acids. Synthetic membraneless organelles recapitulating these features can be conveniently assembled by utilizing optogenetic techniques (FIGURE 5*D*). Optogenetics is found to be extremely useful to quantify the threshold concentration at the boundaries that triggers LLPS and to reversibly mimic condensate formation mediated by many IDPs or IDRs (277).

Spatiotemporal control of intracellular phase transition was first made possible by using optoDroplet (137). The OptoDroplet platform utilizes CRY2 to drive reversible oligomerization of IDR-containing ribonucleoprotein body proteins, such as FUS, DDX4, and HNRNPA1, in a light-dependent manner. Fusing FUS to CRY2olig could increase the strength for clustering but slow the inactivation kinetics, thus leading to gellike structure formation. Further light-induced deep supersaturation was found to promote the formation of irreversible aggregates reminiscent of pathological aggregates seen in neurodegenerative diseases.

With a similar design principle, OptoTF was developed to enable light-induced IDR oligomerization at the promoter region to drive LLPS and perturb gene transcription (278). In this case, IDRs were inserted into a transcription-activating construct, CRY2-eYFP-NLS-VP16, whereas CIBN-TetR was used for tetracycline response element (TRE) binding (FIGURE 5*D*). Light-induced TF droplet formation at the target promoters was shown to promote gene transcription activation by up to fivefold. Building upon the CRISPR-Cas9 system, CasDrop was designed to target endogenous genomic loci and control the local concentration of transcriptional regulators and nuclear proteins through phase separation. CasDrop contains a catalytically dead Cas9 (dCas9)/single guide RNAs (sgRNAs) for precise genomic loci targeting and an iLID module to recruit SspB-IDR-containing nuclear proteins, such as bromodomain-containing protein 4 (BRD4), Fused in sarcoma (FUS), and TATA-binding protein-associated factor 2N (TAF15). CasDrop allows light-driven association between sgRNA-targeted distal genomic loci (such as enhancer and telomere) while filtering out nontargeted background components of the genome (279). This chromatin filter model likely explains how phase separation in the nucleus restructures the genome organization to impact transcriptional regulation in eukaryotes.

In addition, a two-component Corelet system was developed to enable light-activated droplet condensation (FIGURE 5*D*). Corelet contains SspB tagged to various self-interacting IDRs and 24 subunits of the human ferritin heavy chain (FTH1) fused with iLID that self-assemble to a spherical particle core. Corelet was used to illustrate a diffusive capture mechanism where slowly diffusing multivalent interactions can amplify the IDR concentration through posttranslational modifications (e.g., phosphorylation) to drive local condensation and phase separation (280).

A PixE/PixD-dependent synthetic device, Pix Evaporates from Liquid-like droplets in Light (PixELL; FIGURE 5*D*), was generated to confer light control over liquid droplet dissociation (186). The PixELL system self-assembled into condensates in the dark and dissociated into soluble FUS-PixD dimer and FUS-PixE monomers upon light stimulation. Both the optoDroplet and PixELL systems have been applied to unveil the biophysical principles governing spatial memory of phase-separated structures and dissect the diffusion dynamics of different subcellular compartments (186).

### 4.4. Autophagy

Autophagy has been well recognized as the intracellular recycling machinery responsible for the clearance of pathologically accumulated tau protein in neurons from AD patients. Transcription factor EB (TFEB) is a well-known regulator that induces autophagy-mediated clearance of pathological tau. To confer optical control over TFEB transcription, a DNA-binding EL222 with an NH_2_-terminal VP16 transcriptional activation domain was coexpressed with a light response element encoding TFEB. Light-inducible expression of TFEB has been shown to clear tau in neurons derived from human induced pluripotent stem cells (281).

The mitochondrion is the cellular powerhouse and a metabolic hub that provides energy to maintain normal cell function. Accumulation of damaged mitochondria due to aging or infection triggers turnover of mitochondria by autophagosomes, a process known as mitophagy (282). Activating molecule in Beclin 1-regulated autophagy protein 1 (AMBRA1) is an autophagy regulator that can unleash LC3-dependent mitophagy once localized to the OMM (283). By fusing AMBRA1 to SspB and anchoring iLID to OMM, light stimulation forced cytosol-to-OMM translocation of AMBRA1 to induce mitophagy in T cells, as well as in zebrafish (284). In addition, fusion of a mitochondrial leading sequence (MLS) of the ATP binding cassette transporter ABCB10 to ChR2 promotes the targeting of ChR2 to inner mitochondrial membrane (IMM). As a result, blue light triggers the inner membrane potential (ΔΨ_m_) depolarization to regulate mitophagy (285).

Nuclear p53 induces autophagy by transcriptionally activating the expression of genes that are associated with autophagy activation (286, 287). p53 is also important for the nucleus-to-cytoplasm shuttling and autophagy-mediated degradation of Ten-eleven Translocation 2 (TET2). The accumulation of nuclear TET2 in p53-mutated/null tumors protects cancer cells from chemotherapy-induced DNA damage, hence rendering drug resistance to cancer cells (288). By adapting CRY2 with a cytoplasm-localized p53 (cyto-P53), in the presence of blue light, a CIB1-NLS promotes the nuclear translocation of CRY2-cyto-P53 and LC3^+^ autophagosome formation (289). Such tools will likely find applications to modulate p53-related autophagic regulation pathways.

### 4.5. Photoactivatable Recombinases

Cre and Flp recombinases catalyze sequence-dependent recombination of the genome and are widely used in the generation of transgenic rodent models. Engineered Cre and Flp enzymes have been generated to confer optical control over genome engineering. Optical dimerizers, made of either CRY2-CIB (63) or pMag-nMag pairs (290, 291), have been used to restore the functional reassembly of split Cre recombinases with light. The second generation of photoactivable Cre was further evolved by using a long-cycling L348F mutant of CRY2 (65, 292), which led to 35% increase of Cre recombinase activity with 50% reduction in the dark activity. The Magnets-based Cre system was further demonstrated to mediate efficient DNA recombination in living mice, establishing the feasibility of optogenetic genome engineering in vivo (293).

### 4.6. Immunomodulation

**FIGURE 6.**
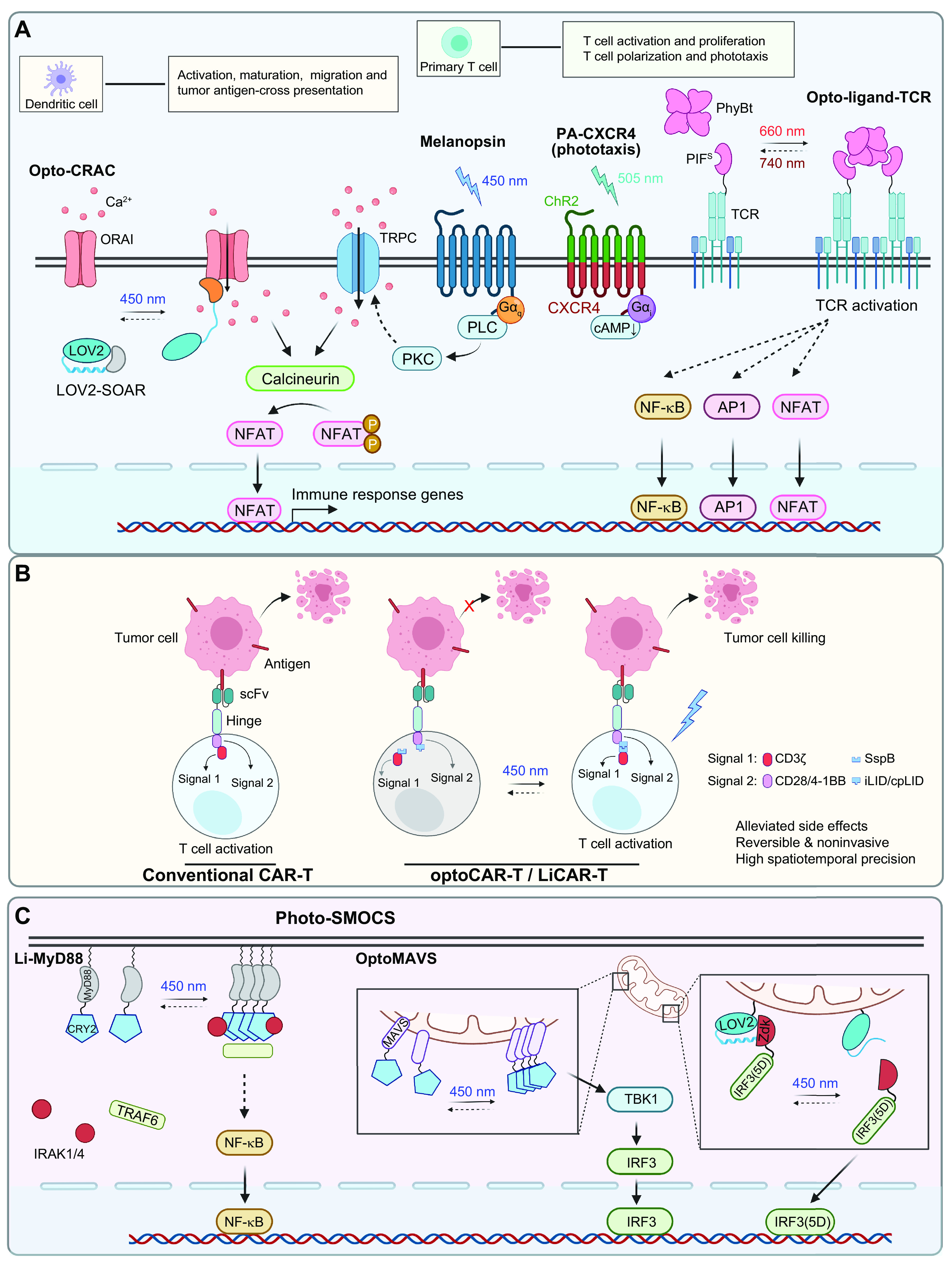
Optogenetic immunomodulation and light-switchable CAR-T cells. *A*: optogenetic tools designed to modulate immune responses. *1*) Optogenetic control of Ca^2+^ signaling by Opto-CRAC or melanopsin to promote the antitumor effector functions in dendritic cells (DCs) and T lymphocytes; *2*) light-triggerable T-cell trafficking mediated by PA-CXCR4, which is created by swapping the intracellular rhodopsin-associated Gα_t_ with the CXCR4-associated Gα_i_ domain; *3*) a red light-sensitive PhyB-tetramer (PhyBt) serves as photoligand for secretory PIF (PIF^S^)-fused TCR (opto-ligand-TCR) to study the kinetics of TCR activation by tuning the light pulse intensity and duration. *B*: design of optogenetic or light-switchable CAR-T (OptoCAR-T or LiCAR-T) cells. Two classical activation signals are required to mount T cell-mediated productive immune response. In OptoCAR-T, domains mediating the 2 signals (CD28/4-1BB or CD3ζ) can only be reassembled to form a functional CAR upon photostimulation, thereby providing a safety switch for reversible CAR-T cell activation to mitigate potential side effects. *C*: light-controllable activation of SMOCs (photo-SMOCs: MyDDosome and MAVSome) to modulate innate immune signaling. In addition, light-induced type I IFN activation can be achieved by using LOVTRAP-mediated mitochondria-to-nucleus shuttling of IRF3(5D). See glossary for abbreviations. Image created with BioRender.com and used with permission.

Immunomodulation is a dynamic process whereby innate and adaptive immunity can be synthetically or genetically modulated for host defense (294) and affect trafficking and persistence of cytotoxic T cells to reverse immunosuppression induced by tumor in the tumor microenvironment (17). The combination of nanophotonics, optogenetics with immunoengineering, has enabled wireless control over both innate and adaptive immunity (FIGURE 6). We highlight here the latest progress in optogenetic immunomodulation and discuss the potential applications of optogenetics for future immunotherapy.

Ca^2+^ flux and downstream signaling is important for both innate and adaptive immune effector functions (22). Ca^2+^ mobilization in dendritic cells (DCs) is known to boost DC activation, maturation, and migration (295, 296). Opto-CRAC expressed in DCs could photoinduce Ca^2+^ influx, promote tumor antigen cross presentation to CD8^+^ T cells, and boost antitumor immune response both in vitro and in a mouse model of melanoma (FIGURE 6*A*) (22). Opto-CRAC has also been engineered into primary T cells to mount productive immune response ex vivo in a light-dependent manner in the presence of costimulatory signals. Nonetheless, since constitutive NFAT activation is known to cause T-cell exhaustion or anergy by binding at genomic regions that do not require cooperation with AP-1 (297), sustained Ca^2+^ elevation mediated by Opto-CRAC or similar tools will likely lead to similar phenotypes. This opens the exciting possibility of photoconverting effector T cells into exhausted ones to calm overactive immune responses in some autoimmune and inflammatory disorders.

Melanopsin, when ectopically expressed in T cells, mediates light-inducible intracellular Ca^2+^ mobilization through TRPC channels (FIGURE 6*A*), thereby triggering NFAT-mediated cytokine expression to boost effector T-cell function and facilitate solid tumor killing in a mouse model of hepatocellular carcinoma (298). Another approach called the opto-ligand T-cell receptor (TCR) system has been used to tune the ligand-TCR binding kinetics by using the red light-sensitive PhyB-PIF pair (299). This optogenetic system allows precise control of the duration of ligand-TCR binding and therefore has been used to demonstrate a kinetic proofreading model for TCR activation (FIGURE 6A), in which the half-life of the ligand-TCR interaction dictates the degree of downstream TCR signaling activation (300).

To control T-cell phototaxis, an opsin-chemoreceptor chimera (PA-CXCR4; FIGURE 6*A*) made of ChR2-CXCR4 fusion has been generated by retaining the extracellular photosensitive domain of ChR2 and replacing the intracellular rhodopsin-associated Gα_t_ with CXCR4-associated Gα_i_. Activation of PA-CXCR4 was shown to induce T-cell polarization-associated small GTPase activation in vitro and T-cell migration to aid tumor killing in vivo (301).

Optogenetics has also been applied to control the activity of therapeutic immune cells, such as the chimeric antigen receptor (CAR) T cells. Optogenetic or light-switchable chimeric antigen receptors (optoCARs or LiCARs; FIGURE 6*B*) based on iLID, cpLID (cpLOV2-ssrA and -sspB), or CRY2-CIB1 have been developed to conditionally reconstitute a split CAR into a fully functional CAR in the presence of blue light (23, 302, 303). In the dark, the tumor antigen-sensing unit and/or the effector domains remain separately expressed in two constructs. Upon light stimulation, optical dimerizers bring the two split units together to reassemble intact CARs to mount antitumor immune response. By simply tuning the temporal frequency of light input in optoCAR-T cells, one could faithfully mimic the dynamic interaction patterns T cells might encounter naturally in the human body (302). OptoCAR has been utilized to reveal that T cells can selectively filter oscillatory signals on the timescale of minutes. Furthermore, CD8^+^ optoCAR-T cells have demonstrated high spatial and temporal precision in terms of tumor killing (23, 303). The most noteworthy feature of optoCAR and LiCAR is their reversibility and tunability, promising to alleviate side effects associated with conventional CAR-T cell therapy, including cytokine release syndrome and “on-target off-tumor” toxicity (304, 305).

In addition to adaptive immunity, innate immune signaling pathways have been engineered to respond to light. Pattern recognition receptors are important innate sensors for the clearance of foreign stimuli such as viruses, bacteria, and toxic substances causing chronic inflammation and autoimmune diseases (306–308). The spatiotemporal activation of supramolecular organizing centers (SMOCs) made of oligomeric protein complexes, including MyDDosome, MAVSome, and inflammasome, is critical for host defense and immunometabolism (309). CRY2 or its variants have been fused to SMOC components (photo-SMOCs) to induce innate immune responses (FIGURE 6*C*). For instance, CRY2-mediated oligomerization of membrane-tethered myeloid differentiation primary response protein 88 (MyD88) could activate NF-κB signaling upon photostimulation (310, 311). Similarly, light-triggered oligomerization of mitochondrial antiviral-signaling protein (MAVS) or TNF receptor-associated factor 6 (310, 311), together with CRY2/CIB1-mediated dimerization of DNA-dependent activator of interferon (IFN)-regulatory factor (DAI) (312), have been explored to photoinduce activation of type I IFN and/or NF-κB signaling pathways. Furthermore, light-controllable nucleocytoplasmic shuttling of constitutively active IRF3, IRF(5D), was shown to conditionally activate type I IFN signaling in cells of the immune system (311). In the forthcoming years, more and more synthetic biology tools tailored for optical control of innate and adaptive immunity will be developed to enable versatile optogenetic immunomodulation.

### 4.7. Molecular Recorders

**FIGURE 7.**
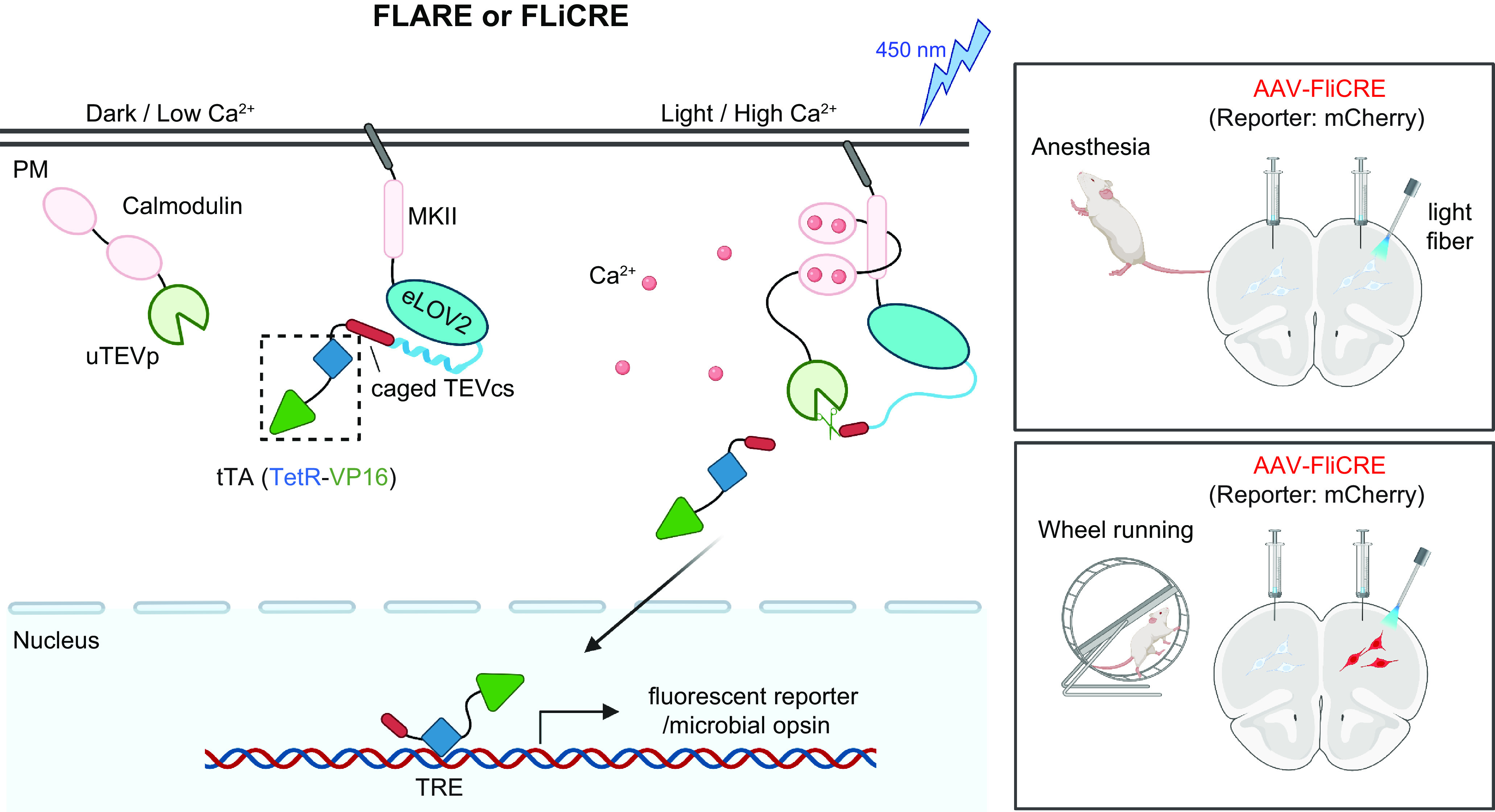
A light- and Ca^2+^-gated cell tagging and recording system (FLARE or FLiCRE). Compared with the dark or low-Ca^2+^ (nonneuronal activation) state, light illumination and high Ca^2+^ (neuronal activation) level trigger the interaction between calmodulin and the PM-tethered MKII peptide, accompanied by conformational changes in an evolved LOV domain (eLOV) to expose the TEV cleavage site (TEVcs). This allows the calmodulin-linked protease uTEVp to act on TEVcs to release the tetracycline (TET)-response element (TRE)-binding transcription factor tTA (TetR-VP16). TetR-VP16 subsequently translocates to the nucleus to switch on the transcription of a fluorescent reporter (for cell labeling and transcriptome recording) or microbial opsin (for reactivation or replay of the FLiCRE-expressing cells). See glossary for abbreviations. Image created with BioRender.com and used with permission.

Cell tagging has been used for spatiotemporally labeling cell types in organoids and isolated tumors with photocage compounds, such as near-UV light-responsive calcein nitroveratryloxycarbonyl caging dye. This photopharmacology method allows spatially resolved live cell tagging and isolation in a nondestructive manner using protected photoactivatable cell dyes (313). To tag cells dependent on their activities and study the causal relationship in the cell circuits with genetically encoded tools, a robust tool designated Fast light- and Ca^2+^-regulated expression (FLiCRE; FIGURE 7) has been developed to convert the transient activation signal (Ca^2+^ influx) in neurons into a stably expressed transcript, such as a reporter gene (314).

FLiCRE serves as a molecular recorder to store the history of activity and facilitate the visualization or sorting of individual cells for subsequent single-cell RNA sequencing analysis. FLiCRE is well suited to studying the interactions between activated and nonactivated cells in the same timescale or long-range cell-to-cell communications in neural circuits. FLiCRE is evolved from its prototype Fast light- and activity-regulated expression (FLARE) (315), containing a PM-anchored transcription factor (such as Gal4), in between inserted with a calmodulin-binding peptide (MKII), a mutated form of LOV (hLOV1) with faster kinetics and better caging capability, and a tobacco etch virus cleavage site (TEVcs). A coexpressed construct contains an ultra-TEV protease (uTEVp) fused to calmodulin that binds to MKII when it senses high levels of Ca^2+^. In the dual presence of blue light and high Ca^2+^, calmodulin engages MKII and brings TEV in close proximity to the TEVcs site, the latter of which is only accessible by TEV in the lit state (FIGURE 7). After TEV-catalyzed cleavage, Gal4 undergoes nuclear translocation to drive synthetic gene expression. Gal4 can be further replaced by a tetracycline-controlled transactivator (tTA), tetR-VP16 as the transcription factor to activate the expression of a fluorescent reporter or a microbial opsin (red light-sensitive ChrimsonR or bReaChES) under the control of a tetracycline-regulatable element. In this way, neural activity can be recorded by a fluorescent reporter and replayed (reactivated) by a second red light stimulation through ChrimsonR or bReaChES. With an adeno-associated virus (AAV) delivery system, FLiCRE has been used to label and identify neuronal subtypes in nucleus accumbens that are coupled to long-range excitatory inputs and downstream of transsynaptically activated axons (314).

### 4.8. Programmed Cell Death

**FIGURE 8.**
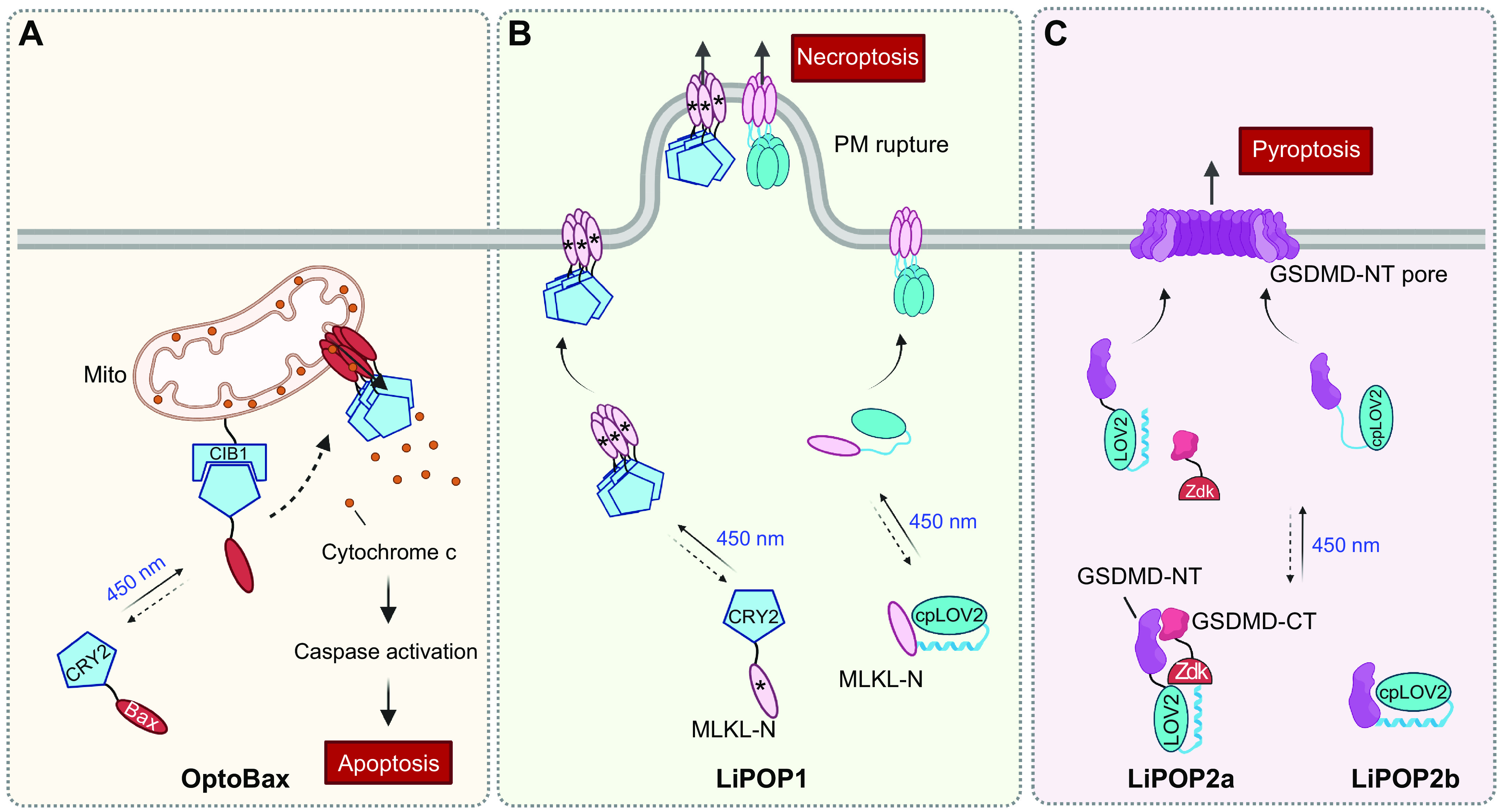
Optical control of programmed cell death. *A*: OptoBax induces cytochrome *c* release and cell apoptosis via light-triggered CRY2-Bax mitochondrial translocation and oligomerization through interaction with the mitochondria-localized CIB1. *B*: LiPOP1 triggers oligomerization (by CRY2) or uncaging (by cpLOV2) of a constitutively active MLKL-N to induce PM rupture and necroptosis. *C*: LiPOP2a containing LOV2-GSDMD-NT and Zdk-GSDMD-CT undergoes light-dependent dissociation to release the active GSDMD-NT to form pore on PM, leading to cell pyroptosis. In an alternative design, LiPOP2b uses cpLOV2 to cage GSDMD-NT in the dark and induce pyroptosis following blue light illumination. See glossary for abbreviations. Image created with BioRender.com and used with permission.

Among all the cell death pathways, apoptosis is initiated and executed by a series of cysteine-aspartic proteases (caspases) (e.g., caspase-3, -7, -8, and -9) and proapoptotic members of the Bcl-2 family of proteins (e.g., Bid and Bax) (316–318). In response to apoptotic signals, Bax translocates from the cytosol to OMM, where it oligomerizes to form the mitochondrial apoptosis-induced channel and cause cytochrome *c* release from mitochondria. To mimic these molecular steps, an OptoBax system consisting of Bax-CRY2 and CIB1-Tom20 tethered to OMM was developed (FIGURE 8*A*). In the light-activated state, CRY2-CIB1 interaction recruited cytosolic Bax toward OMM, while CRY2 self-oligomerization further promoted the clustering of Bax to induce caspase-3 activation and subsequent apoptosis (319).

In addition to apoptosis, necroptosis and pyroptosis constitute two additional programmed cell death pathways associated with inflammation and microbial infection (320–323). Necroptosis is often initiated downstream of death receptors (TNF receptor and Fas) and requires sequential activation of receptor-interacting protein kinase (RIPK)1 and RIPK3 to form necrosome (324–327). Activated RIPK3 phosphorylates mixed-lineage kinase domain-like pseudokinase (MLKL), leading to a conformational change to expose the NH_2_-terminal four-helical bundle domain (4HBD or MLKL-N). Subsequent MLKL oligomerization and PM translocation mediated by inositol phosphate (IP) kinase lead to PM pore formation, PM rupture, and necroptosis (321). To achieve light-modulated necroptosis, RIPK3 was fused to CRY2-PHR and underwent oligomerization upon photoillumination. An increase of phosphorylation of MLKL and coclustering of oligomerized RIPK3 and MLKL were observed. Similarly, CRY2-mediated oligomerization of a MLKL-N mutant (H15A/K16A/R17A), named light-induced nonapoptotic tool 1 (LiPOP1; FIGURE 8*B*), was devised to control its binding to PM-resident phosphoinositides and elicit PM rupture-induced cell death with a photoactivation half-life of 18 min. LiPOP1 was further coupled with NanoLuc-mediated bioluminescence to induce tumor cell necroptosis in living animals within 30 min. NanoLuc could convert furimazine (Fz) into furimamide, accompanied by photon emission at ∼460 nm to activate CRY2-based LiPOP1 (24). In a second approach, MLKL-N was fused to the NH_2_ terminus of cpLOV2 to block its PM-perforating activity in the dark. Light irradiation led to the exposure of MLKL-N to restore its necroptosis-inducing function (23).

Pyroptosis is another form of immunogenic cell death triggered by pathogen invasion (323, 328, 329). LPS stimulation or bacterial toxin leads to inflammasome formation and activation of caspase-4 and -5 in humans and the mice ortholog caspase-11 or caspase-1 found in both humans and mice, resulting in the cleavage of gasdermin D (GSDMD). The cleaved GSDMD NH_2_ terminus (GSDMD-NT) forms pores in the PM to induce pyroptosis (321). This physiological process could be faithfully reconstructed through optogenetic engineering of GSDMD as the effector of pyroptosis. In a second LiPOP tool (LiPOP2a; FIGURE 8*C*), LOVTRAP was used to control the disassembly of a split GSDMD (CT + NT) with light, thus mimicking caspase-mediated proteolytic cleavage. Photostimulation led to dissociation between GSDMD-NT and GSDMD-CT, with GSDMD-NT immediately resuming its activity to induce pyroptosis within hours (24). In a second design (LiPOP2b; FIGURE 8*C*), the autoinhibitory GSDMD-CT was replaced by cpLOV2, with the latter capable of masking the active site of GSDMD-NT fused to its NH_2_ terminus when shielded from light. Exposure to blue light was found to trigger pyroptotic cell death with an estimated half-life of 3–6 h (24). Ideally, LiPOP can be repurposed as a suicide device or a safety brake to eliminate adoptively transferred cells during transplantation or CAR-T cell therapy. Moreover, these tools can be used to precisely elicit cytokine release and proinflammatory response in tumor microenvironment, thereby illuminating how controllable necroptosis or pyroptosis can be harnessed to benefit cancer immunotherapy.

### 4.9. Intrabody-Antigen Recognition

Owing to their cysteineless backbone, small size (10–15 kDa), and ease of production, intracellular antibodies (intrabodies), such as nanobodies and monobodies, have been gaining wide applications in structural biology, cell biology, cancer diagnostics, and therapeutics (330–336). Nanobody, naturally produced in camelids (330), contains a small single variable domain on a heavy chain (VHH) with three complementarity-determining regions (CDRs) as the antigen-recognition module. By comparison, monobody is a non-IgG protein binder derived from the fibronectin type III domain and contains three distinct loops (BC, DE, and FG loops) as CDR equivalents to form the antigen-binding regions (337, 338). Recently, photosensitive domains have been engineered into both nanobody and monobody to produce photoswitchable intrabodies, named Sunbody (or optoNB) and Moonbody (or optoMB), respectively (339–341).

**FIGURE 9.**
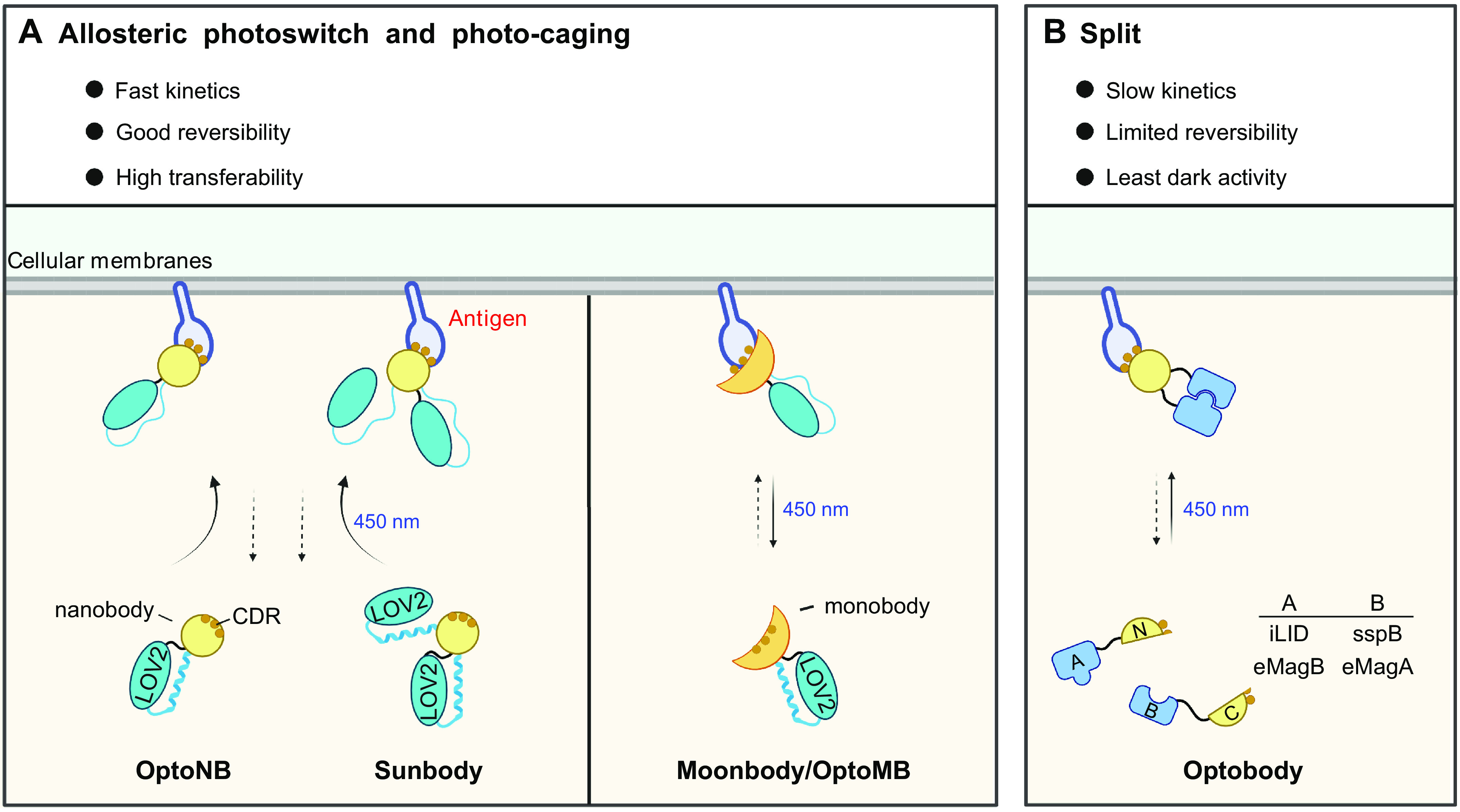
Design of photoswitchable intrabodies. *A*: engineering of light-switchable nanobody (Sunbody or OptoNB) and monobody (Moonbody or OptoMB) via photoswitch insertion or fusion. LOV2 was inserted into exposed loops of nanobodies or monobodies to confer photosensitivity. The fusion of a second copy of LOV2 to the NH_2_ terminus of a nanobody further improves the photoresponsiveness of Sunbody. *B*: optobody engineered from a split nanobody. Optical dimerizers (iLID or eMags pairs) are fused to the NH_2_- or COOH-terminal parts of a split nanobody, respectively. Light stimulation leads to the reassembly of a functional nanobody to restore its antigen recognition capability. See glossary for abbreviations. Image created with BioRender.com and used with permission.

The first approach takes advantage of LOV2 as an allosteric photoswitch to engineer light sensitivity into intrabodies, thereby enabling light-inducible antigen-antibody association or dissociation (FIGURE 9*A*). For instance, LOV2 was inserted into a non-CDR loop next to CDR1 and CDR2 of LaM, a nanobody that specifically recognizes mCherry, to generate optoNB (340) and Sunbody (341) in two independent studies. The fusion of one additional LOV2 to the NH_2_ terminus of LaM further enhanced the photodynamic performance by increasing the range of light-induced changes in antibody-antigen binding (341). When anchoring mCherry as the antigen toward different intracellular organellar membranes (PM, mitochondria, ER, nuclear envelope, or early endosome), light-triggered translocation of Sunbody could be used to study location-specific protein functions. Sunbody was also coupled with the FLARE system or a cytosine base editor to enable light-inducible transcriptional activation or base editing in mammalian cells (341). For OptoNB, LOV2 insertion into the non-CDR loop 1 between Gly15 and Gly16 (LaM-GG15) caused light-inducible dissociation of LaM from membrane-bound mCherry. By contrast, insertion to loop 6 between residues Ala74 and Lys75 (LaM-AK74) led to light-inducible LaM-mCherry interaction (340). OptoNB was coupled with SOScat to module the RAS-ERK pathway. In cells expressing membrane-bound mCherry (CAAX-mCherry), OptoNB-fused SOScat underwent light-dependent recruitment (with LaM-AK74) or undocking (with LAM-GG15) from PM-anchored mCherry, thereby leading to photoinducible activation or inactivation of ERK signaling, respectively (340).

Similarly, Moonbody and optoMB were independently designed by inserting LOV2 into the EF loop (residues Pro73 and Gly74) (341) or between residues Ser58 and Ser59 (339), respectively, to a monobody that specifically recognizes the Src homology 2 (SH2) domain of Abelson tyrosine kinase (Abl). Both engineered monobodies showed light-induced dissociation from the SH2 antigen. Moonbody showed reversible interaction with its binding target, with a dissociable half-life of ∼8 s and an associable half-life of ∼50 s. Moonbody was further used to control the degradation of its binding target in a light-dependent fashion (341). Similar engineering approaches were extended to develop moonbodies against the small ubiquitin-like modifier protein (SUMO) and the maltose-binding protein as a commonly used fusion tag for protein purification. Such photoswitchable monobodies could be used for cost-effective protein purification (339) or enrichment of SUMOylated proteins with light (341).

The second approach involves the use of an optical dimerizer to reassemble split nanobodies by light. A series of optogenetically activatable intracellular antibodies (optobodies; FIGURE 9*B*) have been created by splitting nanobody into two parts on the opposite sides of CDRs and conjugating each part with the Magnets or iLID optical dimerizers (342). For the anti-GFP nanobody, the most ideal split site was identified between Asn65 and Cys66. The split nanobody showed no interaction with its target in the dark but gradually restored its interaction with GFP within 1 h. With this strategy, optobodies against endogenous targets, such as β2-adrenergic receptor (β2AR) and the actin-binding protein gelsolin, were developed for light-inducible perturbation of β2AR signaling and actin cytoskeleton dynamics. The approach could be further extended to engineer light-activatable split single-chain variable fragment (ScFv). A GCN4-specific optobody was generated by splitting the VH segment but not the VL region of scFV (342). Compared with the LOV2 insertion method, the split intrabody approach has the least dark activity but suffers from slow kinetics and limited reversibility.

### 4.10. Protein Degradation

**FIGURE 10.**
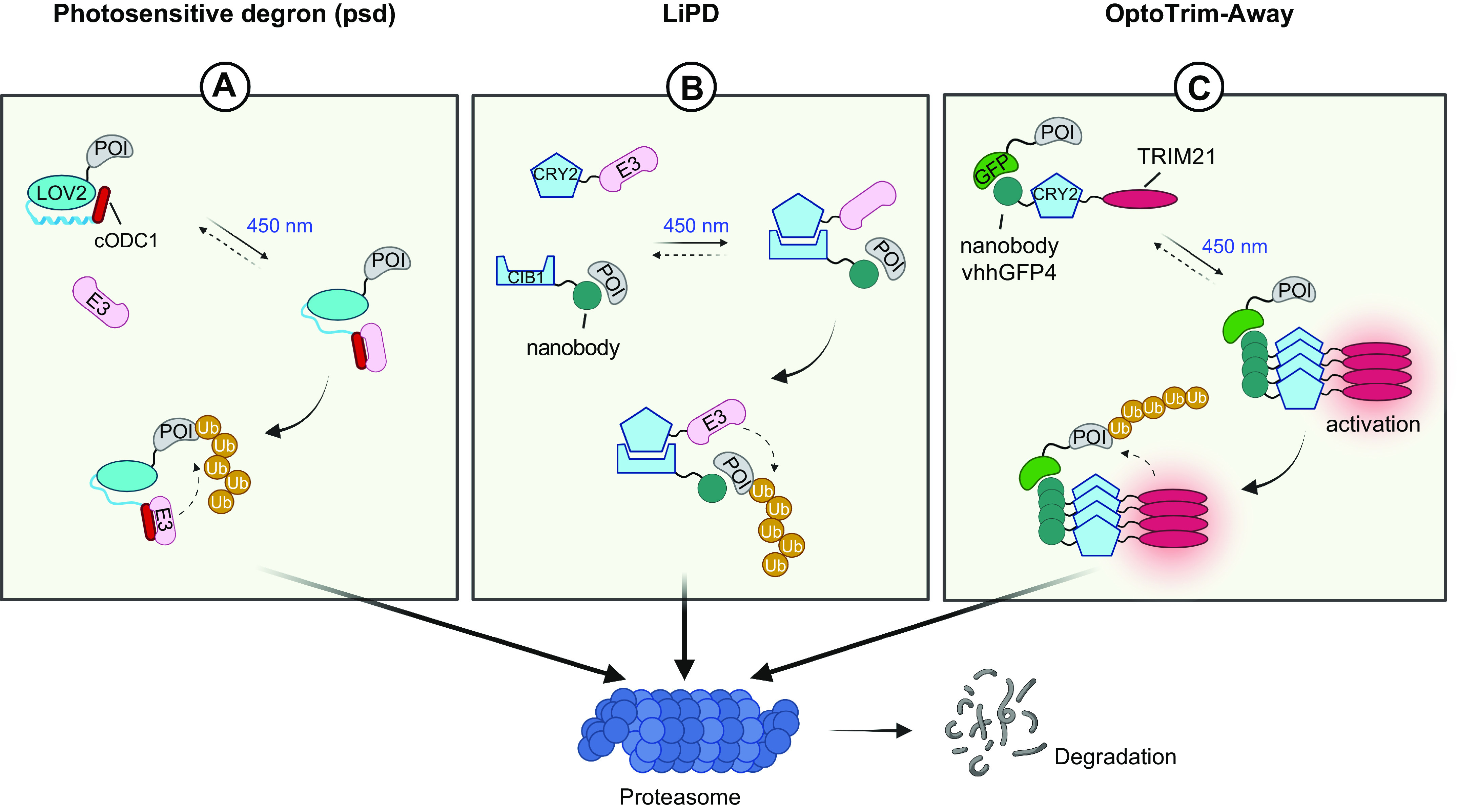
Light-inducible protein degradation systems. *A*: photosensitive degron. LOV2 is used to mask the COOH-terminal degron sequence from the murine ornithine decarboxylase (ODC). Blue light illumination uncages cODC1 to recruit E3 ligase for protein ubiquitination and proteasomal degradation. *B*: LiPD, consisting of a CRY2-fused E3 ligase and a nanobody conjugated with CIB1, engages and ubiquitinates the CIB1-nanobody-antigen/POI complex for proteasomal degradation. *C*: OptoTrim-Away. The TRIM21 E3 ligase is fused with CRY2 and an anti-GFP nanobody (vhhGFP4) to engage GFP-tagged POIs. Light stimulation induces TRIM21 clustering and activation, thereby resulting in POI ubiquitination and subsequent degradation. See glossary for abbreviations. Image created with BioRender.com and used with permission.

Existing light-inducible protein degradation systems are mostly made by taking advantage of degrons or bringing a POI into close proximity to the proteasomal degradation machinery. For example, a photosensitive degron (psd) was generated by combining LOV2 with a COOH-terminal synthetic degradation sequence (MSCAQESITSLYKKAGSENLYFQ) derived from the COOH- terminal degron of murine ornithine decarboxylase (cODC1) (FIGURE 10*A*). The cODC1 psd module was fused to cell cycle regulators to control their degradation and modulate yeast growth (343).

In a second case, a two-component system was used to design a light-induced protein depletion (LiPD; FIGURE 10*B*) tool (344). LiPD consists of CRY2 fused to a nanobody as the target binding module and CIBN conjugated to an E3 ligase RING domain, thereby allowing the conditional recruitment of a POI for targeted ubiquitination upon blue light stimulation. LiPD was shown to cause target protein depletion by 80% within 4 h. With a similar design, moonbody has also been repurposed for conditional protein degradation. To achieve this, an anti-SH2 moonbody was fused to the auxin signaling F-box 2 protein (AFB2) of the Skp1-Cul-F-Box E3 ligase complex. Once expressed, the AFB2-moonbody fusion protein led to proteasomal degradation of an SH2-tagged target protein in the dark by bringing the complex toward the E3 ligase complex. Upon light illumination, AFB2-moonbody dissociated from its target and hence prevented SH2 from degradation (341). This method can be adopted as an alternative to the auxin-inducible degron degradation system (345).

Independently, a self-ubiquitinated E3 ligase tripartite motif 21 (TRIM21) has also been engineered to induce protein degradation with blue light. TRIM21 binds to the Fc domain of an antibody and causes degradation of antibody-bound pathogens through the ubiquitin-proteasome pathway (346–348). Target-induced clustering of TRIM21 through its RING domain activates its ubiquitination activity for selective degradation of antibody-bound targets. An OptoTrim-Away construct, which contains TRIM21 fused with CRY2 and an anti-GFP nanobody vhhGFP4, has been generated to demonstrate light-inducible degradation of GFP-tagged protein (FIGURE 10*C*). Given that the RING domain alone is sufficient to trigger clustering and proteasomal degradation, a minimal version of OptoTrim-Away was created to cause target degradation to a similar level of full-length TRIM21 (349). Red or far-red light-inducible degradation systems are yet to be developed to enable paralleled degradation of multiple endogenous targets in the same cells.

### 4.11. Genome Engineering and Transcriptional Regulation

**FIGURE 11.**
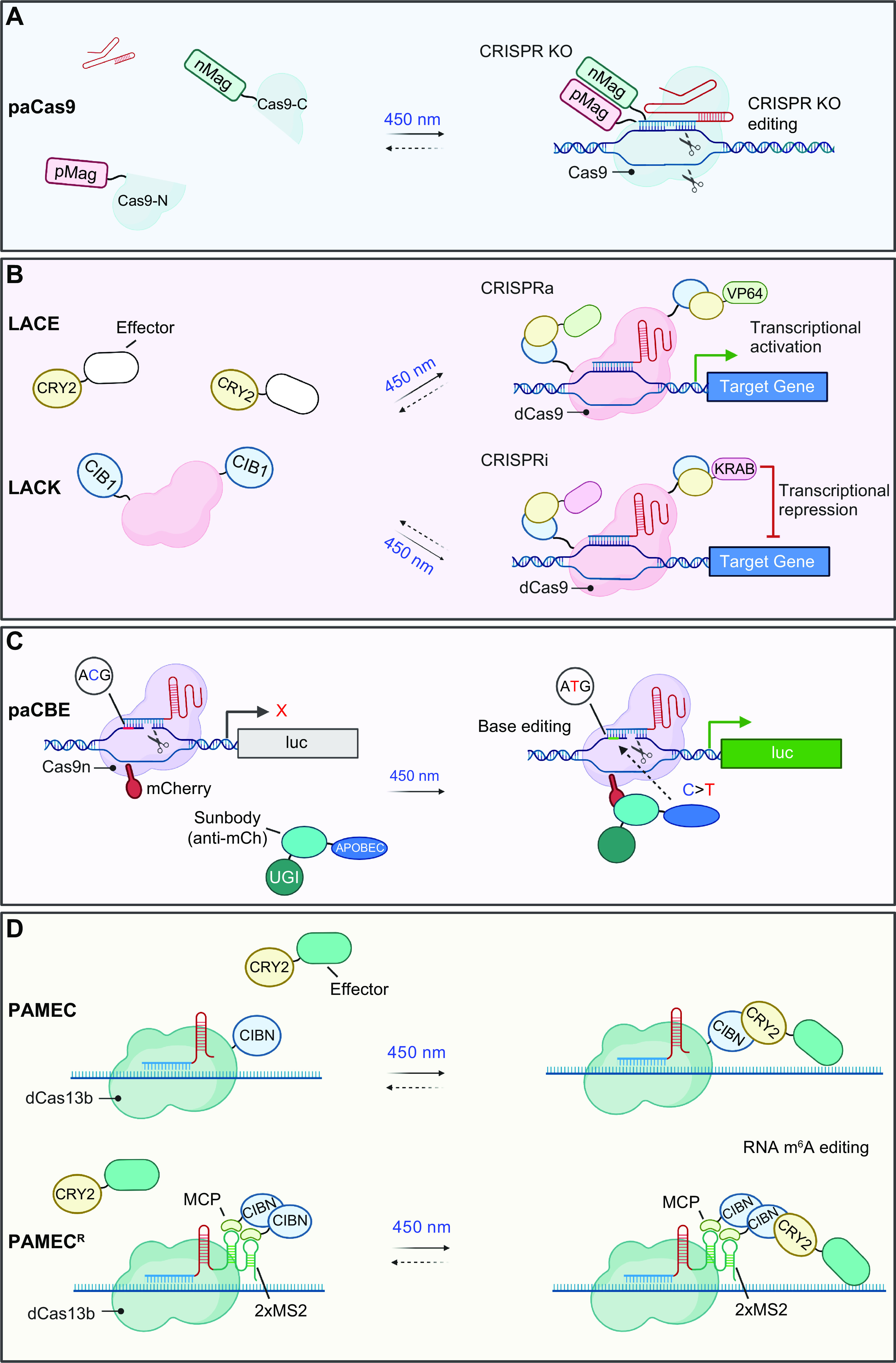
Optogenetic genome engineering and transcriptional regulation. *A*: photoactivatable Cas9 (paCas9). The NH_2_-/COOH-terminal parts of Cas9 are fused to nMag and pMag, respectively. Light illumination reassembles an intact Cas9 to restore its genome editing function in the presence of sgRNA. *B*: photoactivatable CRISPRa (LACE) or CRISPRi (LACK) systems. Based on the CRY2-CIB1 optical dimerizer, light is utilized to recruit a transcription activator (VP64) or repressor (KRAB) to endogenous genomic loci bound by dCas9/sgRNA, thereby enabling photocontrollable transcriptional reprogramming. *C*: paCBE is a photoactivatable cytosine base editor that consists of an anti-mCherry Sunbody-fused APOBEC1 and uracil DNA glycosylase inhibitor (UGI) that interacts with the mCherry-tagged Cas9 nickase to induce sgRNA-targeted C-to-T conversion. paCBE can be used to initiate the transcription of target or reporter (such as luciferase or luc) genes. *D*: PAMEC mediates RNA m6A editing by light using CIBN-fused Cas13b and CRY2 appended to m6A modulators (such as RNA demethylase FTO or the methyltransferase domains of METTL3/4). PAMEC^R^ further increases m6A editing efficiency by adding MS2 aptamer hairpins to the 3′ end of Cas13b crRNA for additional anchoring of MS2 coat protein (MCP)-fused CIBN to recruit more CRY2-fused effectors. See glossary for abbreviations. Image created with BioRender.com and used with permission.

Photoactivatable genome editing and engineering tools have been developed by using optical dimerization modules to reconstitute the activity of CRISPR/Cas9 and TALE in the presence of light. A photoactivatable Cas9 (paCas9; FIGURE 11*A*) was constructed by splitting Cas9 into two nonfunctional fragments and fusing each with pMag and nMag, respectively (350). Blue light irradiation induced pMag-nMag interaction, which restored Cas9 function to mediate genome editing and transcriptional activation. To conditionally inhibit the CRISPR-Cas9 activity, CASANOVA as an optogenetic anti-CRISPR system was engineered by inserting LOV2 into a phage-derived anti-*Streptococcus pyogenes* Cas9 AcrII4 protein (129). Another tool, termed LACE for “light-activated CRISPR-Cas9 effector” (FIGURE 11*B*), utilized the CRY2-CIB1 pair, dCas9, and VP64 to induce transcriptional activation (146). CRISPR interference (CRISPRi) allows sequence-specific suppression of gene transcription (351). A recent study suggests that the fusion of a Krüppel-associated box (KRAB) repressor domain to dCas9 outperforms the original CRISPRi system (352). By replacing the transactivator VP64 in the LACE system with KRAB, a light-activated CRISPRi using KRAB domain (LACK) tool has been developed to enable light-dependent transcriptional repression (FIGURE 11*B*).

Likewise, light-inducible transcriptional effectors (LITEs) were devised by combining the CRY2-CIB1 heterodimerization pair with TALE modules designed to bind customizable DNA sequences and a transcriptional activator VP64 for target gene expression (143). Moreover, locus-specific epigenetic mark-modifying TALE-histone effector fusion constructs (epiTALE) were developed by fusing CIB1 with TALE and CRY2-PHR with epigenetic modifiers, including histone methyltransferase and histone deacetylases.pdDronpa1 was also employed to design a photoswitchable transcriptional reprogramming tool based on a catalytically inactive SpCas9 (dSpCas9) and a hybrid VP64-p65-Rta (VPR) tripartite transactivator (353). Dimerization of pdDronpa1 in the dark prevented dCas9-DNA interaction to abrogate transcriptional activation by VPR. Light illumination at 500 nm led to pdDronpa1 dissociation to restore dSpCas9-target interaction to turn on gene transcription. Light-inducible gene transcription was also made possible by using a LightOn system, which contains VVD and DNA-recognition domain of Gal4(1–65). Blue light stimulates Gal4(1–65)-VVD dimerization and subsequent binding to the upstream activating sequence of Gal (UASG) to switch on gene expression. With an optimized VVD dimerization-enhancing mutant (N56K and C71V), LightOn was used for light-switchable transgene expression in mammalian cells and in the liver of mice (354).

Building upon the FLARE system (315), Sunbody has been repurposed to generate SolarFLARE for light-tunable gene transcription. In the SolarFLARE design, the Ca^2+^-sensing calmodulin-TEV chimera was replaced with an anti-mCherry Sunbody-TEV fusion protein and the PM-bound CaM-binding MKII peptide with an mCherry to become PM-mCh-LOV2-TEVcs-tetR-VP16. Light-triggered Sunbody-mCherry interaction brings TEV into the proximity of TEVcs for cleavage, resulting in the release of transcriptional coactivator tetR-VP16 that subsequently binds the tetracycline operator (tetO) sequence and confers light control over gene transcription. The application of Sunbody was further extended to CRISPR/Cas9-mediated C-to-T base editing (355) to create a photoactivatable cytosine base editor (paCBE; FIGURE 11*C*). paCBE contains two parts: an mCherry-tagged Cas9 nickase with sgRNA and an anti-mCh Sunbody fused with cytidine deaminase APOBEC1 and a uracil DNA glycosylase inhibitor (UGI) to inhibit U:G mismatch repair. Blue light irradiation induced the interaction of two parts of paCBE to initiate C-to-T editing in the genome (341).

Optogenetic engineering has been further extended to control RNA modifications in mammalian cells. A photoactivatable RNA N^6^-methyladenosine (m6A) editing system, designated PAMEC (FIGURE 11*D*), was developed by using type VI RNA-guide RNA-targeting CRISPR-Cas13b from *Porphyromonas gulae* (356). Using CIBN-fused dCas13b and CRY2PHR appended to fat mass and obesity-associated (FTO) protein or methyltransferase domains of METTL3 and METTL4, PAMEC enabled blue light-dependent m6A erasure or installation in the presence of a guide RNA. To further increase the m6A editing capability, PAMEC^R^ was developed by adding two MS2 aptamer hairpins to the 3′ end of Cas13b crRNA, thereby generating scaffold RNA for additional anchoring of MS2 coat protein (MCP)-bound CIBN (FIGURE 11*D*). These photoswitchable nucleic acid-modifying tools will likely have broad applicability for precise editing and engineering of mammalian genome, epigenome, and epitranscriptome, as well as for mechanistic dissection of causal effects among genotype, epigenotype, and phenotype.

### 4.12. Cell-Cell Communications

**FIGURE 12.**
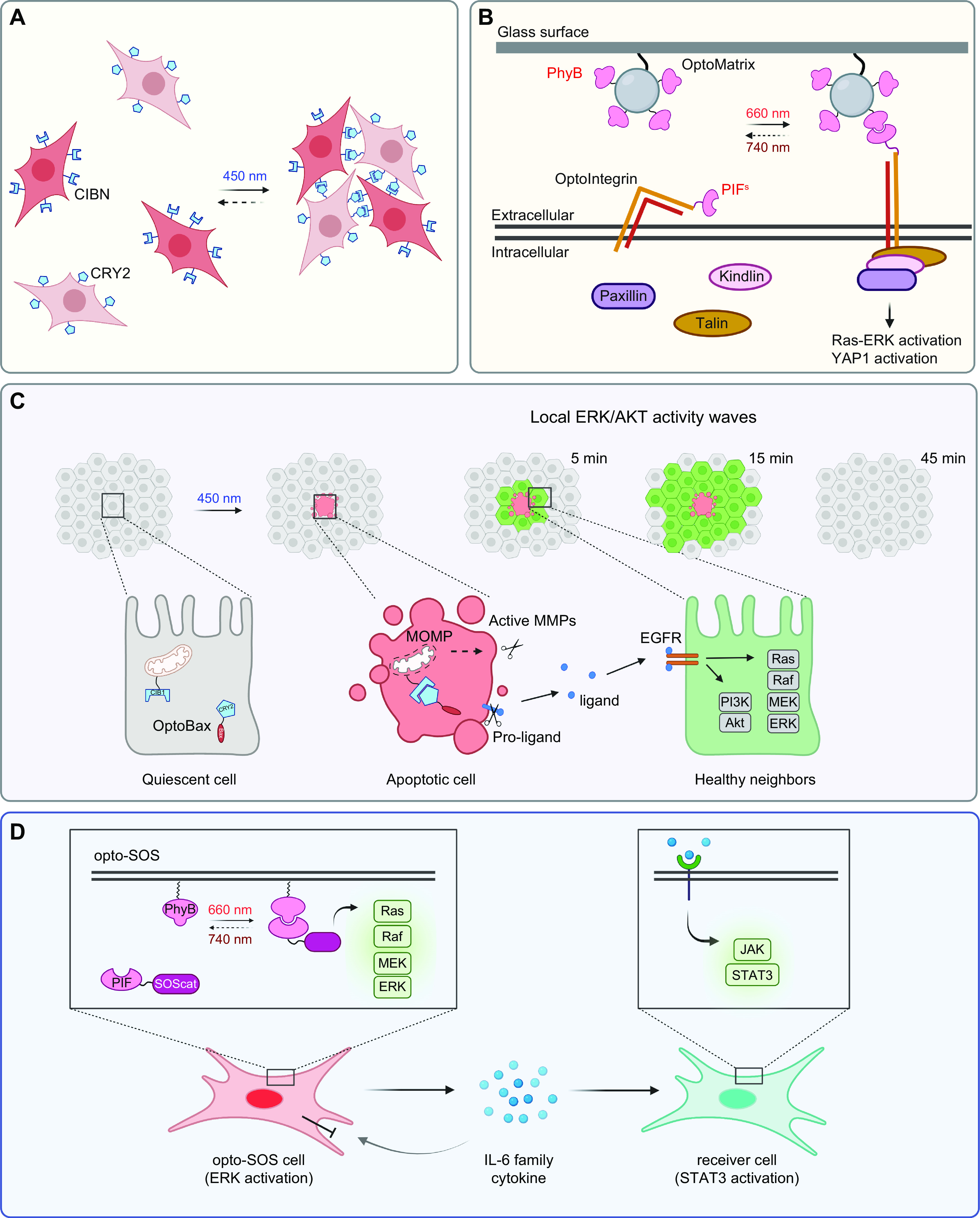
Optogenetic regulation of cell-cell communications. *A*: self-assembly of MDA-MB-231 breast cancer cells can be achieved by using a CRY2-CIBN photodimerization pair separately expressed on the cell membrane. Blue light stimulation induces MDA-MB-231 cell cluster formation. *B*: optogenetic regulation of integrin interaction with the extracellular matrix (ECM). Under 660-nm light, OptoIntegrin (PIF6-fused to the extracellular domain of the β-subunit of integrin α_V_β_3_) interacts with glass-coated OptoMatrix (glass surface-bound PhyB) to trigger integrin-ECM engagement-induced RAS-ERK activation and mechanosensory YAP1 activation. *C*: OptoBax-induced cell apoptosis upon exposure to blue light by translocating CRY2-BAX to interact with mitochondrion-anchored CIB to trigger mitochondrial outer membrane permeabilization (MOMP) and cell death. The resulting active matrix metalloproteinases (MMPs) cleave pro-EGF ligands on the apoptotic cells, which then triggers an ERK/Akt wave to support cell survival over hours through EGFR signaling in the healthy neighbors that could last for 5–15 min. *D*: activation of Ras-ERK signaling in Opto-SOS (PIF-SOScat binds to membrane-targeted PhyB upon 660-nm illumination)-expressing sender cells triggers a paracrine pathway by secreting STAT3-activating factors, such as IL-6, while simultaneously inhibiting autocrine pathways to prevent self-activation of STAT3. See glossary for abbreviations. Image created with BioRender.com and used with permission.

Tissue physiology is constituted by intricate cell-cell interaction networks. Interactions between cell surfaces in proximity or long range (sender-receiver) are mediated by ions, integrins and extracellular matrix, metabolites, junction proteins, and ligand-receptor pairs that involve growth factors, cytokines, neurotransmitters, and chemokines. Signaling propagation from cell to cell induces changes in gene expression and proteomic profiles. Cell-cell interactions among single cells or a collective cell population play crucial roles in cell migration, organ tissue development, and disease progression (357). Given the spatiotemporal precision and single-cell resolution of light stimulation, optogenetic tools have been used to precisely probe signaling and manipulate cell-cell interactions. To modulate direct cell-cell interaction through a light-controllable system, the CRY2-CIBN photodimerization pair was expressed in the MDA-MB-231 breast cancer cell line (FIGURE 12*A*), known to express low levels of cadherins. CRY2 or CIBN was cloned into a pDisplay vector in frame with the NH_2_-terminal Ig κ-chain secretion signal sequence and COOH-terminal transmembrane domain of the platelet-derived growth factor receptor (PDGFR) (FIGURE 12*A*). Light stimulation was applied to induce cell cluster formation and tissue engineering (358).

To enable optogenetic regulation of integrin interaction with extracellular matrix (ECM), a secretion-optimized PIF6 was inserted into the extracellular domain of the β-subunit of integrin α_V_β_3_ to generate OptoIntegrin (FIGURE 12*B*), which could interact with PhyB under 660-nm red light stimulation. Subsequently, light-sensitive OptoMatrix was made by coating slides with (3-glycidyloxypropyl)trimethoxysilane and neutravidin (FIGURE 12*B*), followed by addition of biotinylated PhyB that binds neutravidin. Under 660-nm light, cells expressing OptoIntegrin tightly adhered to the OptoMatrix and triggered integrin-ECM engagement-induced RAS-ERK activation and mechanosensory YAP1 activation (FIGURE 12*B*). This process could be reversed by exposing OptoMatrix to 740-nm light (359).

Tissue level cell turnover and survival are governed by waves of extracellular (nonautonomous) signal-induced ERK and AKT activity pulses to healthy surrounding cells, which can be triggered by cell apoptosis through EGFR and metalloproteinase (MMP) signaling (360). To mimic this physiological process, OptoBax (319) was used to trigger cell apoptosis at the single-cell level within 1 h (FIGURE 12*C*). Resultant ERK and AKT waves were observed, which was found to be identical to starvation-induced apoptosis but independent of caspase activation. Furthermore, OptoFGFR (233) and OptoRAF (361) were used to induce protective ERK and AKT waves (FIGURE 12*C*) (360). Coculture of sender cells expressing an optogenetic construct (such as Opto-SOS) with wild-type cells (receiver cells) provides a controllable system to study dose- and intensity-dependent signaling outputs from sender cells to receiver cells. In the case of Opto-SOS-stimulated Ras-ERK signaling (FIGURE 12*D*), a sustained signaling activation is required within the sender cells to trigger a paracrine pathway by secreting STAT3-activating factors such as interleukin-6 (IL-6) while simultaneously inhibiting autocrine pathways to prevent self-activation of STAT3 (234).

## 5. PHYSIOLOGICAL MODULATION IN VIVO BY WIRELESS OPTOGENETICS

To push optogenetics forward toward real-world applications in living animals and ultimately in humans, notable bottlenecks have to be tackled, which include *1*) the efficient delivery of the genetically encoded materials to targeted regions, *2*) the limited depth of tissue penetration associated with blue and green light-activatable optogenetic constructs, and *3*) the inability to extend the spectral sensitivity of optogenetic constructs into the tissue transmissible range to reach body-circulating cells or deeply buried tissues. The requirement of a large head-mounted device or implantation can induce tissue damage and irritation, which greatly hampers the in vivo application of optogenetics in freely moving animals. The emerging concept of “wireless optogenetics” (20, 362–367) aims at solving these hurdles and could be defined as a noninvasive or minimally invasive, precisely and remotely light-controlling platform for the studies of tissue biology, behavior, and diseases in freely moving animals or primates. The advances in photonics, material engineering, and nanofabrication technologies used in the semiconductor industry have been adapted for the application of wireless optogenetics (TABLE 3). The most recently developed wireless optogenetic platform exists as a closed-loop optofluidic system that enables cell type-specific delivery of genetically encoded photosensitive switch and fluorescent probes with high spatial selectivity (367). Adeno-associated virus (AAV)-based viral transduction systems combined with the Cre-loxP genome engineering method can achieve efficient and cell type-specific gene silencing (368) and expression of light-activated genes (369). In a second example, a deep-tissue implantable, miniaturized, wireless-powered, multifunctional device has been invented to integrate simultaneous optical stimulation and signal recording. Transdermal light illumination or optical fiber implantation has been applied to reach shallow tissues. Sophisticated but simplified light delivery systems beyond optical fibers have also been invented to control the behavior of live mice, as exemplified by cellular-scale μLED implants. Such miniature μLEDs allow precise control over the property of light pulses (frequency, intensity, and photocycle) because they can be wirelessly controlled by battery-powered infrared (IR) or head-mounted radiofrequency (220, 363, 370, 371). In addition, transparent tissue interfaces and materials are developed for localized optogenetics and simultaneous electrophysiological recording. These include transparent conducting oxides, carbon-layered electrode array of graphene, metal nanowires, and conductive polymers (367, 372). Such devices can be further coupled with programmable drug delivery channels to incorporate pharmacological agents (363, 373). Drugs, in the form of small molecules, ligands, or microbial products, can be pumped at programmable flow rates through microfluidic channels fabricated with thin and flexible silicon shanks or polydimethylsiloxane (PDMS). Notably, the recent discovery of red-shifted photoreceptors, the invention of NIR light-responsive nanoparticles, and the combination of optogenetics with magnetic stimulation will likely provide additional solutions to this long-standing challenge in the field (17, 22, 24, 26, 374, 375).

**Table 3.** Summary of devices used in wireless optogenetics and selected in vivo applications

Device/Light Source	Mechanism	Substrate	Wavelength (nm)/Output	Advantages	Disadvantages	Applications
μLED	Radiofrequency (RF)		450	Minimally invasive implant integrated with drug delivery systems	RF has a short operation range, decays over a few days, and remains susceptible to signal interference and absorption by tissue; repeated and sustained drug delivery is still challenging.	Brain (362); spinal cord (364); peripheral neurons (364)
μLED	Battery-powered infrared light (IR; 950 nm)		400–700 or any optical wavelength	Minimally invasive, combined with ultrathin, soft microfluidic drug delivery, allowing for simultaneous photostimulation and pharmacological manipulation; closed-loop design	Requiring thermally stable compounds; battery-powered and IR receiver system is needed; nondynamic and nonrefillable microfluidic flow; line-of-sight handicap; thermal-mechanical micropump generates heat; complexity in system design makes it not readily accessible	Brain (363)
μLED	Smartphone Bluetooth low energy (BLE)		470, 589 or any optical wavelength	Replaceable drug cartridges for chronic pharmacology and optogenetics; ultrathin, soft multimodal drug delivery system with dual wavelengths of light; long-range coverage (10–100 m), no line-of-sight handicap	Unadjustable volume and rate of drug infusion; requires drugs or chemicals to be thermally stable; head-mounted devices are invasive and potentially induce inflammation; requires battery recharging.	Brain (373); spinal cord (365); peripheral neurons in bladder (20)
μLED	Battery-free magnetically coupled antennas; near-field communication		Any optical wavelength	Closed-loop control of photostimulation with recording and data processing capability; refillable and reusable; ultrathin, soft multimodal drug delivery system of multiple types of drugs and photostimulation; lightweight and compact; wireless and battery-free, continuously powered by near-field communication (NFC) technology	Head-mounted devices are invasive and potentially induce inflammation.	Brain (366); spinal cord (431, 432); Peripheral nerve (388)
Deep-tissue optical focusing	Guide star-based wavefront shaping (feedback or optical phase conjugation; e.g. ultrasonically encoded guide stars, TRUE, AOTM)		532	Achieves diffraction-limited control of light beyond 1 cm within a scattering material; combines optical phase conjugation with the ultrasound guide star to enable light focusing at depths and noninvasive optical imaging and optogenetics.	An internal guide star is embedded at the targeted location that often requires invasive positioning and fixes light delivery to a single location; accurate focusing requires millions of unique measurements; inefficiencies and residual heating are current limitations.	TRUE (389, 390); TROVE (433); AOTM (393); GePGS in tumor and brain (391, 392)
Deep-tissue optical focusing	Beacon-guided wavefront shaping		532	Enables superresolution optical focusing and optical imaging at unprecedented depth; provides subwavelength guide star feedback inside a complex medium by spin-dependent fluorescence.	An internal guide star is embedded at the targeted location that often requires invasive positioning and fixes light delivery to a single location.	Superresolution optical focusing using quantum reference beacon (394)
Bioluminescence	*Gaussia* luciferase	Coelenterazine (CTZ)	480	Brighter versions of GLuc generate light sufficient to activate a nearby opsin; noninvasive, no unintended damage; GLuc variants typically emit blue light, emission spectra of luciferases can be engineered or found naturally to cover a wide spectrum of visible light.	Fast light intensity decay, coelenterazine substrate is chemically unstable, and high autoluminescence background, thus requiring luminometers equipped with injectors to measure the transient peak luminescence assay sensitivity.	Brain (396)
Bioluminescence	NanoLuc	Furimazine (Fz) Hydrofurimazine (HFz) Fluorofurimazine (FFz)	456	Brighter bioluminescence with lower background; high physical stability, retaining activity over hours of incubation up to 55°C; improved NanoLuc substrates HFz and FFz are more water soluble, intense, and more prolonged than Fz.	Poor solubility and bioavailability of the NanoLuc substrate Fz; in vivo performance of NanoLuc is poor in deep tissues.	NanoLOGS in tumor (24, 397, 398)
Bioluminescence	teLuc	Diphenylterazine (DTZ)	502	High signal-to-background ratios, small size, minimal cell toxicity; enhanced bioluminescence with DTZ substrate than FLuc-D-luciferin and NanoLuc-Fz pairs in vivo; sustained bioluminescence with 40-min half-life suitable for in vivo application	Poor water solubility	Bioluminescence imaging (399)
Bioluminescence	yeLuc	Selenoterazine (STZ)	527	High signal-to-background ratios, small size; 11.5-fold increase of bioluminescence with STZ substrate over NanoLuc	Poor water solubility; fast decay of yeLuc-STZ emission has ∼5-min half-life; higher toxicity than DTZ	Bioluminescence imaging (399)
Upconversion nanoparticles (UCNPs)	Laser at 980 nm		NIR to visible light	Injectable and minimally invasive nanotechnology-assisted approach for optical control of in vitro and in vivo neuronal activity; absorbs tissue-penetrating NIR light and emits wavelength-specific visible light. Customizable surface coating for cell type-specific targeting and increasing biocompatibility	Biocompatibility, long-term utility and toxicity, dose and parameters of NIR stimulation need to be determined; low upconversion emission and quantum yield with a low-power NIR laser; lack of cell type-specific targeting or uptake of UCNPs; risks of tissue damage or nontargeted thermal neuronal activation caused by NIR irradiation	Brain (374); tumor cell death (24); Ca^2+^ channel (22); dendritic cell-based vaccine (22); CAR-T cell therapy (23, 303)
Gold nanoparticles (AuNPs)	Laser		532 nm to heat	Ligand- or antibody-conjugated AuNPs enable cell type-specific binding to the membrane protein; highly resistant to washout and enables photothermal stimulation with lower delivered energy and resulting heating	Heating effect, clearance or degradation of AuNPs limit the lifetime of cell photosensitivity; repeated treatment of AuNPs is needed; absorption peak at 523 nm and hence less tissue penetrance compared with NIR	Brain (403); CRISPR genome editing (404, 405)
Mechanoluminescent nanoparticles	Ultrasound		470	Activated by deep-tissue focused ultrasound; rechargeable by 400-nm photoexcitation in superficial blood vessels; doping ZnS nanoparticles with Ag^+^ and Co^2+^ stores the photoexcitation energy	Weak intensity of mechanoluminescence from the ZnS:Ag,Co nanoparticles; immediate light emission with a short delay of ∼4 ms is not suitable for photoactivating nonneuronal cells; decay of stored energy over time in the circulation	Brain (407)
Radioluminescent Gd2(WO4)3:Eu nanoparticles	X-ray		610	X-rays freely pass biological barriers; converting X-rays into deep-tissue penetrating red-shifted light	Radiation imposes risk	Brain (408)

See glossary for abbreviations.

### 5.1. Optogenetic Tool-Carrying Viruses

Recombinant viruses, such as AAV, lentivirus (LV), rabies virus (RbV), retrovirus (RV), and herpes simplex virus (HSV), have enabled transgene expression, multicolor labeling, circuit tracing, genome modifications, and perturbation in a cell type-specific manner in vivo (376–378). Among these viruses, AAV has been successfully implemented in many applications demonstrated in the nervous systems of rodents, primates, and even humans. AAVs have been widely used for gene delivery to the nervous system and neural circuit tracing in a Cre-dependent manner. PHP capsid variants of AAV are capable of transducing both central and peripheral nervous systems. For example, PHP.eB and PHP.S are evolved by using a Cre recombinase-based AAV-targeted evolution method based on parent capsid AAV-PHP.B and AAV9 (379). Systematic injection of these engineered AAV variants has been reported to enable noninvasive gene delivery in the central and peripheral nervous systems, such as enteric neurons, dorsal root ganglia, and nodose ganglia (380). Although AAVs infect soma and transport anterogradely, AAV2-retro variants were designed to retrogradely trace neural projections (381). Similarly, canine adenovirus 2 is preferentially taken up by axonal terminals of projection neurons and therefore is useful for interrogating neural circuits in long-range projections. AAV serotypes tailored for nonneuronal cells are also available. For instance, AAV-BR1 is generated for specific targeting of brain endothelial cells to intervene in neurovascular and neurological diseases (382).

RbV has been used for monosynaptic and retrograde tracing to express transgene products such as Ca^2+^ indicators, fluorescent reporters, and microbial opsins. RbV encodes the receptor-binding G membrane protein that helps RbV bind to the receptor on the cell surface for transneuronal transfer. An engineered RbV with G membrane protein deletion (RbV-ΔG) enables monosynaptic circuit tracing (383). By pseudotyping with an avian sarcoma leucosis virus envelope protein (EnvA), RbV-ΔG is incapable of infecting neurons without the coexpression of the EnvA receptor (TVA), thus enabling the tracing of projection neurons expressing TVA. Even though engineered RbV can lead to high expression of transgene products, it also causes cytotoxicity and cell death, thereby preventing the use of RbV for long-term circuit study. Deletion of RbV polymerase has been shown to greatly reduce cytotoxicity to leave the infected cell alive for long-term studies (384). Compared with AAV and RbV, HSV vector is used for rapid transgene expression, as well as anterograde and retrograde transsynaptic tracing with a large genetic payload (385). Long-term HSV (LT-HSV) has also been generated to permit durable gene expression in chronic animal studies (386). For the transduction of nonexcitable cells and tissues, lentivirus and retrovirus are more preferentially used.

### 5.2. μLED

Advancement of bioelectronical engineering and biomaterials has accelerated the applications of optogenetic tools to study cell circuits and signaling transduction in vivo and set the stage for translating optogenetics into therapeutics. Traditional implementation of central nervous system optogenetics requires invasive implantation of optical fibers to the brain that constrains animal movements and remains unreachable to the peripheral nervous system. The injectable microscale inorganic μLED optoelectrode array (362) with minimal stimulation artifact enables precise optical stimulation and temporal recording of neural activities (387). To achieve minimal invasiveness and remote control of optogenetic constructs in vivo, a wireless μLED powered by a resonant radiofrequency (RF) cavity has been implanted to activate neurons in brain, spinal cord, and peripheral nerve of freely moving animals (370). A thin, soft. and stretchable version of such a RF-powered μLED was further developed to improve the biocompatibility (364). In addition, a battery-powered IR-controlled wireless microfluidic device coupled with μLED has been developed for simultaneous delivery of drugs or viruses and photostimulation to the brain in awake and behaving animals (363). However, such a head-mounted device could not be applied for drug delivery and photomodulation in the peripheral nervous system. The microfluidic pump requires high battery capacity, and the unrefillable microfluidic chambers could further limit its use in the clinical setting. To overcome these shortcomings, an optogenetic device with battery-free, ultra-low-power microfluidic using an electrochemical pump was fabricated for photomodulation of the peripheral nervous system (388).

The μLED implant has also been incorporated into a closed-loop setup to treat organ dysfunction in vivo by modulating the nervous system. For example, a closed-loop functional optogenetic stimulation system was used to control ankle joint position by stimulating the peroneal or tibial nerve (371). In rodent bladder-projecting sensory neurons transduced with HSV to express the inhibitory opsin archaerhodopsin 3.0, a closed-loop optogenetic control (CLOC) device was used to record and optically control bladder function in real time. In CLOC, an optoelectronic stimulation and sensing (OESS) module was integrated with μLED wrapping around the bladder. A wireless control and power (WCP) module was installed to record data from the OESS module and control μLED operation, with a Bluetooth tablet-connected data analytic module responsible for communication with WCP. CLOC was demonstrated to modulate bladder sensory afferent neurons to normalize the bladder voiding dysfunction in rodent models (20).

### 5.3. Deep-Tissue Optical Focusing

Optical scattering caused by the heterogeneous optical properties of tissue samples remains an issue that prevents the use of conventional optical systems over 1 mm. Wavefront shaping has the ability to manipulate scattered photons to create a micrometer-scale light focus at depth. The optical technique time-reversed ultrasonically encoded (TRUE) allows light focus at deeply buried regions to monitor and control neural activity in living tissues with a good spatial resolution (389, 390). Guide star was used to provide the location reference for the target inside scattering media for wavefront shaping (391). Successful examples include the genetically encoded photochromic guide star (GePGS) (392) and the acousto-optic transmission matrix (AOTM) guide star (393). Superresolution optical focusing can also be achieved by using quantum reference beacon (QRB) (394). Achromatic metalens further enabled miniaturization of optical focusing devices using wavefront shaping at nanoscales (395). Compared with the most popular optogenetic approaches that rely on invasive implanted optical fibers or μLED, deep-tissue optical focusing enables noninvasive photostimulation with the ability to freely move the focus toward regions of interest at single-cell resolution.

### 5.4. Bioluminescence

Bioluminescence generated during the oxidative reactions of substrates catalyzed by luciferases has been used for bioimaging and as light source for optogenetic stimulation in animal models. It provides an alternative noninvasive means for in vivo optogenetics but at the cost of sacrificing spatial precision.

**FIGURE 13.**
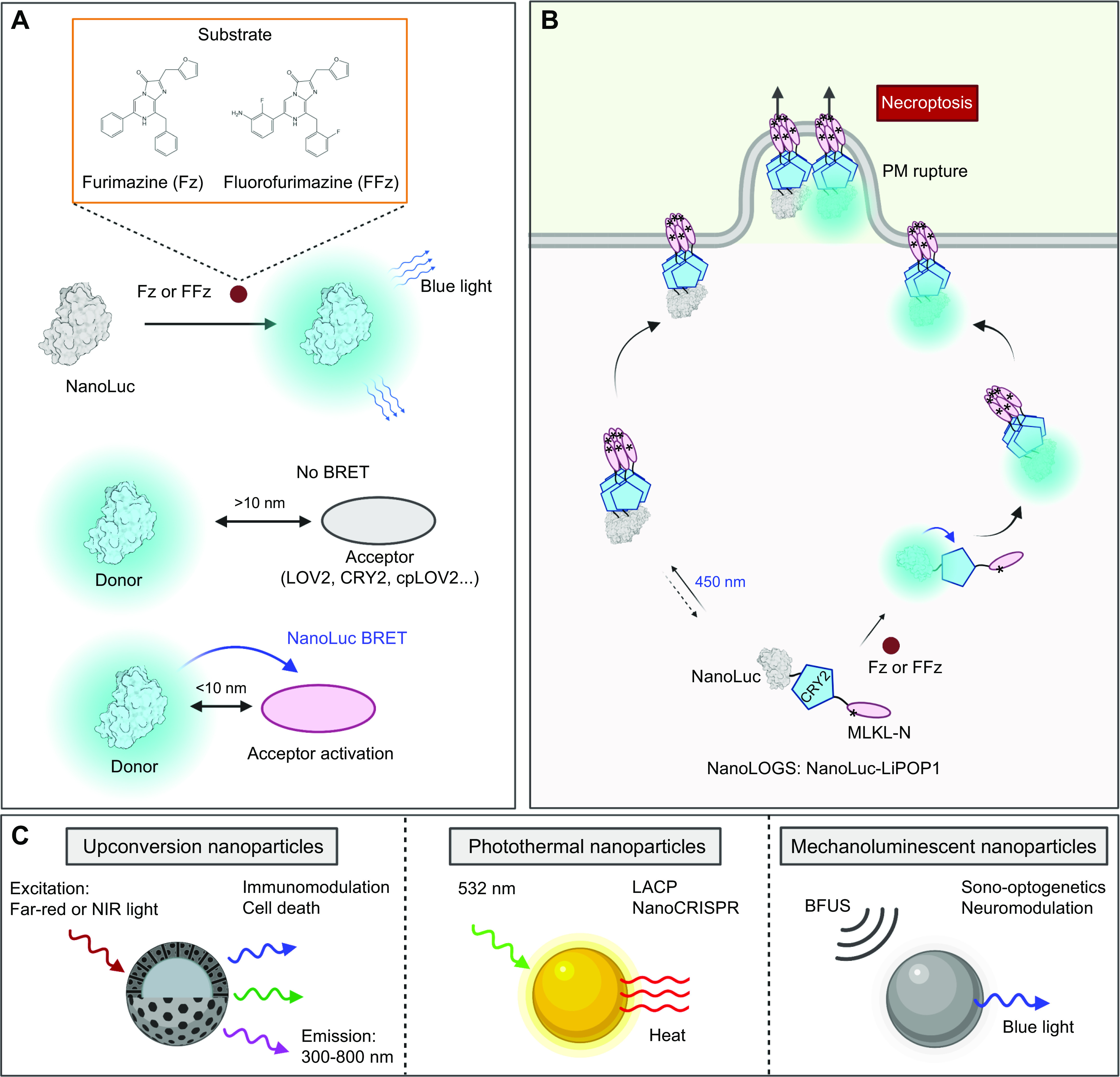
Bioluminescence and nanoparticles exploited for noninvasive wireless optogenetics. *A*: NanoLuc uses furimazine (Fz) or fluorofurimazine (FFz) as substrate to emit bioluminescence in the blue light range. NanoLuc can also serve as a BRET donor to activate blue light-sensitive photoreceptors when an acceptor (e.g., LOV2, CRY2, or cpLOV2) is positioned in close proximity (<10 nm). *B*: NanoLuc-based bioluminescence-aided optogenetic stimulation (NanoLOGS) is applied to enable chemo-optogenetic control of cell death mediated by LiPOP1 in the presence of Fz or FFz. *C*: nano-optogenetic devices enable wireless optogenetics. *1*) Upconversion nanoparticles (UCNPs) are capable of converting far-red or NIR light into visible light in the range of 300–800 nm to activate most existing optogenetic tools. UCNPs have been used in optogenetic immunotherapies and regulation of cell death in living mice. *2*) Photothermal nanoparticles (e.g., AuNPs) convert light irradiation (532 nm) to localized heat. In the LACP or nanoCRISPR design, the generated heat triggers the release of Cas9-sgRNA into cells or HSP70 promoter-driven Cas9 expression for inducible genome editing. *3*) Mechanoluminescent nanoparticles (MPs) convert brain-penetrant focused ultrasound (FUS) to blue light (470 nm), which can be rechargeable by 400-nm light. This sonooptogenetic approach has been used to modulate neuronal activities when combined with ChR2. See glossary for abbreviations. Image created with BioRender.com and used with permission.

Microbial opsin-fused *Gaussia* luciferase (GLuc), designated luminopsin, catalyzes its diffusible substrate coelenterazine (CTZ) into coelenteramide and photon emission to wavelengths similar to blue and green light (396). However, fast light intensity decay, chemical instability, and high autoluminescence background limit its long-term use for optogenetics. By contrast, NanoLuc that catalyzes imidazopyrazinone substrate Fz to emit blue light has brighter bioluminescence (FIGURE 13*A*), improved in vivo environmental tolerance (pH and temperature), and lower background (397). NanoLuc-catalyzed bioluminescence-aided optogenetic stimulation (NanoLOGS; FIGURE 13*B*) was coupled with the CRY2-based LiPOP tools to enable chemo-optogenetic control of necroptosis in mouse tumor models (24). The NanoLOGS method further established fluorofurimazine (FFz) (398) as a substantially improved substrate for chemo-optogenetic applications in vivo (FIGURE 13*B*). Compared with Fz, FFz showed higher aqueous solubility, reduced cytotoxicity, and prolonged bioluminescence and therefore is more suitable for large-scale optogenetic stimulation in chronic experiments.

Along these lines, NanoLuc variants using red-shifted luciferins as substrates can be utilized to improve photonic performance and, more importantly, enable bioluminescence multiplexing. For instance, teLuc with a teal emission peak at 502 nm displayed sustained bioluminescence with diphenylterazine as the substrate, whereas yeLuc with a yellow emission peak at 527 nm exhibited fast turnover with selenoterazine as the substrate (399). These new luciferase-substrate combinations were shown to yield high signal-to-background ratios and elicit minimal cell toxicity for in vivo bioluminescence. Ideally, these red-shifting bioluminescent variants can be coupled with yellow or red light-activatable optogenetic constructs to enable deeper tissue penetration with reduced phototoxicity.

### 5.5. Nano-Optogenetics

Ultrathin μLED implants have minimized invasiveness compared with head-mounted optical fibers but have limited operational time and require surgical implantation. The NanoLOGS approach allows large-scale optogenetic stimulation in deeply buried tissues but requires injection of substrates to sacrifice the spatial resolution (24). A third approach, designated nano-optogenetics (FIGURE 13*C*), combines unique nanomaterials with optogenetic constructs to enable wireless optogenetics. Injectable nanoparticles with good biocompatibility are emerging as an ideal solution to many optogenetic applications in vivo.

#### 5.5.1. Upconversion nanoparticles.

UCNPs (β-NaYF4: Yb, Tm@β-NaYF4) represent a class of lanthanide-doped nanomaterials that are capable of converting deep-tissue-penetrating NIR light into visible or far-red light, ranging from 300 to 800 nm (17, 26, 400, 401), in which Yb^3+^ accepts excitation light and Tm^3+^ serves as the emitter (FIGURE 13*C*). UCNPs can thus activate almost all existing optogenetic constructs. Because the photoactivation window can be shifted to the NIR range, the depth of tissue penetration reaches up to a few centimeters compared with <1 mm with blue light. The UCNP surface can be further functionalized to coat biocompatible silica shell, streptavidin, or antibodies for cell type-specific targeting (22, 303, 402). This approach is most useful when combined with adoptive cell therapies because UCNPs and engineered therapeutic cells can be coinjected to enable wireless optogenetics in living animals. UCNP-based nano-optogenetics has been applied to remotely modulate LiPOP-mediated nonapoptotic cell death, as well as antitumor immune response mediated by engineered dendritic cells and CAR-T cells (FIGURE 13*C*) (22, 23, 303).

#### 5.5.2. Photothermal nanoparticles.

Gold nanoparticles (AuNPs) have been streptavidin coated and conjugated to biotinylated ligands (e.g., neurotoxin Ts1 that targets voltage-gated sodium channels) or antibodies for cell-targeted photothermal stimulation. The attachment of nanoparticles to the cell surface was found to be stable and resistant to being washed out. AuNPs convert 532-nm light into heat that depolarizes the cell membrane by altering membrane capacitance (403). A lipid-capsulated, AuNPs-condensed Cas9-sgRNA plasmid (LACP) has been used to deliver Cas9-sgRNA plasmid (CP) to the cell for genome editing (FIGURE 13*C*). AuNPs converted light irradiation to localized heat, causing the thermal release of CP into the cells (404). Similarly, nanoCRISPR (FIGURE 13*C*), a polymer-coated Au nanorod was shown to convert NIR light to heat, subsequently driving Cas9 expression under a heat-inducible HSP70 promoter (405). Therefore, AuNPs could serve as a multifunctional delivery system when combined with optogenetics for photothermal conversion, cell targeting, and transgene delivery. Notably, compared with direct illumination and heating of tissues with red-shifted light, the ligand-conjugated AuNPs allow cell type-specific targeting and selective heating of a localized environment. Since excess AuNPs can be washed away leaving only neuron-bound AuNPs, strong binding of AuNPs to the target neurons allows temperature changes in the cellular environment to be modest (1.5°C), making it an ideal choice for in vivo applications (403). AuNPs are claimed to be relatively safe because they do not seem to induce changes in cell cycle and apoptosis under certain short-term laser pulses. However, because many physiological processes are temperature sensitive, even modest heating can confound the experimental interpretation (406). Therefore, the photothermal effects following long-term AuNP stimulation are yet to be closely examined (403, 405).

#### 5.5.3. Mechanoluminescent nanoparticles.

Mechanoluminescent nanoparticles (ZnS:Ag,Co@ZnS) triggered by brain-penetrant focused ultrasound (BFUS) can serve as in vivo blue light source (470 nm) for optogenetic stimulation (FIGURE 13*C*), a process named sonooptogenetics (407). Such nanoparticles could be delivered by intravenous administration into the blood circulation for optogenetic neuromodulation and recharged by 400-nm light to store energy.

#### 5.5.4. Radioluminescent nanoparticles.

Radioluminescent Gd_2_(WO_4_)_3_:Eu nanoparticles can downconvert X-ray energy into photons with red-shifted light wavelength around 610 nm (408). Nanoparticles were well dispersed in an aqueous solution via surface modifications with ethylene glycol ligands to enhance the hydrophilicity. Radioluminescent nanoparticles have been successfully applied to transcranially modulate cortical neurons expressing ReaChR, a red-shifted microbial opsin that absorbs light at 590–630 nm.

## 6. ALL-OPTICAL SCREENS

Optogenetics is also emerging as a unique technology to accelerate the pace of drug screening. High-throughput screens built upon pooled libraries for CRISPR-Cas9, base editing, or chemicals have tremendously contributed to the discovery of novel gene function, signaling networks, and pathways of action. In situ sequencing-based optical pooled screens that couple genetic screens with phenotypic screens allow the identification of causal relationships between perturbations and phenotypes (409). However, perturbations in conventional pooled screens are often triggered by ligands or chemicals, which tend to introduce side effects and cross talk among multiple signaling pathways. By contrast, all-optical screens combining optogenetic actuation and recording have enabled programable photoactivation or inhibition by simply varying the intensity, pulse frequency, and duration of light stimulation. The all-optical screen offers high information content but also demands experimental and instrumental setups (e.g., signal-to-noise ratios, programmable plate-based light source, and automation) and applicable computational analysis. An OptoPlate-96 platform has been constructed with controllable illumination for multicolor stimulation in individual wells, as well as a cooling system to prevent overheating-induced toxicity (21). The software program optoConfig-96 written in Python further provided a graphical user interface customized for OptoPlate-96 (410).

Optogenetic actuators can be combined with a growing list of genetically encoded fluorescent reporters or indicators to perturb and monitor diverse aspects of cell physiology in high-throughput assay formats. To avoid optical cross talk between photostimulation and the measurement of optical readouts, red-shifted dyes or reporter proteins that are compatible with optogenetic tools have been commonly used. For example, red genetically encoded calcium indicators were utilized to facilitate dual-color optogenetics (411). In a second case, an all-optical automated system, OptoDyCE (all-optical dynamic cardiac electrophysiology framework), was used by combining ChR2 stimulation with red-shifted dyes for optical sensing of intracellular Ca^2+^ in cardiomyocytes (412). An all-optical screen was also utilized to screen small molecules against protein kinases in a 384-well plate format without the addition of reagents to induce or detect pathway activation and with fewer handling steps. Opto-RTKs (FGFR1, EGFR, ROS1) were used in combination of GFP reporter to detect MAPK/ERK signaling. With the use of this all-optical platform, AV-951 was identified as a ROS1-specific kinase inhibitor (413). This was extended to incorporate a separated color reporter and spatial light stimulation (center vs. peripheral) to capture higher-dimensional information for drug screens in real time. This study clearly shows that the all-optical screen system can be adapted to many drug targets and cellular processes (413).

## 7. LIMITATIONS

Optogenetics has been widely used to control cellular and tissue physiology. However, long-term light delivery to the target cells or tissue regions may cause heating effects, tissue damage, and off-target cellular activities. Sustained light exposure can affect cell viability and trigger transcriptional activation and oxidative stress response in many cell types including immune cells (414, 415), as well as longevity of *C. elegans* (416, 417) and *Drosophila* (418). Visible light (e.g., 390–550 nm, 2.8 mW/cm^2^) can lead to the loss of mitochondrial respiratory activity and damage of mitochondrial DNA (419). Heating (0.2–2°C) resulting from commonly used illumination protocols (30 mW) can change cellular behavior in neurons given their temperature sensitivity (74). This was exemplified by the suppressed spiking activity of medium spiny neurons by light alone (3–15 mW) without expressing opsin (406). Key factors to be considered are wavelength, power intensity, frequency, and scattering nature of light. Optogenetic experiments need to be well designed to include light-only controls. Light exposure also leads to unexpected results in a cell type-specific and time-dependent manner. For example, extended light pulse could depolarize interneurons expressing ChR2 (420) and activate excitatory neurons in a specific layer of brain regions, thereby causing the silencing of neuronal circuits in other layers (421). These caveats should be taken into consideration during data interpretation.

High expression levels and the duration of the expression of given optogenetic proteins may result in dark activity (autoactivation) and undesired cellular toxicity, which may alter cell physiology. In specific brain regions, discrepancy was noted for ChR2 expression driven by different serotypes of AAV vectors compared with its expression by using transgenic mouse models (422). Under such scenarios, it is imperative to assess potential off-target effects and validate key findings with complementary approaches (chemogenetics and electrophysiology, etc.) and the inclusion of control cells or animals. The expression of optogenetic proteins needs to be controlled under minimally required levels and tested across different viral serotype and concentrations if using viral delivery systems. Even though the Cre-LoxP system allows cell type-specific labeling and perturbation, the leaky and insufficient Cre-recombination can result in nonspecific expression. The specificity of the expression of optogenetic proteins should be cross-validated by histological and electrophysiological methods.

In addition, most of the current optogenetic approaches are still invasive. Tethering of animals with optical fiber limits animal behavior and reduces the reproducibility of behavioral test results. Wearable or implantable µLED reduces tissue invasion but still can cause irritation at the tissue-implant interface. Invasive surgery of microdevice implantation can trigger a prolonged inflammation that could subsequently affect cellular activity and confound changes in tissue physiology. Such devices should be monitored for heat generation from the LED and electrical circuits to avoid overheating and local tissue damages. With advanced fabrication technologies, integrated devices with multiple functions have high potential to be reduced in size for less traumatic implantation. However, as the device dimensions are reduced, advanced components and functions are utilized, which may prohibit the wide adoption of sophisticated devices.

Finally, other aspects, including the possibility of light-induced receptor desensitization, internalization, and degradation, warrant further scrutiny in different cellular and animal models.

## 8. CONCLUDING REMARKS

Given the modularity and high transferability of photosensory modules introduced above, the optogenetics toolkit is expanding with an accelerated pace. Continuing efforts are being made to discover and create novel photoreceptors, and to develop better light delivery options for deep-tissue optogenetics. These synthetic biology tools will greatly facilitate the mechanistic dissection of cell signaling and cell physiology. Optogenetic studies starting from mammalian cell culture have now been moving forward to measure cellular or subcellular phenomena and diverse cell type communication within tissues and in living organisms. Building upon three-dimensional high-resolution optical data generation and computational algorithm development, optogenetics has emerged as an indispensable pillar for investigating cell and tissue structures, as well as manipulating four-dimensional changes in cell physiology. Noninvasive optical methods with the ability to monitor and manipulate cell physiology deep inside living tissues and the ability to tightly focus on specific targeted areas would greatly extend the use of optogenetics in vivo.

Recent progress in constructing closed-loop and deep-tissue optogenetic devices with drug delivery systems has provided novel means to test complex experimental designs in vivo in a minimally invasive manner. However, in vivo computational quantification of optogenetic readouts in a high-throughput fashion still remains challenging. Sophisticated software pipelines are yet to be developed to systematically incorporate precise information about experimental parameters, such as illumination intensity, area of acquisition, expression levels of the optogenetic toolkit and indicators, quantum yields, signal patterns, and intensity of readouts.

Optogenetic therapies are gaining momentum for translation into the clinical setting. Promising results have been reported from a growing number of preclinical optogenetic researches in disease intervention in animal models. Most encouragingly, optogenetic therapy has recently been applied to partially recover visual function in a blind patient diagnosed with retinitis pigmentosa. This medical milestone was achieved through a single-dose intraocular injection of an AAV vector encoding ChrimsonR (423), an engineered microbial opsin with its photoactivation window red-shifted to 595 nm (424) to minimize pupil constriction. Moreover, a red-shifted channelrhodopsin, ChRmine, has been used for deep-brain photoactivation of neurons to trigger perception without cranial surgery and implantation of optical devices (425, 426). Finally, with recent progress in designing light-switchable therapeutic immune cells OptoCAR-T and LiCAR-T cells (23, 303) and intrabodies (339–341), optogenetics promises to accelerate the development of personalized medicine, whereby the time, location, and duration of therapeutic effects can be tailored by light.

## GLOSSARY

4HBD: NH_2_-terminal four-helical bundle domain
AAV: Adeno-associated virus
Abl: Abelson tyrosine kinase
AdoCbl: 5′-Deoxyadenosylcobalamin
AFB2: Auxin signaling F-box 2 protein
AKT: RAC-alpha serine/threonine-protein kinase
Am1: Am1_c0023g2
AMBRA1: Activating molecule in Beclin 1-regulated autophagy protein 1
As: *Avena sativa*
AuLOV: Aureochrome LOV
AuNP: Gold nanoparticle
BACCS: Blue light-activated Ca^2+^ channel switch
BFUS: Brain-penetrant focused ultrasound
BLI: Biolayer interferometry
BLINK: Blue light-induced potassium channel
BphP1: Bacteriophytochrome photoreceptor 1
BRD4: Bromodomain-containing protein 4
BV: Biliverdin
bZIP: Basic region/leucine zipper
cAMP: Cyclic AMP
CAR: Chimeric antigen receptor
CaV: Voltage-gated Ca^2+^ channel
CB5: Cytochrome *b*5
CBD: Cobalamin-binding domain
CDR: Complementarity-determining region
Che: Chemotaxis histidine kinase
ChR2: Channelrhodopsin-2
CIB1: CRY-interacting bHLH
CIBN: NH_2_-terminal residues of CIB1
CLASP2: CLIP-associated protein 2
CLOC: Closed-loop optogenetic control
cLOV: LOV core domain
CNGCs: Cyclic nucleotide-gated channels
cODC1: COOH-terminal degron of murine ornithine decarboxylase
COP1: Constitutive photomorphogenesis protein 1
CP: Cas9-sgRNA plasmid
CPH1: Cyanobacterial phytochrome 1
Cph1S-o: Codon-optimized Cph1S
cpLOV2: LOV2 circular permutant
CRAC: Calcium release-activated calcium
CRISPR: Clustered regularly interspaced short palindromic repeats
CRISPRi: CRISPR interference
CRY: Cryptochrome
CTZ: Coelenterazine
DAG: Diacylglycerol
DAI: DNA-dependent activator of interferon (IFN)-regulatory factor
DC: Dendritic cell
dCas9: Catalytically dead Cas9
DrBphP: *Deinococcus radiodurans* phytochrome
DTZ: Diphenylterazine
EB1: End-binding protein
ECM: Extracellular matrix
EGFR: Epidermal growth factor receptor
eMag: Enhanced Magnet
EnvA: Avian sarcoma leucosis virus envelope protein
ePDZ: Engineered PDZ domain
ER: Endoplasmic reticulum
ERK: Extracellular signal-regulated kinase
FAD: Flavin adenine dinucleotide
FFz: Fluorofurimazine
FGF: Fibroblast growth factor
FHL: FHY1-like
FHY1: Far-red elongated hypocotyl 1
FKF1: Flavin-binding Kelch repeat F-box1
FLARE: Fast light- and activity-regulated expression
FLiCRE: Fast light- and Ca^2+^-regulated expression
FMN: Flavin mononucleotide
FP: Fluorescent protein
FTH1: Ferritin heavy chain
FTO: Fat mass and obesity-associated
FUS: Fused in sarcoma
Fz: Furimazine
GAF: cGMP phosphodiesterase/adenylyl cyclase/FhlA
GECA: Genetically encoded Ca^2+^ channel actuator
GEF: Guanine nucleotide exchange factor
GePGS: Genetically encoded photochromic guide star
GFP: Green fluorescent protein
GLuc: *Gaussia* luciferase
GPCR: G protein-coupled receptor
GSDMD: Gasdermin D
HFz: Hydrofurimazine
HKRD: Histidine kinase-related domain
HLH: Helix-loop-helix
HSV: Herpes simplex virus
HTH: Helix-turn-helix
Htr: Halobacterial transducer protein
iLID: Improved light-induced dimer
IMM: Inner mitochondria membrane
Interleukin 6: IL-6
IP: Inositol phosphate
IP_3_: Inositol trisphosphate
IR: Battery-powered infrared
JellyOp: Jellyfish opsin
KAT1: Inward-rectifier K^+^ channel subunit 1
*K*_d_
: Dissociation constant
KRAB: Krüppel-associated box
LACK: Light-activated CRISPRi using KRAB domain
LACP: Lipid-capsulated, AuNPs-condensed Cas9-sgRNA plasmid
LAPD: Light-activated phosphodiesterase
LiMETER: Light-inducible membrane-tethered peripheral endoplasmic reticulum
LiPD: Light-induced protein depletion
LiPOP1: Light-induced nonapoptotic tool 1
LITE: Light-inducible transcriptional effectors
LLPS: Liquid-liquid phase separation
LOCa: Light-operated Ca^2+^ channel
LOV: Light-oxygen-voltage
LOVTRAP: LOV2 trap and release of protein
LV: Lentivirus
MAPK: Mitogen-activated protein kinase
MAVS: Mitochondrial antiviral-signaling protein
MCAK: Mitotic centromere-associated kinesin
MCS: Membrane contact site
MKII: Calmodulin-binding peptide
MLKL: Mixed-lineage kinase domain-like pseudokinase
MLS: Mitochondrial leading sequence
MMP: Metalloproteinase
MOMP: Mitochondrial outer membrane permeabilization
MS2: MS2 coat protein
MT: Microtubule
MyD88: Myeloid differentiation primary response protein 88
nanoReD: Nanobody-based, red light-induced dimerization system
NES: Nuclear export signal
NIR: Near-infrared light
NLS: Nuclear localization signal
nMag: Negative magnet
NS3: Nonstructural 3
OESS: Optoelectronic stimulation and sensing
OMM: Outer mitochondrial membrane
OPM: Output module
OPN4: Olivary pretectal nucleus 4
Opto-MOR: Light-sensitive μ-opioid-like receptor
Opto-XR: Synthetic light-gated GPCR
Optobodies: Optogenetically activatable intracellular antibodies
OptoDyCE: All-optical dynamic cardiac electrophysiology framework
OptoPB: Photosensitive polybasic domain
ORAI: Calcium release-activated calcium modulator
ORF: Open reading frame
PA-Cre: Photoactivatable Cre recombinase
PAC: Photoactivated adenylyl cyclase
paCBE: Photoactivatable cytosine base editor
PAMEC: Photoactivatable RNA N^6^-methyladenosine (m6A) editing system
PAS: Per-ARNT-Sim
PCB: Phycocyanobilin
pd: Dronpa1Photo-dissociable dimeric Dronpa 1
PDGFR: Platelet-derived growth factor receptor
PDMS: Polydimethylsiloxane
PhoCl: Photocleavable protein
PHY: Phytochrome-specific domain
PhyB: Phytochrome B
PI: Phosphoinositide
PI-Rac1: Photo-inhibitable Rac1
PI-Src: Photo-inhibitable Src
PI3K: Phosphatidylinositol 3-kinase
PIF: Phytochrome-interacting factor
PixELL: Pix Evaporates from Liquid-like droplets in Light
PLC: Phospholipase C
PM: Plasma membrane
pMag: Positive Magnet
POI: Protein of interest
PRC1: Protein regulator of cytokinesis 1
Psd: Photosensitive degron
PSM: Photosensory module
PtAU1a: Phaeodactylum tricornutum aureochrome 1a
PTS1: Peroxisome targeting sequence
PYP: Photoactive yellow protein
PΦB: Phytochromobilin
QRB: Quantum reference beacon
RbV: Rabies virus
REDMAP: Red/far-red light-mediated and miniaturized Δphytochrome A (ΔPhyA)-based photoswitch
RET: Rearranged during transfection
RF: Radiofrequency
RGK: Ras-like GTPases Rad/Rem/Gem/Kir
RGS: Regulator of G protein signaling
RsLOV: LOV domain from *Rhodobacter sphaeroides*
RTK: Receptor tyrosine kinase
RV: Retrovirus
ScFV: Single-chain variable fragment
SLAIN2: SLAIN motif-containing protein 2
SMOC: Supramolecular organizing centers
SOS: Son of sevenless guanine nucleotide exchange factor
SR: Sensory rhodopsin
STAS: Sulfate transporter anti-sigma-factor antagonist
STIM: Stromal interaction molecule
STZ: Selenoterazine
SUMO: Small ubiquitin-like modifier protein
TAF15: TATA-binding protein-associated factor 2N
TALE: Transcription activator-like effector
TCR: T-cell receptor
TET: Ten–eleven Translocation
tetO: Tetracycline operator
TEVcs: Tobacco etch virus cleavage site
TFEB: Transcription factor EB
TIP1: tax-interacting protein-1
TRE: Tetracycline response element
TRIM21: Tripartite motif 21
TrkB: Tropomyosin receptor kinase B
TROVE: Time reversal of variance-encoded light
TRPC: Transient receptor potential C
TRUE: Time-reversed ultrasonically encoded
tTA: Tetracycline-controlled transactivator
TULIP: Tunable, light-controlled interacting protein tag
UASG: Upstream activating sequence of Gal
UCNP: Upconversion nanoparticle
UGI: Uracil DNA glycosylase inhibitor
uTEVp: Ultra TEV protease
UV: Ultraviolet
UVR8: UV resistance locus 8
VfAU1: *Vaucheria frigida* aureochrome 1
VHH: Single variable domain on a heavy chain
VPR: VP64-p65-Rta
VVD: Photoreceptor vivid
Zdk: Zdark
αTAT: α tubulin acetyltransferase
ΔΨ_m_: Inner membrane potential
µLED: MicroLED

## GRANTS

This work is supported by grants from the National Institutes of Health (R01GM112003 to Y.Z., R01HL134780 to Y.H., R01HL146852 to Y.H., and R01CA240258 to Y.H.), the Welch Foundation (BE-1913-20190330 to Y.Z.), the Cancer Prevention and Research Institute of Texas (RP210070), and the American Cancer Society (RSG-16-215-01-TBE to Y.Z. and RSG-18-043-01-LIB to Y.H.).

## DISCLOSURES

No conflicts of interest, financial or otherwise, are declared by the authors.

## AUTHOR CONTRIBUTIONS

P.T. and Y.Z. conceived and designed research; P.T. analyzed data; P.T., L.H., and Y.Z. prepared figures; P.T., L.H., and Y.Z. drafted manuscript; P.T., L.H., Y.H., and Y.Z. edited and revised manuscript; P.T., Y.H., and Y.Z. approved final version of manuscript.
